# Pancreatic Beta Cell G-Protein Coupled Receptors and Second Messenger Interactions: A Systems Biology Computational Analysis

**DOI:** 10.1371/journal.pone.0152869

**Published:** 2016-05-03

**Authors:** Leonid E. Fridlyand, Louis H. Philipson

**Affiliations:** Departments of Medicine and Pediatrics, the Kovler Diabetes Center, The University of Chicago, Chicago, IL, 60637, United States of America; NIDCR/NIH, UNITED STATES

## Abstract

Insulin secretory in pancreatic beta-cells responses to nutrient stimuli and hormonal modulators include multiple messengers and signaling pathways with complex interdependencies. Here we present a computational model that incorporates recent data on glucose metabolism, plasma membrane potential, G-protein-coupled-receptors (GPCR), cytoplasmic and endoplasmic reticulum calcium dynamics, cAMP and phospholipase C pathways that regulate interactions between second messengers in pancreatic beta-cells. The values of key model parameters were inferred from published experimental data. The model gives a reasonable fit to important aspects of experimentally measured metabolic and second messenger concentrations and provides a framework for analyzing the role of metabolic, hormones and neurotransmitters changes on insulin secretion. Our analysis of the dynamic data provides support for the hypothesis that activation of Ca^2+^-dependent adenylyl cyclases play a critical role in modulating the effects of glucagon-like peptide 1 (GLP-1), glucose-dependent insulinotropic polypeptide (GIP) and catecholamines. The regulatory properties of adenylyl cyclase isoforms determine fluctuations in cytoplasmic cAMP concentration and reveal a synergistic action of glucose, GLP-1 and GIP on insulin secretion. On the other hand, the regulatory properties of phospholipase C isoforms determine the interaction of glucose, acetylcholine and free fatty acids (FFA) (that act through the FFA receptors) on insulin secretion. We found that a combination of GPCR agonists activating different messenger pathways can stimulate insulin secretion more effectively than a combination of GPCR agonists for a single pathway. This analysis also suggests that the activators of GLP-1, GIP and FFA receptors may have a relatively low risk of hypoglycemia in fasting conditions whereas an activator of muscarinic receptors can increase this risk. This computational analysis demonstrates that study of second messenger pathway interactions will improve understanding of critical regulatory sites, how different GPCRs interact and pharmacological targets for modulating insulin secretion in type 2 diabetes.

## Introduction

**Fig 1:**
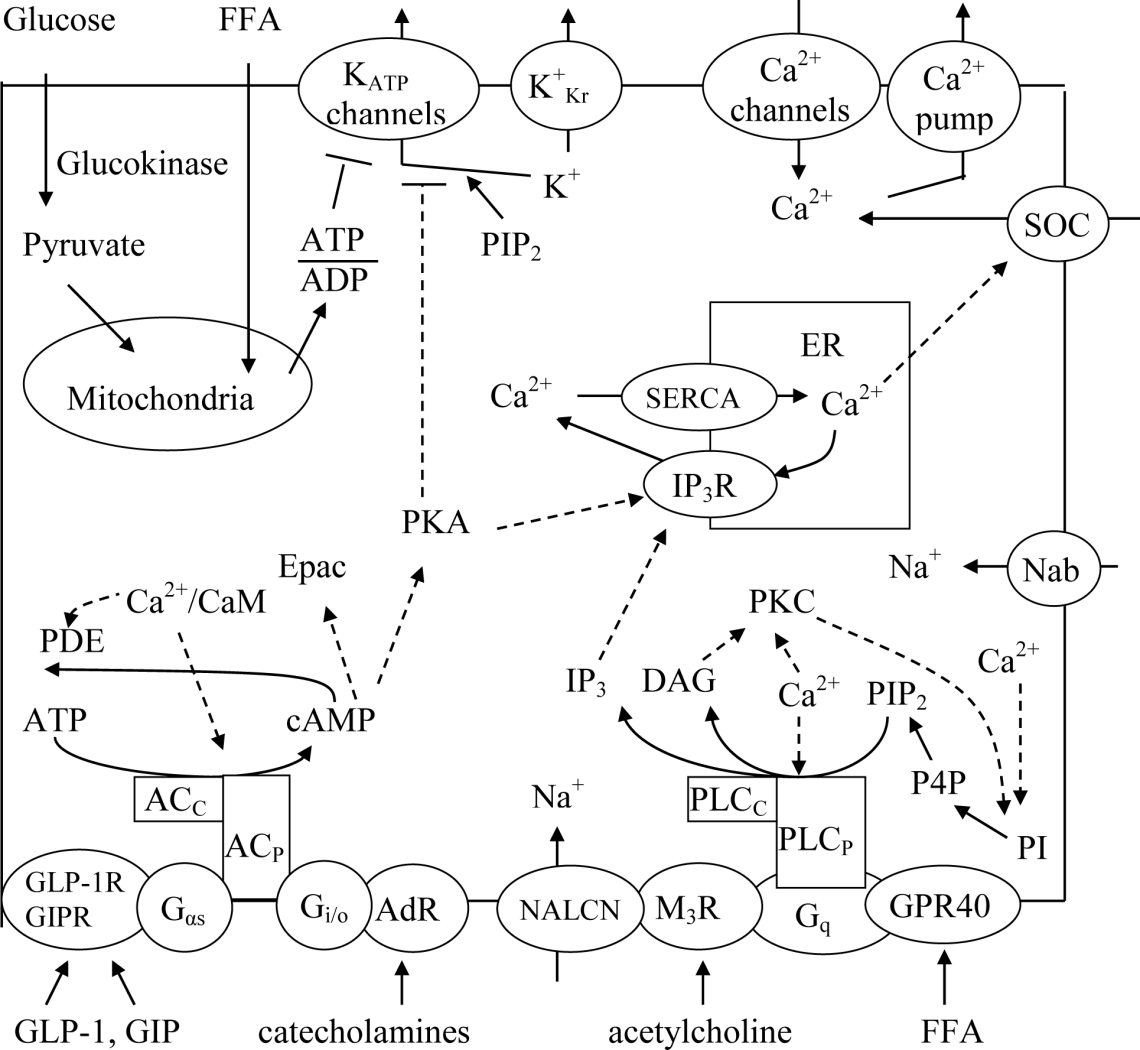
A schematic model of the main signaling pathways that regulate insulin secretion. Glucose enters the cell through its glucose transporter, and is phosphorylated and metabolized in the mitochondrion as well free fatty acids (FFA). Glucose and FFA metabolism leads to an increase in the ATP/ADP ratio, closure of the ATP-sensitive potassium (K_ATP_) channels leading to plasma membrane (PM) depolarization that increase a calcium influx through the voltage-gated calcium channels and an increase in cytoplasmic Ca^2+^. Other transmembrane channels can also regulate PM potential: K^+^_Kr_ is the voltage gated K^+^ channel, SOC is the store operating channels, Nab is the Na^+^ background current, NALCN is the specific nonselective cation channel that can be modulated by acetylcholine through activation of M_3_ muscarinic receptors (M_3_R). Increase in cytoplasmic Ca^2+^ leads to the activation of several calcium dependent enzymes including adenylyl cyclase (AC) and phospholipase C (PLC). Phosphoinositides pathway: phosphatidylinositol-4-phosphate (P4P) is synthesized from phosphatidylinositol (PI) by phosphatidylinositol 4-kinases and its synthesis activates by PKC and Ca^2+^. Phosphotidylinositol-4,5-bisphosphate (PIP_2_) in turn is primarily formed from P4P by phosphatidylinositol-4-phosphate 5-kinase I. cAMP pathway: Ca^2+^/CaM is Ca^2+^-bound calmodulin, Synthesis and degradation of cAMP are catalyzed by adenylyl cyclase and phosphodiesterase (PDE), respectively. AC_c_ is the soluble AC, AC_P_ is the G-protein controlled AC on plasmalemma that can be activated by stimulatory G_αs_ type G-protein and Ca^2+^/CaM and deactivated by inhibitory G_i/o_ type G-protein. PDE activity can be enhanced by Ca^2+^/CaM, cAMP activates protein kinase A (PKA) and exchange protein (Epac). The incretins glucagon-like peptide-1 (GLP-1) and glucose-dependent insulinotropic polypeptide (GIP) bind to their respective receptors (GLP-1R and GIPR), activate AC_P_ and increase intracellular levels of cAMP. Endogenous catecholamines adrenaline (epinephrine) and noradrenaline (norepinephrine) bound with G-protein-coupled α_2A_-adrenergic receptors (AdR) on plasma membrane and inhibit AC_P_. Phospholipase C (PLC) pathway. PLC_C_ is the cytoplasmic calcium activated PLC and PLC_P_ is plasma membrane bound PLC that can be activated by G_q_ type G-protein. Acetylcholine and FFA can bind to their respective receptors (M_3_R and FFAR1/GPR40) expressed at the cell surface and trigger a Ga_q_-mediated activation of PLC_P_. PLC_C_ and PLC_P_ generate inositol-3-phosphate (IP_3_) and diacylglycerol (DAG) by hydrolyzing membrane PIP_2_. DAG activates protein kinase C (PKC). Endoplasmic reticulum (ER): SERCA is the endoplasmic reticulum Ca^2+^ATPase, IP_3_R is the IP_3_ receptor; that can be activated by IP_3_ and PKA. Solid lines indicate fluxes, and dashed lines indicate inhibitory or stimulatory influences on currents or fluxes.

Insulin release from the pancreatic β-cells must respond acutely to meet the insulin demands of the organism. However, in type 2 diabetes (T2D) pancreatic β-cells fail to compensate for an increase in blood glucose concentration with sufficient insulin secretion, leading to progressive hyperglycemia [1]. T2D is a chronic metabolic illness with dramatic increasing medical and financial costs but prevention and effective treatments remain suboptimal. Numerous studies have been published on the regulation of β-cell function. A general reaction network diagram for the β-cell is shown in Fig 1.

Glucose is the major physiologic regulator of insulin release. Glucose-stimulated insulin secretion (GSIS) includes an increase in ATP/ADP ratio leading to a closure of ATP sensitive potassium (K_ATP_) channels, plasma membrane (PM) depolarization, opening of voltage-gated calcium channels(VGCC) with corresponding calcium influx and an increased cytosolic Ca^2+^. The rise in intracellular free calcium concentration ([Ca^2+^]_c_) is an important signal in the initiation of β-cell insulin secretion [2–4]. The β-cell has numerous G protein coupled receptors (GPCRs) that can activate or inhibit β-cell insulin secretion [5]. Therefore a better understanding of how activation of GPCRs regulate β-cell function might illuminate approaches to help β-cell compensation and lead to better approaches to treatment of T2D.

Additional regulation of insulin release is provided by circulating metabolic secretagogues and by stimuli such as hormones and neurotransmitters. This permits close regulation of islet hormone secretion. For example, non-metabolic stimulation of insulin release occurs during the first phase of feeding and precedes any increase in blood glucose (termed the "cephalic phase"). This is largely mediated by the release of acetylcholine from nerves innervating pancreatic islets and the cholinergic stimulation of the muscarinic acetylcholine receptors [3, 6–8]. Incretin hormones released from gastrointestinal L-cells in response to food intake also stimulate insulin secretion [9]. On the other hand the neurotransmitters such as noradrenaline inhibit insulin secretion to increase glucose availability during times of stress [10]. These signals are mediated by a variety of GPCRs that have complimentary or antagonistic actions on insulin secretion [5, 11]. Interestingly, signaling networks must convert a large variety of extracellular stimuli onto a limited number of intracellular second messenger pathways. This includes intracellular free Ca^2+^ concentration and the two main signals of activated GPCRs: cyclic AMP (cAMP) on the one hand and inositol 1,4,5-trisphosphate (IP_3_) and diacylglycerol (DAG) on the other [3, 12, 13]. Group of third pathways through adhesion class GPCR was also found in islets [14]. However, these pathways were only beginning to be studied in β-cells and there is not enough data to include them here. There is therefore considerable interest in understanding how GPCRs in β-cells integrate second messenger signaling.

Despite the recent increase in our knowledge of β-cell physiology and biochemistry, we still lack a coherent model of how second messengers and their interactions regulate β-cell secretion. The complex nature of the receptors and second messenger interactions makes it extremely difficult to understand how they are regulated and which parameters determine their dynamics. However, systems biology has emerged to provide a systematic approach to integrate the complexity of cellular signaling combining dynamic experiments with a computational biology approach [15–17]. Mathematical modeling has been used extensively and with great success to study metabolic and signaling networks [16, 18, 19]. This approach is useful for describing experimental data, deducing regulatory principles, and understanding complex dynamic phenomena such as oscillations. Integrated complex models of signal transduction pathways were published recently for synaptic input studies [20], mouse hepatocytes [21], cardiac myocytes [22] and liver metabolism [15]. However, this approach has not been as well developed for pancreatic β cells. Indeed only GLP-1 receptor coupled G-protein regulated cAMP signaling has been modeled previously [23–28].

We have previously focused on applying this mathematical modeling approach to mechanisms of glucose sensing of insulin secretion, Ca^2+^ and cAMP dynamics, electrophysiology events and exocytosis in pancreatic β-cells [23, 25, 29–34]. Here the objective is to evaluate the effect of interactions of metabolites, hormones, GPCRs and second messengers in regulation of insulin secretion in the pancreatic β-cell. For this aim we have constructed an integrated mathematical model of interaction of these components based on the framework developed in our previous models. In this work we focus on those cellular signaling pathways that appear essential for relatively rapid β-cell responses. We have estimated individual reaction rates and model parameters by fitting the theoretical reaction scheme to a variety of key experimental findings published to date in both β-cells and insulinoma cell lines. The model gives a reasonable fit to important aspects of experimentally measured metabolic, plasma membrane potential and second messenger concentrations and provides a framework for analyzing the role of metabolic, hormones and neurotransmitters changes on insulin secretion. This allowed us to refine the model to test hypotheses and conclusions about interacting pathways to design *in silico* experiments.

We analyzed interactions of clinically relevant GPCR agonists. We included those responding to the neurotransmitters acetylcholine and catecholamines with stimulation and inhibition of insulin secretion, respectively, to the incretin hormones GLP-1 and GIP with potentiation of GSIS, and the agonists for the free fatty acid (FFA) receptor (e.g., FFAR1/GPR40) and determined how combinations of different agonists affect second messenger dynamics in β-cell.

Defective regulation of messenger pathways clearly contributes to T2D in a variety of ways (see below). For this reason new anti-diabetic drugs in use and in development exploit several β-cell stimulating GPCR pathways in order to combat the growing health and economic costs of T2D. However, the effects of agonist combinations on the tightly coupled metabolic and signaling pathways of β-cells as well as on insulin secretion in diabetic states have not been carefully studied. We have attempted to analyze these processes and evaluate how particular impairments in the mechanisms of β-cell regulations sensing can lead to insulin release changes in T2D. Here we also examined the hypothesis that simultaneous stimulation of multiple signaling pathways is potentially is inhibitory for β-cell function and insulin secretion, but instead found that for the most part that combined stimulation can be synergistic.

## Models and Methods

Here we will briefly outline the different parts of the regulatory mechanisms in β-cell. Fig 1 shows a schematic diagram of the biochemical steps, Ca^2+^ handling, channels, G-protein-coupled-receptors and mechanisms of second messenger regulation that were used for our mathematical modeling. Details of modeling are described below in the section “Computational model” (CM).

### Metabolic regulation

The cellular metabolic mechanisms leading to insulin secretion in pancreatic β-cells are fairly well understood. Glucose-dependent signal transduction begins with uptake of glucose into β-cells via the glucose facilitative transporter. Glucose molecules are rapidly phosphorylated by glucokinase and converted to pyruvate in the cytosol via the glycolytic pathway, and pyruvate and FFA are oxidized within the mitochondria producing ATP. Blocking K_ATP_ channels by an ATP/ADP-dependent mechanism initiates plasma membrane depolarization to the threshold potential for leading to Ca^2+^ influx through VGCCs and increases free cytosolic calcium concentration ([Ca^2+^]_c_). The rise in [Ca^2+^]_c_ is a key signal in the initiation of -cell insulin secretion (for reviews, see [2, 3, 32]). Other metabolic cofactors such as NADH or NAD(P)H have also been considered as possible coupling candidates as well [32, 35]. We used a simplified coarse-grained mathematical model of these mechanisms for pancreatic β-cells that is based on previous results [31, 32] (see CM).

The effects of FFAs on insulin secretion can also due to simply supplying fuel for cell metabolism [36] and we include this role for FFAs in our model as an increase in ATP/ADP ratio following FFA challenge (see CM). However, we do not consider this mechanism in detail here, because in physiological conditions FFA concentration in blood is low so that FFA acts mainly through FFA activated GPCRs [37] and only this signaling FFA effect is considered here.

### Channels and regulation of plasma membrane potential

Plasma membrane potential (V_p_) regulates Ca^2+^ influx through VGCCs in β-cells. On other hand V_p_ is regulated by ion channels and pumps. A schematic diagram of the principle β-cell channels (Fig 1) includes the more completely characterized channels and pumps. Additional actions such as activation of ion channels by second messengers also have been described [38, 39] and considered in our model (see CM).

β-cells display spike activity in response to glucose, an effect that has been modeled previously [29, 30, 40]. However, changes in PM potential and their corresponding voltage-dependent currents are considerably faster during spikes (milliseconds) than the changes in second messenger concentrations (minutes). For this reason, we excluded fast changes of PM potential here and for simplicity we did not describe ionic spike activity, modeling the time-dependent gating variables for currents as the stationary voltage dependences.

#### K^+^ channels

We have included the ATP-sensitive potassium (K_ATP_) channel and the voltage gated K^+^ (K^+^_Kr_) channel. Messengers can affect K^+^ channel dynamics in various ways. We have taken into account that protein kinase A (PKA) activation and a decrease in phosphatidylinositol 4,5-bisphosphate (PIP_2_) concentration can lead to block of K_ATP_ channels [38, 41, 42] (see CM for details). Several studies suggest that voltage-gated K^+^ channels might be also regulated by PIP_2_ [43, 44]. However, K_v_2.1 channels, that are the main voltage-gated K^+^ channels in β-cells [2, 4] are not sensitive to PIP_2_ depletion [43]. For this reason we excluded the influence of PIP_2_ on voltage-gated K^+^ channels.

#### Ca^2+^ channels and pumps

PM depolarization potentiates glucose-induced Ca^2+^ influx through voltage-gated Ca^2+^ channel (VDCCs). Depletion of intracellular Ca^2+^ stores in β-cells activates a special Ca^2+^-release activated current (or store operating current (SOC)). While the detailed mechanism for coupling of ER Ca^2+^ store depletion with these PM channels activation is uncertain, it likely involves translocation of STIM1 and STIM2 proteins from the ER calcium stores to the PM where they interact with ORAI1 and related proteins associated with cation influx channels [45, 46]. Plasma membrane Ca^2+^ pumps provide an outward Ca^2+^ current.

#### Na^+^ channels

Voltage-gated Na^+^ current is likely inactivated in mouse β-cells [47] due to the relatively high resting V_p_, so it is not considered, although it may play a role in human beta cells (see [30]). Nonselective PM cation channels permeable to Na^+^ and Ca^2+^ have been described in pancreatic β-cells, and the transient receptor potential ion channels were proposed for this role [48, 49]. Specific nonselective cation channels (NALCN) are expressed in pancreatic islets and can also be modulated by acetylcholine through activation of M_3_ muscarinic receptors [39, 50]. We modeled these currents as Na^+^ background current (*I*_Nab_) through specific channels on PM (see CM).

### Receptors, G-proteins and messenger regulation

Regulating signals from hormones and neurotransmitters are mediated by a variety of G-protein-coupled-receptors (GPCRs) that bind ligands from the extracellular space. GPCRs couple to stimulatory and inhibitory membrane-bound heterotrimeric G-proteins, mediating regulation of insulin secretion by the second messenger pathways. Activation of most GPCRs occurs in a similar time frame, but downstream effects may decay with a slower time course and may also effect gene expression.

Two main types of activation were found for different receptors: 1. Collision coupling, where a ligand binds to the free receptor and then the ligand-receptor complex ‘‘collides” with the free G-protein. 2. Pre-coupling, where stable receptor/G-protein complexes pre-exist and a ligand can bind with these complexes. There is accumulating evidence for both collision coupling and pre-coupling of GPCRs [51, 52].

Although there are many different GPCRs for the specific type of receptors there is a shared mechanism of activation. Following interaction with activated receptor the heterotrimeric G-proteins catalyze the exchange of GDP for GTP on the α-subunit. This event triggers conformational and/or dissociation events between the α-subunit and βγ-subunit. Both Gα-GDP and Gβγ subunits then activate (or inhibit) downstream signaling molecules (enzymes, kinases and ion channels) and thereby elicit cellular responses. The activation cycle is terminated by the Gα intrinsic GTPase activity which allows GTP hydrolysis and the reassociation of Gα-GDP with Gβγ subunits so to restore the inactive basal state. Then the G-protein system initiates a new cycle [53](see CM).

Desensitization is an important mechanism of regulation of receptor activity. At the receptor level several processes of desensitization have been shown to play a role in limiting signal duration and intensity [54]. Desensitization of GPCRs occurs with three phases: the phosphorylation of the receptor bound to ligand (activated receptor) by G-protein-coupled receptor kinases, sequestration/internalization and down-regulation or return to the surface [55]. We will consider the processes of desensitization for each receptor. However, we do not consider how specific processes result in desensitization, such as the SUMOylation of the GLP-1 receptor in response to glucose we have previously described [56] or phosphorylation [57]. We also did not consider synthesis, degradation or irreversible translocation of receptors in the model. The multiple dephosphorylation/recycling steps were shortened into one single reaction (for details see CM).

Constitutive receptor activity has been observed to occur in many different GPCRs (“tonic receptor activation”) and can be explained by different mechanisms [58–60]. On other hand, islet α-cells can also secrete GLP-1 [61] and it may be that the neurotransmitter acetylcholine can be secreted by human alpha cells [62]. FFAs that activate FFAR1/GPR40 are present in blood and vary following meals. All these data provide reason enough to suggest that some activation of GPCRs takes place even without an additional GPCR agonist. For simplification, we have employed low concentrations of receptor ligands in modeling of experiments even where no specific GPCR agonists were used (for details see CM).

### cAMP pathway

Signal routing to cAMP involves multiple GPCRs that regulate activity of several isoforms of AC and phosphodiesterases leading to production and hydrolysis of cAMP (Fig 1). cAMP activates both PKA and the cAMP-regulated guanine nucleotide exchange factor (Epac). We previously developed a computational model of the pancreatic β-cell cAMP pathway [23, 25] that was used in the construction of the general model here (see CM).

#### Incretin hormones and receptors in β-cells

Glucagon-like-peptide-1 (GLP-1) is one potent incretin hormone coupled primarily to the specific α_s_-G-protein-coupled receptor (GLP-1R). The GLP-1R belongs to the class B family of GPCRs. The GLP-1R couples to the G-protein complex and facilitates the release of the activated Gα_s_ subunit of the complex, which activates plasma membrane-bound adenylyl cyclase (AC_P_) to produce cAMP [12, 63, 64]. Following agonist-induced receptor activation, GLP-1R is probably internalized [65]. Small ubiquitin-related modifier protein (SUMO) can covalently modify GLP-1R and thereby desensitize GLP-1 signaling [56].

In islets, GLP-1R is expressed predominantly on β-cells [66]. A mathematical model for GLP-1R signal transduction was published recently [24]. However, this model lacks components such as a complex of G-protein with AC_p_. To address this we used our general model for GPCR effects (for details see CM).

Another important incretin hormone is glucose-dependent insulinotropic polypeptide or gastric inhibitory polypeptide (GIP). GIP is a 42-amino-acid hormone, secreted from the enteroendocrine K cells and one of the major mediators in the regulation of nutrient-dependent insulin release from the pancreas [67]. Hyperglycemic clamp studies or GIP infusion established that GIP was insulinotropic at physiological concentrations [68]. GIP exerts its effects through binding to a specific α_s_-G-protein-coupled receptor (GIPR) that also belongs to the B-family class of GPCRs of the glucagon–secretin family of peptides [68, 69].

The GIPR undergoes rapid and reversible homologous desensitization following binding of GIP [68, 70]. Regulator of G-protein signaling-2, G-protein receptor kinase 2, and β-arrestin 1 all have been implicated in GIPR desensitization [71].

Interestingly, class B GPCRs are characterized by a common topology of the ligand-receptor complex, and by their ability to couple multiple G-proteins. Biochemical and structural studies have led to a model of class B1 GPCR activation by peptide hormones including GLP-1 and GIP, referred to as the “two-step” mechanism [72]. This corresponds to collision coupling, where an agonist binds to the free receptor and then the agonist-receptor complex ‘‘collides” with the free G-protein. Corresponding models were developed for GLP-1R and GIPR (see CM).

#### Adrenergic receptors

Adrenergic agonists, the endogenous catecholamines adrenaline (epinephrine) and noradrenaline (norepinephrine), as well as agonists such as clonidine, all inhibit insulin secretion [10, 73, 74]. G-protein-coupled α_2A_-adrenergic receptors (AdR) are responsible for these effects for adrenaline/noradrenaline, and they are mediated by the pertussis toxin-sensitive heterotrimeric G_i_ and G_o_ proteins, that inhibit AC [10, 73]. The mRNAs encoding the α_2A_ adrenoceptor are expressed at relatively high levels in human islets [5].

Co-immunoprecipitation studies showed pre-coupling of a_2A_-adrenergic receptors with G_i/o_ proteins [75]. However, no evidence for a_2A_-adrenergic receptor and G_i_-protein precoupling was found in resonance energy transfer studies and it was suggested that a_2A_-adrenergic receptors and G-proteins interact by rapid collision coupling [76]. For this reason we also used the collision coupling mechanism for α_2A_-adrenergic receptor (see CM). Desensitization mechanisms have not been specifically studied in pancreatic β-cells for these receptors, however, these receptors recycle rapidly in COS-7 and HEK-293 cells [77].

We modeled the processes of desensitization of GLP-1R, GIPR and AdR as a simplified mechanism that includes only one step for the active state (where a receptor is bound with ligand) pass off or recirculation of GPCRs (see CM), because these mechanisms are not well studied.

#### Adenylyl cyclases and phosphodiesterases

Activated adenylcyclase (AC) synthesizes cAMP from the substrate Mg^2+^ATP. Rodent and human β-cells and insulinoma cell lines express different AC isoforms, including the Ca^2+^ and calmodulin-activated isoforms AC1, AC3, and AC8 [78–81]. Interestingly, among the cloned and characterized members of the mammalian AC family, two isoforms, AC1 and AC8, are synergistically activated by Ca^2+^-calmodulin and G_sα_ [82, 83]. Both receptors for GIP and GLP-1 activate the α subunit (G_sα_) of the G-protein leading to an activation of AC in pancreatic cell lines and isolated pancreatic islets [67, 84]. The activation of AC by the α subunit (G_sα_) of the G-protein may be associated with formation of their complex with PM bound AC (AC_p_) [85, 86].

**Fig 2:**
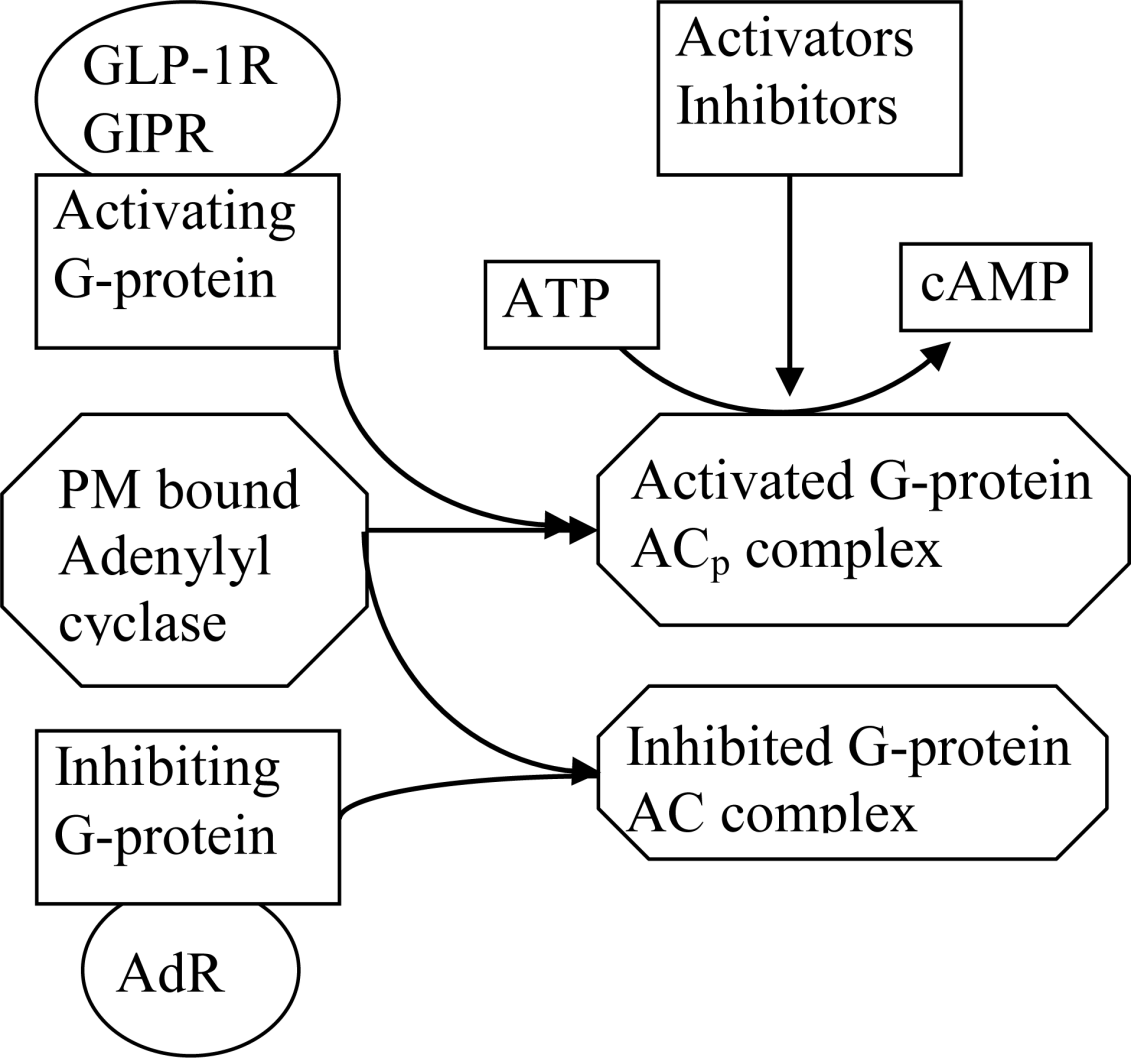
A schematic model of interaction of G-proteins activated by receptors with PM bound adenylyl cyclase (AC_p_). Activating G-protein can bind with AC_p_ and their complex can accelerate cAMP production. AC_p_ complex bounded with inhibiting G-protein cannot catalyze cAMP production.

GLP-1 resulting in increments in G_sa_ has been proposed to regulate calmodulin-activated AC8 that is central to GLP-1 signaling in rodent and human β-cells [78, 79, 87]. Our previous kinetic analysis supports this conclusion [23]. Similar interactions with AC8 was suggested for GIPR [68]. Catecholamines inhibit AC, however it is not known which isoform of AC in the β-cell is inhibited [10]. Activation of G_i_ by catecholamines may block AC_P_ mediated cAMP synthesis thus preventing the augmentation of insulin release stimulated by GLP-1 and GIP [10]. Acute activation of G_i/o_-coupled receptors leads to inhibition of AC8 isoform [88]. Therefore we suggest that this G_i_ binds to the same AC isoform as G-proteins activated by GLP-1 or GIP, but AC bound with G_i_ has no catalytic activity. We found that these mechanisms of interaction between G-proteins and target enzymes (represented for AC_p_ in Fig 2) can play an important role in the regulation of messenger pathways (see Results and discussion).

Some soluble cytoplasmic adenylate cyclases (AC_c_) can also contribute to cAMP synthesis that are not controlled by G-proteins and forskolin but rather by calcium, ATP and bicarbonate [89]. Therefore alongside G-protein activated or inhibited AC_p_ we modeled a cytoplasmic AC_c_ that is insensitive to G-protein and can be activated by glucose and/or Ca^2+^ (see for detals CM).

Some resting cAMP concentration exists in the absence of agonists (see below). Mechanisms of AC activation were not studied in this case for β–cells. We suggest that AC activity in this case can be a consequence of basal activity of AC_c_ and constitutive G-protein dependent receptor for AC_P_ that is observed in other GPCRs.

Phosphodiesterases (PDEs) hydrolyze cAMP and cyclic GMP. There are eleven known PDE families that differ in primary structures, responses to specific effectors and sensitivities to specific inhibitors, cellular expression and intracellular location and may modulate distinct regulatory pathways within the cell [90, 91]. In β-cells, it has been suggested that several PDE isoforms (1C, 3B, 4, 8B, and 10A) are involved in regulation of insulin secretion [91, 92]. In our model we consider both Ca^2+^ bound calmodulin activated and constitutive PDE activity as in a previous model [23] (see CM).

#### PKA and Epac

The two primary downstream effectors of cAMP are PKA and the cAMP-regulated guanine nucleotide exchange factors (Epac) that play a critical role in insulin release [64, 93]. PKA is a holoenzyme composed of catalytic and regulatory subunits. The catalytic subunits release when cAMP binds to the regulatory subunits and then act to phosphorylate downstream substrates. Epac is similarly activated by cAMP [64, 93]. We have used a simple mathematical model of these events [25]. There are multiple phosphorylation targets of PKA and Epac that are related to their effects on exocytosis. PKA and Epac pathways can have different physiological relevance based on differences in cAMP binding constants and downstream targets [94]. However, in the present model, downstream targets of PKA and Epac include only PKA blocking of K_ATP_ channels (see Eq 13, CM) and regulation of the IP_3_ receptors in the ER (see CM).

### Phosphoinositides

Numerous phosphoinositides are now accepted as independent signaling molecules [95]. We will consider here phosphoinositides for β-cell physiology (see CM). Phosphatidylinositol-4-phosphate (P4P) is synthesized from phosphatidylinositol (PI) by phosphatidylinositol 4-kinases (PI4Ks) and its synthesis activates by PKC and Ca^2+^ [96]. Phosphotidylinositol-4,5-bisphosphate (PIP_2_) in turn is primarily formed from P4P by phosphatidylinositol-4-phosphate 5-kinase I [97] (Fig 1).

Despite being a small component of the plasma membrane, PIP_2_ has many diverse and critical roles in β-cell physiology. PIP_2_ serves as a precursor for the messenger molecules inositol-1,4,5-trisphosphate (IP_3_) and diacylglycerol (DAG) generated following activation of PLC. Membrane content of phospholipids, in particular PIP_2_, can also determine the sensitivity of the K_ATP_ channels. Particularly, the K_ATP_ channels became more resistant to ATP-induced closure as the membrane content of PIP_2_ was increased [98, 99]. A decrease in the PM PIP_2_ concentration may lead to K_ATP_ channels closure and corresponding PM depolarization [41]. We have developed a mathematical model for phosphoinositide dynamics where PIP_2_ can activate K_ATP_ channels (see Eq 10 in CM) and serves as a precursor for IP_3_ and DAG (see CM).

### Phospholipase C pathway

An important component of islet cell signal transduction and the control of islet function is innervation. Importantly, the modulators of insulin secretion include acetylcholine released from pancreatic parasympathetic nerve endings or possibly from alpha cells that activates phospholipase C (PLC) [3, 62, 100]. In general, over 50 hormone receptors can couple to the specific PLC-coupled G-proteins and some receptor tyrosine kinases can also stimulate PLCs that catalyze the formation of DAG and IP_3_ from PIP_2_ [101–103].

#### PLC pathway receptors

Pancreatic β cells express various G_q_-coupled receptors, including the muscarinic receptors, FFAR1/GPR40, GPR120 and different P2Y receptor subtypes, that can regulate PLC activity [67]. However, here we included only what seem to be the most important for the β-cell: muscarinic acetylcholine receptor (MR) and FFAR1/GPR40. Others of this class seem to be expressed at lower levels [5, 104].

#### Muscarinic acetylcholine receptor (MR)

Acetylcholine binds to MRs in the β-cell membrane coupled to Gα_q/11_ to activate PLC [3]. mRNA encoding the type 3 MR is the most abundant in human islets but other isofroms are expressed in β-cells [5].

Initially, it was thought that MR and G-proteins interact freely with each other on the cell membrane [105]. However, type 3 MRs can pre-couple with their preferential classes of G-proteins [51] and here we used a pre-coupled model (see CM). Agonist-bound MR are phosphorylated by G-protein-coupled receptor kinases that leads to receptor internalization and recycling (or down regulation) [55].

#### Free fatty acid receptor (FFAR1/GPR40)

The class A G-protein-coupled receptor GPR40, now also known as free fatty acid receptor 1 (FFAR1) is predominantly expressed in mouse, rat and human pancreatic β-cells and plays a major part in fatty acid amplification of GSIS [106]. FFAR1/GPR40 is activated by medium- to long-chain fatty acids and it is predominantly coupled to Gα_q_ that typically signals through PLC-mediated hydrolysis of membrane phospholipids and is coupled to the formation of IP_3_ and DAG [5, 106]. A collision coupling mechanism was suggested as the predominant mechanism for FFAR1/GPR40 (see [107]). Interestingly, investigations of β cell-specific inactivation of the genes encoding the Gα_q/11_-coupled receptor have shown that glucose does not directly activate this receptor [108]. In stably transfected HEK-293 cells, FFAR1/GPR40 receptors underwent rapid agonist-induced internalization [109]. However, in the absence of specific data we used a general mechaism for modeling the processes of desensitization for MR and FFAR1/GPR40 in β-cells (see CM).

#### Phospholipase C (PLC)

At least thirteen different PLC family members have been identified in various mammalian tissues and the consensus is that PIP_2_ is the major substrate of PLC yielding DAG and IP_3_ [110, 111]. Mouse and rat islets have been reported to express members of the three major phospholipase (PLC) subfamilies; PLC-β1,—β2,—β3,—β4, PLC-γ1, PLC-δ1 and PLC-δ2 [112, 113]. These enzymes appear to be activated by different mechanisms. It is likely that PLC-β isoforms, activated by carbachol, are localized on the PM and are regulated by the Gα_q_ subfamily of G-proteins [114]. On other hand PLC*δ*1 is localized in the cytoplasm and regulated by nutrients including glucose [115]. PLC-γ1 is entirely cytoplasmic in MIN6 cells [116]. Interestingly, PLC-γ is directly phosphorylated and activated by tyrosine kinase [114]. Therefore we modeled the two main PLC forms in β-cells as a nutrient (glucose) activated cytoplasmic PLC form (PLC_C_) and as a G-protein-coupled PLC that is activated by specific G_q_-type G-proteins in the PM (PLC_P_) (Fig 1).

PLC- β and Gα_q_ can form a complex mediating PLC activity [114]. We therefore suggested that the PLC_P_ can be activated only when this G-protein binds with its corresponding PLC (see CM). Interestingly, all the PLC isoforms discussed here can be activated by Ca^2+^ [110, 111]. However, the cytoplasmic PLC isoform activated by glucose is more strongly controlled by Ca^2+^ availability than the isoform that is activated by agonists [115, 117]. We thus considered the constant for [Ca^2+^]_c_ activation for PLC_p_ to be higher (0.4 μM) in comparison with the PLC_C_ (0.2 μM) (see CM).

#### IP_3_ and DAG handling

The dynamic intracellular concentrations of IP_3_ and DAG are determined by the rates of synthesis and degradation. We used our simple model [33] for modeling IP_3_ synthesis and degradation. The DAG model was developed similarly to the IP_3_ model (see CM). However, DAG remains bound within the PM and it was simulated as a concentration closely associated with the PM.

#### Regulation of PKC activation

DAG promotes membrane recruitment and activation of protein kinase C (PKC), an enzyme linked to the regulation of many cellular processes [118]. Several PKC isoenzymes are expressed in pancreatic β-cells and it is known that PKC phosphorylates and activates the components of the exocytotic machinery in β-cell that is important for the regulation of insulin secretion. However, the precise role of PKC-family proteins in β-cell physiology is still controversial and the phosphorylation targets that mediate the enhancement of exocytosis by PKC are not well known [119]. For this reason in our quantitative model of messenger interactions we consider only activation of P4P synthesis from PI by activated PKC (see Fig 1).

DAG can also activate PDK1 in mouse islets [120] and this kinase is also responsible for stimulating insulin release [121]. However, activated PDK1 does not seem to directly regulate the processes that we consider here and its regulation is not further considered.

#### Regulation of Ca^2+^ dynamics

Calcium is a ubiquitous second messenger that regulates many biological processes, such as cell signaling and transport, membrane excitability and substance secretion. In the β-cell Ca^2+^ homeostasis is controlled by transmembrane ion flux and involves coordinated interplay between multiple ion transport mechanisms and organelles. Ca^2+^ enters the β-cells primarily through voltage-gated Ca^2+^ channels. Additionally, Ca^2+^ store-operated current (SOC) increases [Ca^2^]_c_ (see CM). The plasma membrane of β-cells contains P-type ATPases that pump Ca^2+^ out of the cytosol to the extracellular environment [122, 123].

Ca^2+^ enters the endoplasmic reticulum (ER) via P-type ATPases (SERCA pumps, primarily SERCA2 and 3 in β-cells) using ATP and leaves predominantly through intracellular ER Ca^2+^ channels. The type III inositol 1,4,5 triphosphate receptor (IP_3_R) is the main calcium release channel in the β-cell ER. When IP_3_ binds to IP_3_R, Ca^2+^ passes from the lumen of ER into the cytosol [3, 122–124]. An additional regulatory control of IP_3_R is possible through phosphorylation of IP_3_R or through the associated proteins that bind to the regulatory domain. PKA activation leads to an opening of IP_3_R [125–127] (Fig 1). We have included models of IP_3_R in our general model (see CM).

### Developing and simulation an integrated computational system of β-cell GPCR signal transduction and metabolic signaling

We were able to construct an integrated kinetic mathematical model of second messenger pathways. The model is described by mass action kinetics and is formulated as a system of ordinary differential equations (see CM). To handle the model equations from a numerical viewpoint, we need to know the dimensions and ranges of both variables and parameters so as to confine output values within physiological limits. However, precise determination of the model coefficients is limited due to a lack of adequate experimental data. Therefore, the model parameters were taken from the literature when possible and evaluated manually to best reproduce the experimental results that were found in available literature. All parameters and constants were fitted to be in their physiological ranges.

Equations, parameters, coefficients and outputs are represented below in the “Computational Model” and contain all the information necessary to carry out the simulations presented in this paper. They are pointed out in the text as a simulation at basal levels, and all simulations used the same set of parameters except where noted in the text or in the corresponding figure legends. Our simulations give time-dependent changes of cellular parameters. This allows a comparison with the corresponding experimental time-dependent data. To calculate persistent cellular parameters, the model was allowed to run up to steady-state values without changes in coefficients.

The model consists of a system of nonlinear ordinary differential equations describing the time rate of change in parameters. We developed the mathematical submodels of regulation process in the β-cell using web-based modeling resources of “The National Resource for Cell Analysis and Modeling (NRCAM)” This system was solved using the Virtual Cell simulation framework (University of Connecticut Health Center). The entire model and simulated results is publicly available for direct simulation on the website ‘‘Virtual Cell” (www.nrcam.uchc.edu) in ‘‘Math-Model Database” on the ‘‘math workspace” in the library ‘‘Fridlyand” with name ‘‘Messengers interaction”. Free registration and corresponding instructions are available on that website. It is easy to copy, change and simulate this model with other parameters directly on the Virtual Cell platform on their website. Visualization and graphical analysis were performed using “Excel”.

## Results and Discussion

In this section we use computational simulations to critically review and analyze the experimental observations of intracellular regulatory mechanisms. Increases in the intracellular concentrations of Ca^2+^, cAMP and DAG contribute to increased insulin secretion [3, 93, 123]. This allows us to compare data on insulin secretion with simulated results of second messenger pathway activation dynamics.

### Simulation of glucose action (Fig 3, left part)

At low glucose concentrations (defined here as 3 mM) the simulated resting PM potential is approximately -62 mV and [Ca^2+^]_c_ is approximately 0.1 μM, corresponding to experimental data [122, 128]. The constitutive concentrations of activators for each specific G-protein coupled receptors were chosen to obtain 1/10 activity of the corresponding PM bound enzymes.

**Fig 3:**
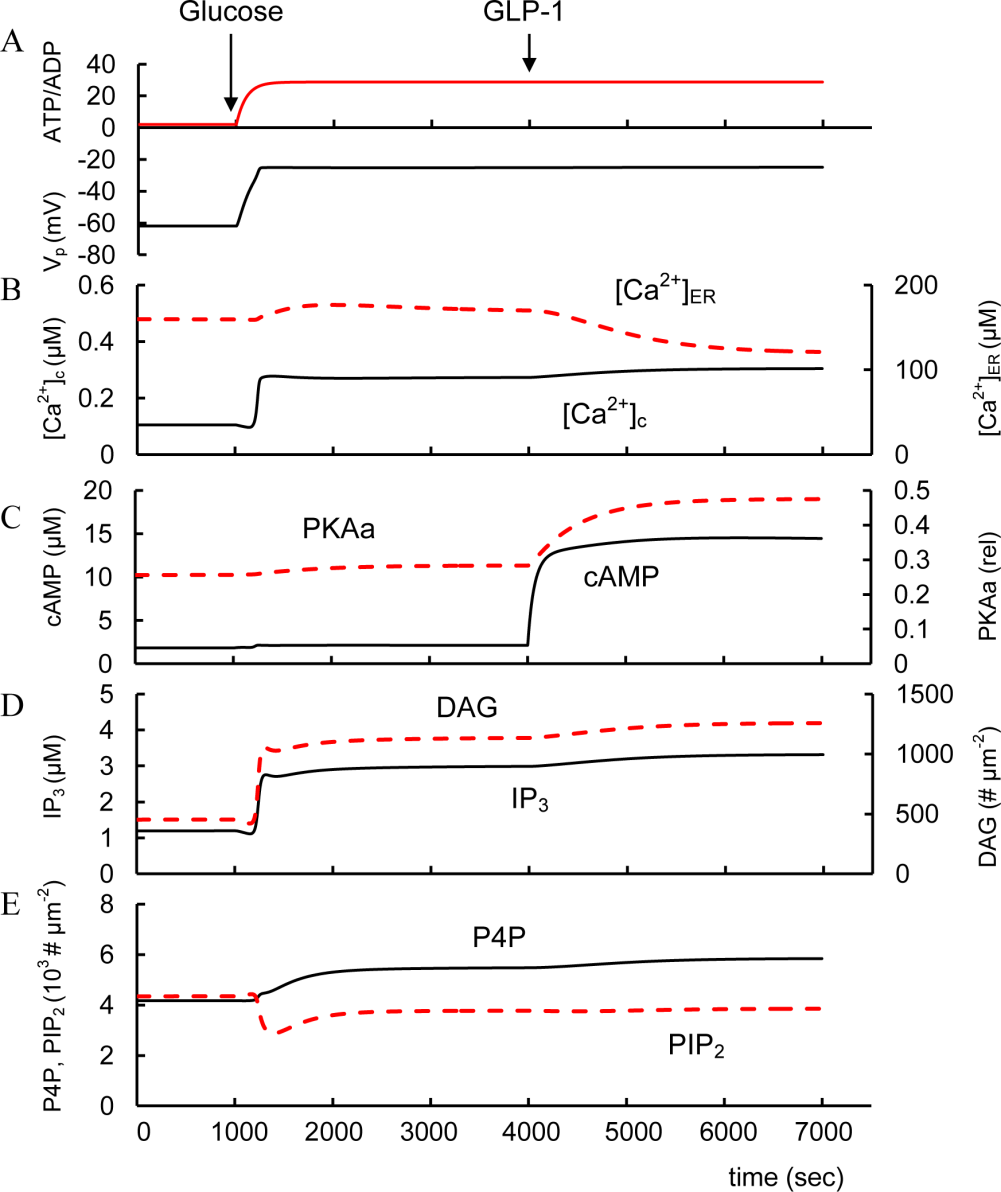
Modeling of spontaneous glucose-and GLP-1 stimulated changes of intracellular parameters. (A) ATP/ADP and PM potentials (V_p_); (B) Free cytoplasmic Ca^2+^ concentration ([Ca^2+^]_c_) and free Ca^2+^ concentration in ER ([Ca^2+^]_ER_); (C) Cytoplasmic cAMP and relative activated PKA (PKAa). (D) IP_3_ and DAG concentrations. (E) Concentrations of P4P and PIP_2_ on PM. Initially all coefficients were on basal level (see Tables in CM). Low glucose concentration (3 mM) was simulated initially (left part). An increased extracellular glucose level at 1000 sec (from 3 to 8 mM) stimulates ATP/ADP increase that blocks K_ATP_ channels. This inhibition allows the PM depolarization (Fig 3A) that activates the voltage-gated Ca^2+^ channels and increases cytoplasmic Ca^2+^. This activate SERCA and increase Ca^2+^ in the endoplasmic reticulum ([Ca^2+^]_ER_). Increased [Ca^2+^]_c_ activates also PLC and correspondently increases IP_3_ and DAG, decreases PIP_2_ and increases P4P concentrations. Increase in GLP-1 (from 3.1e-7 μM to 6.2e-4 μM) with corresponding AC_P_ activation was simulated at 4000 sec. This leads to a fast increase in cAMP concentration and PKA activation (Fig 3C). Rise in PKA activity increases Ca^2+^ discharge from the ER through the inositol 1,4,5 triphosphate receptor (IP_3_R) and decreases [Ca^2+^]_ER_ (Fig 3B).

A simulation of increasing extracellular glucose would produce a rise in the ATP/ADP ratio that blocks K_ATP_ channels. This inhibition would allow the PM depolarization that activates the voltage-gated Ca^2+^ channels and increases cytoplasmic Ca^2+^ (Fig 3). Increased [Ca^2+^]_c_ activates constitutive PM bound and G-protein independent PLC activity and thereby increases IP_3_ and DAG concentrations. DAG activates PKC. PKC and Ca^2+^ also activate phosphatidylinositol 4-kinases leading to an increase in P4P concentration and corresponding acceleration of IP_3_ and DAG production. cAMP dynamics is considered later.

Increased [Ca^2+^]_c_ accelerates Ca^2+^ entry into the ER by SERCA pumps that can increase [Ca^2+^]_ER_. Simulated increase in [Ca^2+^]_ER_ (Fig 3B) corresponds to the experimental data that have shown an increased [Ca^2+^]_ER_ with increased glucose [129–131]. The half-time for increased [Ca^2+^]_ER_ after increased glucose was about 10 min [129] matched by our simulations (Fig 3B).

**Fig 4:**
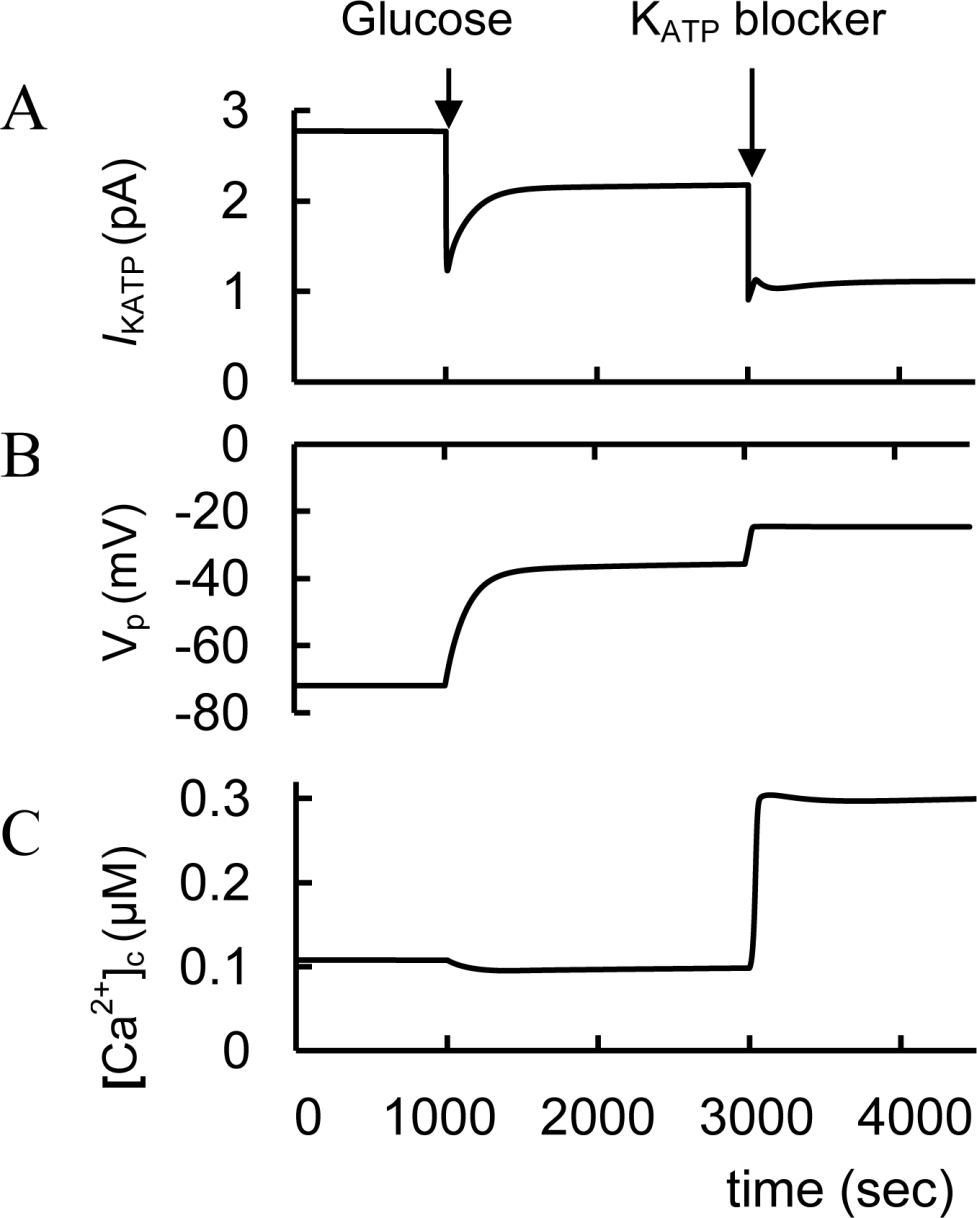
Simulated response to block of K_ATP_ channel in type 2 diabetes (T2D). (A) K_ATP_ channel current (*I*_KATP_), (B) PM potential (V_p_); (C) [Ca^2+^]_c_. T2D conditions were simulated by a decrease of the coefficient that is responsible for glucose dependent saturated ATP/ADP ratio (ATD_m_, Eq 4). ATD_m_ was decreased from 32 (basal level) to 15. In this case PM membrane potential does not increase up to the nessesary threshold and does not lead to [Ca^2+^]_c_ increase as glucose is increased from 3 mM to 8 mM at 1000 sec. For simulation of K_ATP_ channel blocking the maximal conductance (g_mKATP_, Eq 9) was decreased from 24 nS (basal level) to 9 nS at 3000 sec. This induces additional PM depolarization and fast [Ca^2+^]_c_ increase.

In at least some islets from patients with T2D, althought data is extrememly limited, there seems to be a limited glucose-stimulated ATP production in β-cells so that glucose is not fully capable of closing K_ATP_ channels in order to stimulate Ca^2+^ influx [132–134]. Under these pathophysiological conditions, glucose alone fails to generate the critically important cytosolic Ca^2 +^ signal that initiates insulin exocytosis. By facilitating glucose-dependent K_ATP_ channel closure, sulfonylureas can restore the Ca^2 +^ signal, thereby allowing glucose-stimulated insulin secretion (GSIS) to occur [135]. We were able to simulate this mechanism. For example, we simulated the decrease of the glucose dependent saturated ATP/ADP ratio that leads to decreased PM potential and a failure to increase [Ca^2 +^]_c_ in GSIS. Additional closure of K_ATP_ channels can depolarize the PM and restore increased [Ca^2+^]_c_ in GSIS (Fig 4).

### cAMP pathway regulation

Cellular cAMP level is determined by the relative activities of AC and phosphodiesterases (PDEs). The association of cAMP effectors with signaling complexes and PM bound AC (AC_p_) that regulate cAMP concentration (Fig 2) explains the differential signaling initiated by members of the G_s_-and G_i_-protein receptor families.

Glucose stimulation alone has led to either unchanged [136] or insignificantly increased cAMP levels [137–139]. Our simulation (Fig 3C) explains the insignificant increase in cAMP levels due to low activity of G-protein dependent PM bound AC (AC_P_) when the specific hormone is absent, closely reflecting the corresponding experimental data. For example, according to [78] cAMP concentration in rat β-cells corresponds to 2.6 fmol 10^3^ cells^-1^ at low glucose levels (1.4 mM) and 3.6 fmol 10^3^ cells^-1^ at high glucose (20 mM). We converted these units in 1.81 μM and 2.51 μM for cAMP concentration in cell using volume of single β-cell. Our simulated cAMP concentrations were 1.83 μM at low and 2.104 μM at high glucose similar to the experimental data (Fig 3C). In this case cAMP dynamics are determined by constitutively active G-proteins that activate AC_P_ and soluble AC (AC_c_) (both can be activated by Ca^2+^/CaM) and Ca^2+^-dependent PDE isoforms. The insignificant increase in cAMP concentration may result from offsetting events: calcium-stimulated phosphodiesterase activity may offset increased AC activity.

Even a low cAMP level may be necessary for insulin secretion because decreasing cAMP inhibits GSIS. For example phosphodiesterase 3β **(**PDE3B) negatively regulates insulin secretion during GSIS through its cAMP-hydrolyzing activity [140].

#### Incretin hormones

GIP is secreted in the same time and concentration frame as GLP-1 and these concentrations are sufficient to activate GLP-1R or GIPR in humans [141, 142]. GLP-1 and GIP receptor activation on β-cells leads to increased cAMP and intracellular Ca^2+^ that result in exocytosis of insulin-granules as a late response to oral glucose (20–120 min) [143]. GLP-1 and GIP have insulinotropic action in healthy humans [141, 144, 145].

We modeled the actions of incretin hormones and the resulting activation of AC_P_ that increases cAMP synthesis and PKA activation. To do this we set up a simulation with GLP-1 and GIP levels 20 times higher than the Michaelis constant for GLP-1 or GIP receptor binding (coefficient K_GLP1_, or K_GIP_, Eqs 46 and 47). As illustrated in Figs 1 and 3 simulation of increased cAMP leads to dual activation of the PKA and Epac branches of the cAMP pathway. Indeed it was found that GLP-1 and GIP lead to additional glucose-dependent closure of K_ATP_ channels and an increase in Ca^2+^ release from ER leading to [Ca^2+^]_c_ increase, that accelerates Ca^2+^ dependent insulin secretion [64, 68, 93, 146]. We were able to simulate increased [Ca^2+^]_c_ (and a corresponding fall in [Ca^2+^]_ER_) by GLP-1R activation as a consequence of IP_3_R stimulation through PKA activation (Fig 3).

**Fig 5:**
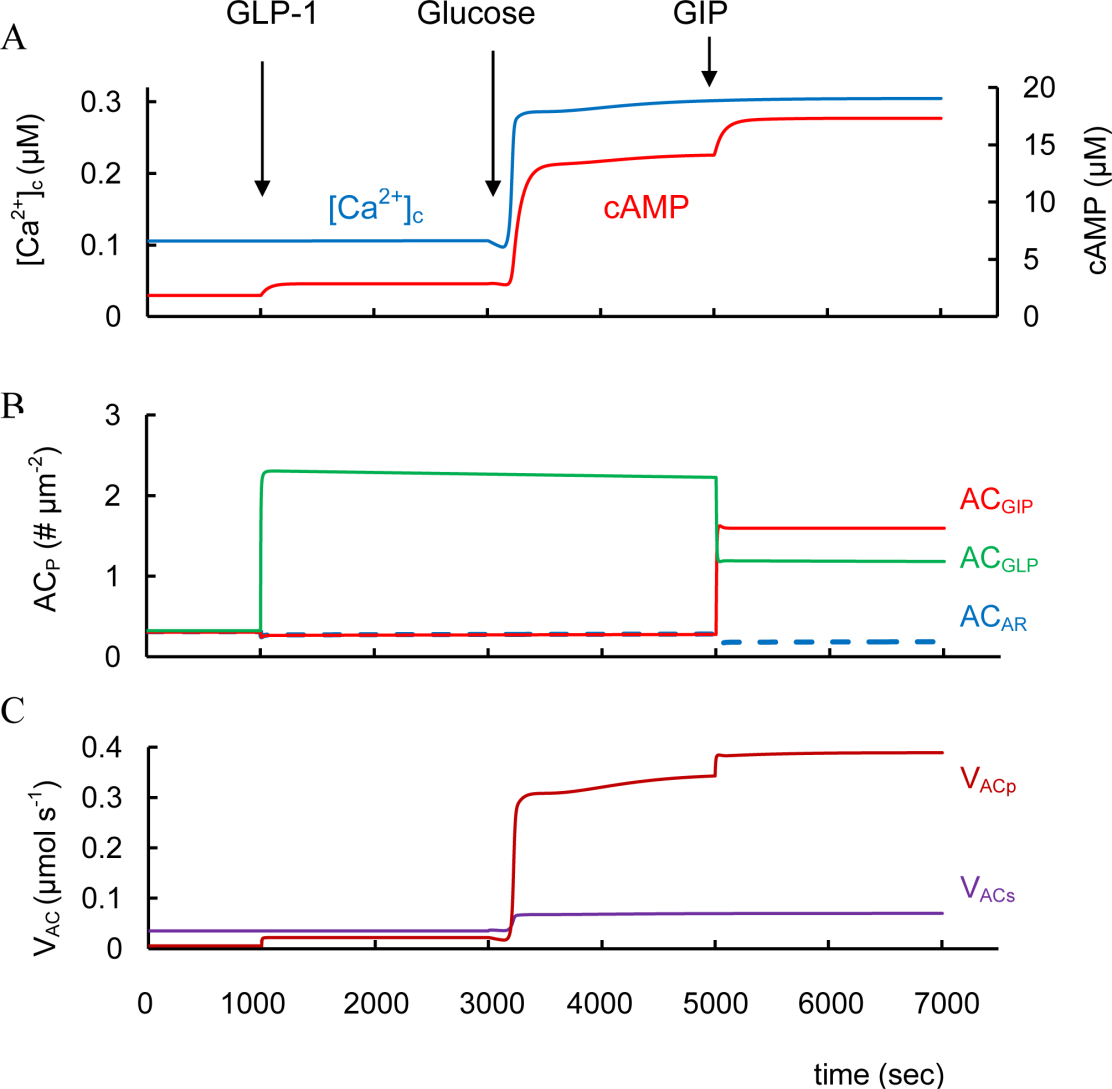
Simulation of GLP-1 and GIP action at low and high glucose levels. (A) Cytoplasmic [Ca^2+^]_c_ and cAMP dynamics. (B) AC_GLP_, AC_GIP_ and AC_AR_ are the concentrations of complexes of AC on PM (AC_P_) bound with corresponding G-proteins activated by GLP-1R, GIPR and α_2A_ adrenergic receptors. (C) V_ACp_ is G-protein and Ca^2+^ dependent AC activity on PM, V_ACc_ is the glucose and Ca^2+^-activated soluble AC activity that is independent on G-protein. At low glucose concentrations (3 mM) (left part) GLP-1 was increased from basal level (3.1e-7 μM) to 6.2e-4 μM at 1000 sec. Increased extracellular glucose level was simulated at 3000 sec (from 3 mM to 8 mM). Than GIP administration was modeled as the increased GIP from basal level (1.37e-6 μM) to 3.42e-3 μM at 5000 sec.

However, in low glucose concentrations treatment of pancreatic β-cells with agents that increase cAMP- alone has little or no effect on insulin secretion. Only a combination of GLP-1 or GIP and high glucose potentiates GSIS [84, 147]. This important feature reduces the chance of producing hypoglycemia in patients with T2D with GLP-1 agonists [148]. Our computational model agrees with this behavior (see Fig 5, left part). At low glucose when [Ca^2+^]_c_ is low, activation of GLP-1R significantly increases AC_P_ (Fig 5B), however this does not lead to increased cAMP because increased [Ca^2+^]_c_ is required for ACp activation. On the other hand the model predicts that a facilitation of cAMP production by AC_P_ by GLP-1R agonists can be stimulated if glucose increases the concentration of Ca^2+^-dependent calmodulin that can activate PM bound AC in β-cells (see Figs 3 and 5). A small effect of GLP-1 on increased cAMP at low glucose was also simulated in our previous model of AC activation and of cAMP dynamics [23] becouse this effect can be explained by the sensitivity of AC_P_ to [Ca^2+^]_c_ changes rather than by the properties of GLP-1R. (A similar simulation of GLP-1 effects via AC activation was reported [24]). This and our previous analysis [23] provides support for a pivotal role of Ca^2+^/CaM-dependent AC activation (possibly the AC8 isoform) in GLP-1 effects. The potentiating effect of cAMP on insulin secretion requires synergism between the cAMP pathway and the Ca^2+^ signal. Our model of cAMP dynamic regulation may also help to explain the mechanism responsible for the pattern of cAMP changes that take place during phases changes and oscillations in intracellular Ca^2+^ [23].

This model also provides an explanation for the observed clinical interaction between GLP-1R agonists and K_ATP_ channel blockers. Several human clinical trials have demonstrated that patients using GLP-1R agonists and K_ATP_ channel blockers therapies have a greater incidence of hypoglycaemia than those not using K_ATP_ channel blockers [148]. K_ATP_ channel blockers have also been shown to abrogate the glucose dependence of GLP-1R agonist activity in the rat pancreas [149].

Although the mechanism of this proposed uncoupling event has not been fully elucidated experimentally, our simulation suggests that K_ATP_ channel blockers may lead to modest PM depolarization and increased Ca^2+^ concentration even in low glucose (not shown) leading to activation of Ca^2+^ dependent AC on PM and an increased cAMP (and corresponding insulin secretion) in the presence of GLP-1R agonists. Thus, block of K_ATP_ channels independently of glucose may allow GLP-1R agonists to bypass their inherent glucose requirement by stimulating the downstream effects ordinarily associated with increased glucose. Our simulation shows also that similar behavior may also occur for GIPR activators in the presence of K_ATP_ channel blockers (not shown).

#### GLP-1 and GIP interaction

In rodent and healthy human subjects the insulinotropic effectiveness of GLP-1 and GIP may be additive [141]. We were able to simulate an additional increase in cAMP concentration following GIPR activation with GLP-1 administration (Fig 5B). This synergetic GIP effect takes place because GLP-1R cannot fully activate AC_p_ and GIPR administration leads to additional AC_p_ activation in our model, i.e. a sum of AC_p_ activated by GLP-1R (AC_GLP_) and GIPR (AC_GIP_) was higher than AC_GLP_ with GLP-1 activation only.

However, investigations in mice have shown a less protracted action of GIP vs GLP-1 that would suggest a more prolonged activation of the GLP-1 receptor than that of GIPR [150]. One hypothesis, is that GIP provokes accelerated desensitization of the GIPR [150]. Ligand-induced desensitization and trafficking of GPCRs have been implicated as critical mechanisms for modulating response duration *in vivo* [151]. A naturally occurring variant of the GIP receptor underwent enhanced agonist-induced desensitization in adipocytes, which impaired GIP control of adipose insulin sensitivity [152]. We have exploited our mathematical model to examine the influence of receptor desensitization in cAMP dynamics. According to results of our modeling an increase in the rate constant for desensitization and/or a decrease of rate of return to surface for GIPR can actually decrease AC_p_ activity bound with GIPR to compare with AC bound with GLP-1R (not shown). However, we were not able to find any data where a signaling desensitization in response to these two hormones has been compared under the same experimental setting in pancreatic β-cells.

#### Incretin hormones and T2D

**Fig 6:**
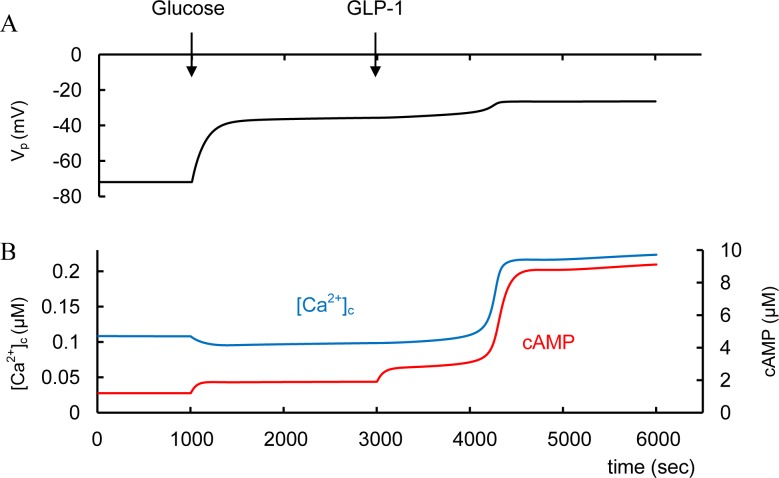
Simulated response of GLP-1 action in T2D conditions. T2D conditions were simulated by decreasing the glucose dependent saturated ATP/ADP ratio (ATD_m_, Eq 4). ATD_m_ was decreased from 32 (basal level) to 15 as in Fig 4. In this case PM membrane potential is decreased and does not lead to [Ca^2+^]_c_ increase as glucose increases from 3 mM to 8 mM at 1000 sec as well as in Fig 4. GLP-1 administration was simulated at 3000 sec ([GLP-1] was increased from 3.1e-7 μM to 6.2e-4 μM. This induces cAMP increase with additional PM depolarization and Ca^2+^ influx into cell. Corresponding PKA activation also increases Ca^2+^ flow from ER. These effects lead to significant [Ca^2+^]_c_ and cAMP increase.

GLP-1 may be able restore a Ca^2+^ signal by facilitating glucose-dependent K_ATP_ channel closure and by enhancing SOC allowing normal GSIS [64]. We were able to simulate this mechanism (Fig 6). In Fig 4 we suggested that in T2D glucose leads to an insufficiently increased ATP/ADP ratio (as a consequence of some unspecified dysfunction). We simulate the initial T2D conditions in Fig 6 (as well as in Fig 4) as decreased glucose dependent saturated ATP/ADP ratio that leads to a inability to increase [Ca^2+^]_c_ during GSIS. However, GLP-1 administration then leads to additional depolarization and [Ca^2+^]_c_ increase in our model (Fig 6). This additional depolarization is a consequence of increased *I*_SOC_ due to decreased [Ca^2+^]_ER_ after IP_3_R activation by PKAa, that also leads to increased Ca^2+^ flux from ER to cytosol. PKA activation also decreases the conductance of K_ATP_.

Patients with T2D can have preserved GLP-1 and GIP secretion [142, 153]. However, the insulinotropic effect of incretins is diminished in T2D patients, due in part to reduced expression of incretin receptors as a consequence of perhaps glucotoxicity or lipotoxicity. For example, GLP-1R and GIPR levels were decreased in islets from mouse and rat model of diabetes and from T2D patients [57, 154, 155]. Elevated non-esterified fatty acids lead to a decreased GLP-1R expression and downregulation of GLP-1 receptor signaling in β-cell lines and mouse islets from db/db mice [156]. On other hand the transgenic expression of GLP-1R was able to restore GLP-1R-dependent stimulation of cAMP in isolated islets from mouse and in insulinoma cell lines [156, 157]. It was also shown that at high glucose levels GLP1-R can also be covalently modified in mouse islets by small ubiquitin-related modifier protein (SUMO) reducing its ability to be stimulated by GLP-1R agonist [56]. We have exploited our model to analyze these results. Proportional loss of cell surface GLP-1R and GIPR leads to a decreased cAMP and Ca^2+^ level even in the presence of high GLP-1 and GIP stimulation because the total receptor number is a limiting factor for activation of G-protein bound AC in our model. For example, a decrease in the total GLP-1R by 2-fold leads to a decrease of cAMP from 10.5 to 4.5 μM in a simulation of GLP-1 administration (if the calculations were made similar to Fig 3).

In summary, the results in this section suggest that the concentration of incretin hormone receptors on the PM can be a limiting factor in incretin function in rodent, normal human and T2D patients.

#### Catecholamines

**Fig 7:**
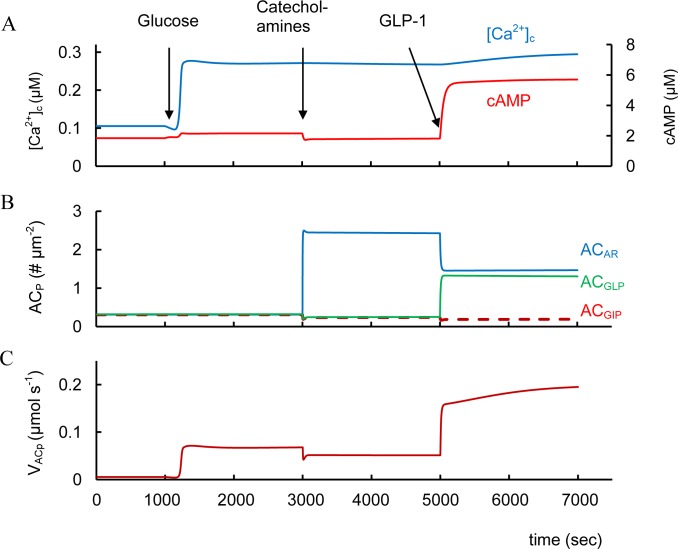
Simulation of catecholamines and GLP-1 interaction at high glucose levels. (A) Cytoplasmic cAMP and [Ca^2+^]_c_ dynamics. (B) AC_GLP_, AC_GIP_ and AC_AR_ are the concentrations of complexes of AC on PM (AC_P_) bound with corresponding G-proteins activated by GLP-1R, GIPR and α_2A_ adrenergic receptors. (C) V_ACp_ is G-protein and Ca^2+^ dependent AC activity on PM. The initial simulation was with low glucose (3 mM) as in Fig 3. Increase of glucose (8 mM at 1000 sec) induced changes of intracellular parameters. Catecholamine concentration (AR_3_) was increased from basal level (0.002 μM) to 3 μM at 3000 sec. Than GLP-1 was increased from basal level (3.1e-7 μM) to 6.2e-4 μM at 5000 sec.

Catecholamines (epinephrine and norepinephrine), the agonists of alpha adrenergic receptors, inhibit insulin secretion by decreasing cAMP content in β-cell as a result of inhibition of AC activity [10]. Our simulation also shows a catecholamine inhibition of cAMP concentration that can reduce GSIS (Fig 7A). This is a consequence of block of PM bound AC (AC_P_) activity because this AC isoform following catecholamine-receptor binding cannot produce cAMP (see Fig 7B). AC_P_ plays a role in maintaining low levels of cAMP at GSIS in our model even without GPCR agonists activating the cAMP pathway. This is a consequence of constitutive activity of GPCRs that activate AC_p_. Even low cAMP levels may be necessary for insulin secretion (see above). Therefore, activation of catecholamine receptors decreasing AC_p_ activity further suppresses GSIS even though cAMP content may already be low.

Subthreshold *α*_2_-adrenergic activation with clonidine counteracts GLP-1 potentiation of GSIS [158]. Our simulation also shows that activated adrenergic receptors can block an increased cAMP concentration by incretin hormones because a significant part of PM bound AC is bound with G_i_-proteins activated by catecholamine receptors. It blocks the ability of AC_P_ to be activated by G α_s_-protein-coupled receptors. In this case the increase in cAMP concentration after simulation of GLP-1 was reduced (~6 μM, Fig 7A) in comparison with simulation in the absence of *α*_2_-adrenergic activation (~15 μM, Fig 3C).

α_2A_ receptor antagonists have been proposed as T2D therapeutic agents [159]. We used our model to evaluate this possibility. Our simulation showed that a decrease in the number of α_2A_ receptors does not lead to a significantly increased cAMP concentration during GSIS, when we assume that α_2A_ receptor number is small (to reflect basal levels of this receptor accepted in our model). For example, with glucose stimulation (as in Fig 3) the increase in cAMP concentration is insignificant (from 2.104 to 2.106 μM) if the α_2A_ receptor content (Re_ARt_ in Eq 48) is lowered 10-fold. This happens because constitutive catecholamine activity blocks only a small fraction of the PM bound AC in the absence of specific activators (see Fig 5B). Block of this fraction cannot significantly effect activation of the remaining fraction of AC_P_ by incretins (not shown). Consequently, α_2A_ receptor antagonists are unlikely to be useful in T2D if the activity of α_2A_ receptors is insignificant.

However, T2D diabetes risk can be associated with increased expression of α_2A_ receptors and a concomitant reduction in insulin secretion [160]. Our simulation of cAMP dynamics with an increased α_2A_ receptor expression level (even without their agonists being present) gives a result that is indeed similar to *α*_2_-adrenergic activation shown in Fig 7, i.e. to significant decrease in cAMP. This shows that increased α_2A_ receptor expression should lead to decreased cAMP production during GSIS without and with the incretin hormones (under physiological conditions). AC_P_ activity is reduced since a significant part of PM bound AC may be inhibited (bound with G_i_-proteins) and cannot be activated during GSIS or by incretin receptors. In this case α_2A_ receptor antagonists might be useful in increase insulin secretion. It does seem that this effect is limited to situations where this is significantly increased expression of α_2A_ receptors over control situations.

#### Regulation of AC and PDE activity

Regulation of intracellular cAMP concentration during GSIS is also possible using AC activators such as forskolin or a PDE inhibitor (e.g., IBMX) [139]. For example, β-cell PDE3B can be activated by insulin, IGF-1 and leptin that inhibit insulin secretion [140]. Interestingly, PDE4 inhibitors may increase insulin secretion in humans [161].

Increased cAMP by AC activation or PDE inhibition causes various events, including Ca^2+^ mobilization from internal Ca^2+^ stores and activation of non-selective cation channels [80, 84]. In our model, activation of AC or PDE inhibition increased cAMP concentration, similar to the above for GLP-1 or GIP stimulation (see below for a special case of expression of different PDE isoforms). Then increased cAMP leads to PKA activation and block of K_ATP_ channels, activation of Ca^2+^ release from ER and additional PM depolarization (see Figs 1 and 6). Notice that dual activation of the PKA and Epac branches of the cAMP signaling mechanism can act directly on the exocytotic machinery [68, 146].

### PLC pathway regulation

PLC activation increases insulin secretion in mouse and rat islets [3, 100, 115]. Drug-mediated, chronic, and selective activation of β-cell G_q_ signaling greatly improves β-cell function and glucose homeostasis in mice [162].

Constitutuve PLC activity occurs at low glucose levels. Increased glucose can activate the PLC pathway and significantly increase IP_3_ and DAG concentration in conditions when PLC pathway GPCRs are not activated [95, 124, 163]. We simulated this process (Fig 3D) and found that the increases in IP_3_ and DAG following glucose stimulation occur through cytoplasmic Ca^2+^ activation of both G-protein dependent and independent forms of PLC. In this case the degradation rate for IP_3_ or DAG does not increase with increased [Ca^2+^]_c_. This is in contrast to cAMP degradation where Ca^2+^ activates phosphodiesterases. Additionally, PLC activation during GSIS and the corresponding decrease in PIP_2_ concentration can simulate K_ATP_ channels closure and increased Ca^2+^ influx through voltage-activated Ca^2+^ channels (see Figs 1 and 3E).

#### FFAR1/GPR40 receptops

FFAR1/GPR40 agonists enhance insulin secretion. They markedly enhance cytoplasmic and mitochondrial Ca^2+^ in insulinoma cells and increased IP_3_ and ATP levels in islets normal and diabetic rats [164]. GSIS (i.e. at high glucose level) was also augmented in pancreatic β-cells in normal and diabetic mice that overexpressed FFAR1/GPR40 [165, 166].

**Fig 8:**
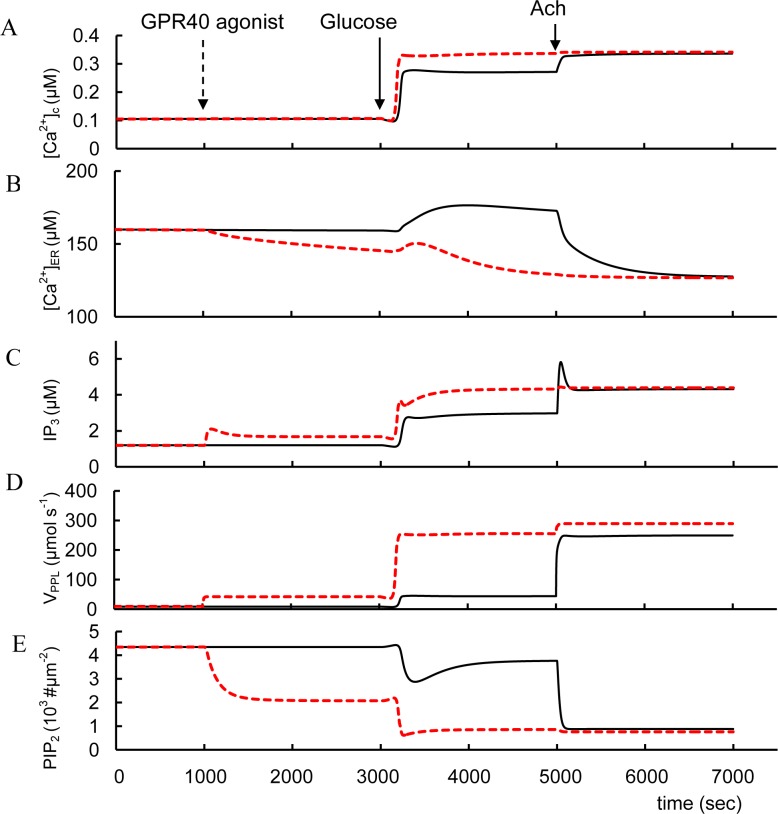
Effects of FFAR1/GPR40 activators and acetylcholine (Ach) on changes of intracellular parameters. Two protocols were stimulated: 1. (---) glucose was increase from 3 mM to 8 mM at 3000 sec, than acetylcholine, M_3_R activator, (AM_3_ concentration in model) was added at 5000 sec (AM_3_ was increased from basal level 0.0022 μM to 2.2 μM) and 2. (- - - -) FFAR1/GPR40 agonist was added at 1000 sec (AR_7_ was increased from basal level 0.0506 μM to 82 μM), than glucose (3000 sec) and acetylcholine were added (5000 sec). (A) Free cytoplasmic Ca^2+^ concentration ([Ca^2+^]_c_. (B) Free Ca^2+^ concentration in ER ([Ca^2+^]_ER_); (C) IP_3_ concentration. (D) V_PLP_ is the activity of PM bound PLC (Eq 78). (E) Concentrations of PIP_2_ on PM.

We simulated the effect of FFAR1/GPR40 activation via Gα_q_-proteins leading to PLC (PLC_p_) pathway activation (Fig 8). However, this effect was insignificant for Ca^2+^ and IP_3_ at low glucose levels and only increased glucose levels (that can increase [Ca^2+^]_c_) can significantly activate PLC_p_ (Fig 8D). This leads to an additional rise in IP_3_ and DAG concentrations. Increased IP_3_ also results in decreased [Ca^2+^]_ER_ and an increase in [Ca^2+^]_c_ as a result of IP_3_-dependent release of Ca^2+^ from the ER. Decreased [Ca^2+^]_ER_ also opens store-operated channels (SOC) accelerating Ca^2+^ and possibly other cations into the cytoplasm through these channels. PLC_p_ activation decreases PIP_2_ that can also lead to additional K_ATP_ channels inhibition (see Fig 8E). All these processes enhance PM depolarization, increasing Ca^2+^ influx (Fig 8A). Formation of DAG also activates PKC, resulting in increased efficiency of Ca^2+^ on exocytosis during GSIS (Fig 1).

These simulations reflect experimental data. Unbound FFAs in plasma in the physiological range can activate FFAR1/GPR40 in the presence of glucose and eventually amplifies GSIS in short-term exposure [106, 167]. FFA action on FFAR1/GPR40 caused PLC activation and a rise in [Ca^2+^]_c_ both in primary mouse and rat β-cells and in INS-1 cells with increased glucose [168–170]. Palmitate also markedly reduced the calcium storage capacity of the ER measured by thapsigargin-induced Ca^2+^ release, suggesting that ER calcium stores may participate in raising [Ca^2+^]_c_ in isolated mouse islets [37]. These events are consistent with a decrease in [Ca^2+^]_ER_ following FFAR1/GPR40 activation in our simulation (Fig 8B).

We evaluated the effect of FFAR1/GPR40 potentiation at low glucose in more detail to evaluate possible enhanced risks of hyperglycemia. At low glucose concentrations, activating FFAR1/GPR40 does not increase [Ca^2+^]_c_ in pancreatic rat β-cells [170] nor does it stimulate insulin secretion in mouse islets [166]. Interestingly, mouse pancreatic β-cells expressing increased FFAR1/GPR40 do not show increased insulin secretion in low glucose [165]. PLC activity is unaffected in our model (Fig 8D left part), because increased cytosolic Ca^2+^ is required to increase G-protein dependent PLC activity. This is an advantageous feature that should reduce the chance of producing hyperinsulinemia and hypoglycemia in T2D patients given FFAR1/GPR40 agonists. Indeed, selective pharmacological activation of FFAR1/GPR40 by TAK-875 significantly improved glycemic control in T2D patients with only a modest risk of hypoglycemia [171]. This result resembles our analysis of GSIS stimulated by GLP-1, where GLP-1 action alone (i.e. at low glucose) has little or no effect on insulin secretion. Only a combination of GLP-1 and high glucose potentiates insulin secretion. We explained this effect also through the dependence of increased [Ca^2+^]_c_ to increase G-protein dependent AC activity.

We can also predict that using FFAR1/GPR40 agonists simultaneously with K_ATP_ channel blockers can lead to increased incidence of hypoglycemia similar to employing GLP-1R agonists and K_ATP_ channel blockers together. Both of these effects are associated with an increase in [Ca^2+^]_c_ due to sulfonylureas or similar K_ATP_ inhbiting drugs that can activate PLC or AC even in low glucose levels. Indeed, FFAR1/GPR40 activation increased insulin secretion in the presence of sulfonylurea under low glucose conditions through enhancement of PKC signaling in insulinoma cells [172].

Activation of FFAR1/GPR40 may be a viable therapeutic approach for treatment of type 2 diabetes [173]. Our simulations suggest that FFAR1/GPR40 should increase insulin secretion in the presence of elevated glucose, because FFAR1/GPR40 agonists should increase [Ca^2+^]_c_ and DAG during GSIS, supporting the evidence that these agonists may be useful as novel insulin secretagogues with low risk of hypoglycemia. However, the simulations also indicate that using FFAR1/GPR40 activators together with sulfonylureas could increase the incidence of insulin secretion overshoot and thereby increase hypoglycemia, as it was discussed for incretin hormones.

#### Activation of muscarinic receptors

Cholinergic muscarinic agonists, including the endogenous neurotransmitter acetylcholine and its synthetic nonhydrolyzable analog carbachol, increase [Ca^2+^]_c_ and GSIS in normal β-cells [3]. Stimulation of the MR activates Gα_q_-proteins leading to PLC_p_ activation in our model (Fig 1) Modeling of MR activation is shown in Fig 8 (right) for high glucose levels for two cases, with and without preliminary FFAR1/GPR40 activation. This simulation (without preliminary FFAR1/GPR40 activation) corresponds to experimental evidence that at high glucose level, stimulation with the muscarinic receptor agonists (for example, carbachol), induces a rapid and sustained PLC activation [124, 174], depolarizes PM and enhances glucose-induced electrical activity [175]. Several studies have also suggested that acetylcholine can regulate [Ca^2+^]_c_ dynamics leading to [Ca^2+^]_c_ increase and [Ca^2+^]_ER_ decrease [129, 176]. Our model also takes into account a suggestion that the depolarizing effects of the muscarinic receptor agonists can be attributed partially to a store-operated current (SOC) that activates following IP_3_-dependent release of Ca^2+^ from the ER.

β-cell specific inactivation of genes encoding Gα_q/11_ in mice leads to decreased PM depolarization with increased glucose [108]. According to our simulations this result seems to be a consequence of decreased SOC because (1) [Ca^2+^]_ER_ does not decrease significantly without IP_3_ and (2) K_ATP_ channels remain open because PIP_2_ concentration does not decrease markedly without PLC activation.

The effect of glucose and carbachol on GSIS is at least additive in rat islets [115]. Based on our model this effect can be explained by significant independence of PLC pathway that increases IP_3_ and DAG (DAG content changes proportionally IP_3_ in our model) (Fig 8C) and glucose activation of GSIS through PM depolarization and [Ca^2+^]_c_ increase (Fig 8A).

Moderate levels of acetylcholine have little effect on PM depolarization or on sustained cytosolic Ca^2+^ in mouse islets at low glucose [124, 176–178]. We simulated activation of muscarinic receptors (MR) at low glucose levels. In this case, activation of MR evoked only a small increase in [Ca^2+^]_c_ similar to modeling of FFAR1/GPR40 activation (for example, see Fig 8, left) if conductivity of specific nonselective cation channels (NALCN) (that can be activated by MR) was insignificant (not shown). This can be explained as well as for FFAR1/GPR40 activation (see Fig 8) so that increased cytosolic Ca^2+^ is necessary for activation of membrane bound PLC.

**Fig 9:**
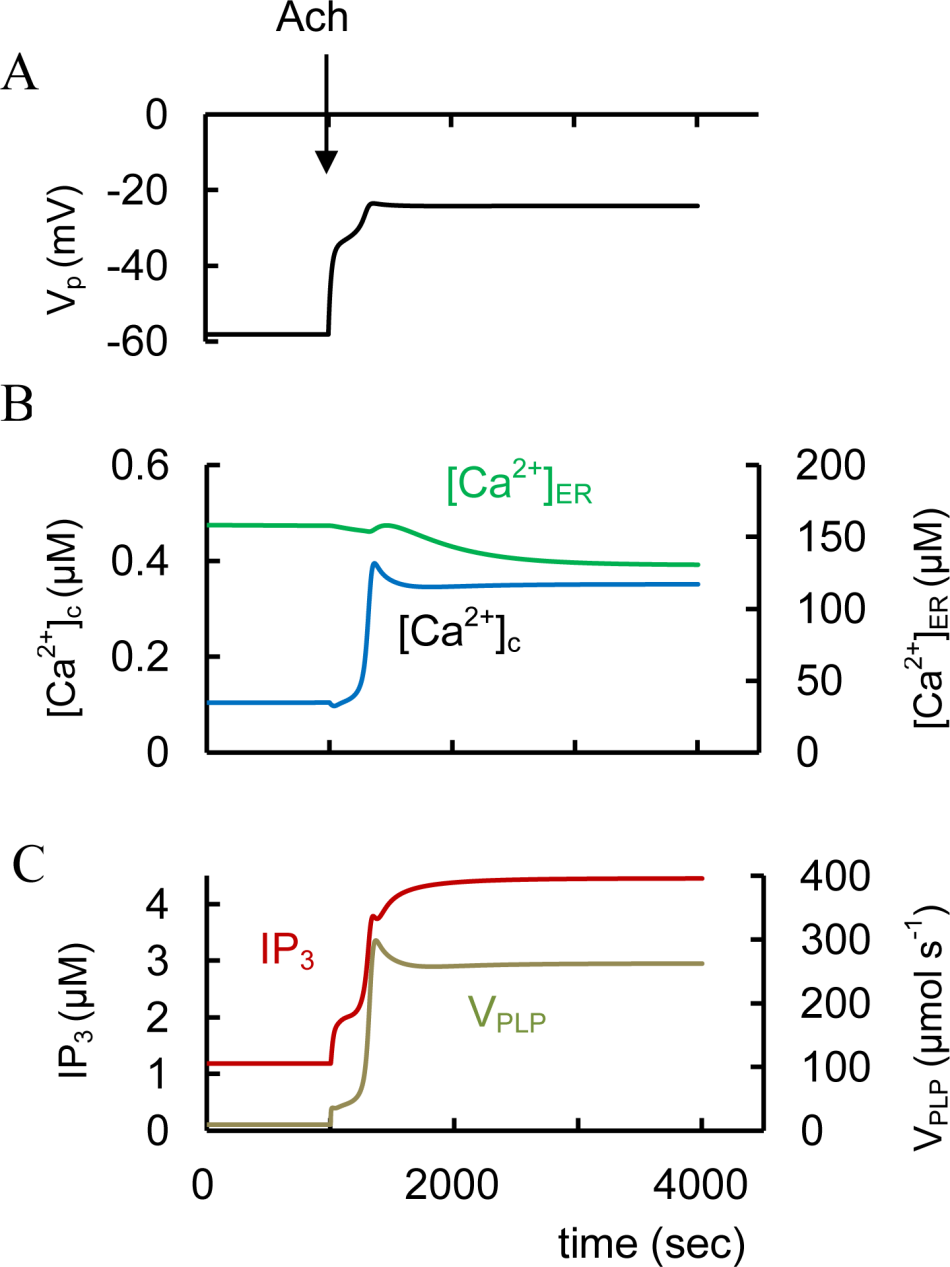
Effects of Ach on changes of intracellular parameters at activated NALCN channels at low glucose. (A) PM potential (V_p_); (B) [Ca^2+^]_c_ and [Ca^2+^]_ER_; (C) Cytoplasmic IP_3_ concentration and the activity of PM bound PLC (V_PLP_). For simulation of NALCN channel activity the maximal conductance (g_mNM3_, Eq 25, CM) was initially increased from 0 nS (basal level), to 35 nS. Addition of acetylcholine that opens NALCN channel was simulated at 1000 sec at the arrow (AM_3_ was increased from basal level 0.0022 μM to 2.2 μM). All simulations were performed at low glucose (3 mM).

A different scenario was found for rat or human islets. Acetylcholine increased [Ca^2+^]_c_ in isolated islets from Wistar rats at low glucose (2.8 mM) [179] and in human β–cells [62]. These results can be explained by activation of the MR dependent NALCN channels that lead to transient PM depolarization and increased [Ca^2+^]_c_ even with the simulation conducted in low glucose (Fig 9). Likewise, our simulation of electrophysiological events in human β-cells showed that activation of NALCN channels can lead to additional PM depolarization and increased insulin secretion at high glucose. This can also decrease the threshold for glucose-induced spike activity and also lead to increased [Ca^2+^]_c_ [30].

Acetylcholine activates Na^+^ influx by an activation of NALCN permeable to Na^+^ [3], i.e. an increased Na^+^ inward current through NALCN leading to PM depolarization (see [30, 50]) that corresponds to mechanisms considered in our model, where opening of NALCN lead to increase in Na^+^ influx (see Eq 25, CM).

Activation of MR dependent NALCN channels in low glucose could be a mechanism by which acetylcholine increases human insulin secretion during first phase of feeding where an increase in insulin secretion precedes an increase in blood glucose. This increase, termed "cephalic phase", is thought to be largely mediated by the release of acetylcholine from nerves innervating pancreatic islets or from the alpha cells [6, 62, 180]. In this case activation of MRs may increase the capability for insulin secretion even while still in sub-threshold glucose levels.

Zawalich and colleagues [115] showed that the failure of PLC activation resulted in impairment of insulin secretion under sustained glucose exposure in rats. It was also found that mice that selectively lacked MRs in pancreatic β-cells showed decreased insulin release and impaired glucose tolerance. Consistent with this concept, transgenic mice that selectively overexpressed type 3 MRs in pancreatic β-cells exhibited increased insulin release and improved glucose tolerance [181]. Our simulation with a decreased number of MRs leads to decreased [Ca^2+^]_c_ and DAG concentration at high glucose because constitutive MR activity takes part in IP_3_ and DAG production. For example, our simulation with 10x reduced MR abundance (to compare with the example in Fig 3) shows a significant decrease in [Ca^2+^]_c_ (from 272 to 250 nM) and DAG concentration (from 1131 to 926 #/μm^-2^) at high glucose. The "cephalic phase" should also be blocked in these conditions. This should lead to decreased insulin secretion. These experimental data and our simulations suggest that optimal acetylcholine receptor stimulation is essential for β-cell insulin secretion.

Approaches aimed at enhancing signaling through β-cell MRs (or downstream) might become therapeutically useful for the treatment of T2D (see [104, 182]). However, our consideration shows that MR activation could conceivably lead to hypoglycemia at relatively low glucose concentrations as a consequence of NALCN channel activation leading to PM depolarization and increased [Ca^2+^]_c_.

#### FFAR1/GPR40 and muscarinic receptor interactions

The temporal interplay between FFAR1/GPR40 and muscarinic receptors was investigated [37]. Palmitic acid at physiological levels could acutely inhibit subsequent acetylcholine-stimulated insulin secretion (through activation of FFAR1/GPR40) and the rise of intracellular Ca^2+^ production in isolated mouse islets during GSIS. According to these authors the mechanism of such effect could be a Ca^2+^ release from the ER during FFAR1/GPR40 activation that prevents subsequent acetylcholine induced Ca^2+^ release. Our simulation shows that this effect can take place, i.e. FFAR1/GPR40 activation can prevent additional [Ca^2+^]_ER_ decrease and Ca^2+^ release at acetylcholine administration (Fig 8A and 8B). However, there is also competition at the level of PLC such that activation of FFAR1/GPR40 leads to G-proteins binding to PLC_p_ that decreases the ability of PLC_p_ to bind with G-proteins, activated by acetylcholine (Fig 8D). Consequently, simultaneous use of activators for FFAR1/GPR40 and the muscarinic receptor may not lead to significant additive effects on insulin secretion.

### Role of phosphoinositides

The rate of phosphoinositide metabolism is increased in glucose-stimulated islets. This effect is due to PLC-mediated hydrolysis of PIP_2_ and leads to a decrease in PIP_2_ content and an increase in P4P [183]. Increased glucose leads also to activation of PLC and decreased PIP_2_. An increase in P4P in our model is a consequence of PKC and Ca^2+^ mediated activation of phosphatidylinositol 4-kinase (see Fig 1 and Fig 3E).

We modeled block of K_ATP_ channels due to decreased PIP_2_ (see Eq 10, CM). This can cause additional PM depolarization and an elevated level of [Ca^2+^]_c_ thogether increasing the insulin secretion rate. These simulations reflect data that shows disrupting the interaction between K_ATP_ channels and PIP_2_ by overexpressing Kir6.2 in mutants with decreased sensitivity to PIP_2_ causes persistent membrane depolarization and elevated basal level insulin secretion [184]. Additionally, PLC activation can also simulate closure of K_ATP_ channel as a consequence of a decreased PIP_2_ concentration [41, 42].

Glucose-induced P4P elevation required voltage-gated Ca^2+^ entry and was mimicked by membrane-depolarizing stimuli. P4P elevation was also sensitive to PKC inhibition and mimicked by phorbol ester stimulation [96]. We were able to simulate these results because P4P synthesis is activated by PKC (see Eq 74 in CM and Figs 1 and 8E). Our model confirms that PIP_2_ levels in β cells are regulated for proper insulin exocytosis [96, 183].

### Interaction of pathways

Activation of the GLP-1R, GIPR, MR and FFAR1/GPR40 receptors following meal ingestion could be simultaneous, very closely timed or even sequential. For this reason it is important to evaluate cross talk between GPCR agonists, comparing and combining those that function via the G_αs_ pathway (e.g., GLP-1 and GIP) with those that function via the Gα_q/11_ pathway (e.g. acetylcholine and FFA or FFAR1/GPR40 agonists).

**Fig 10:**
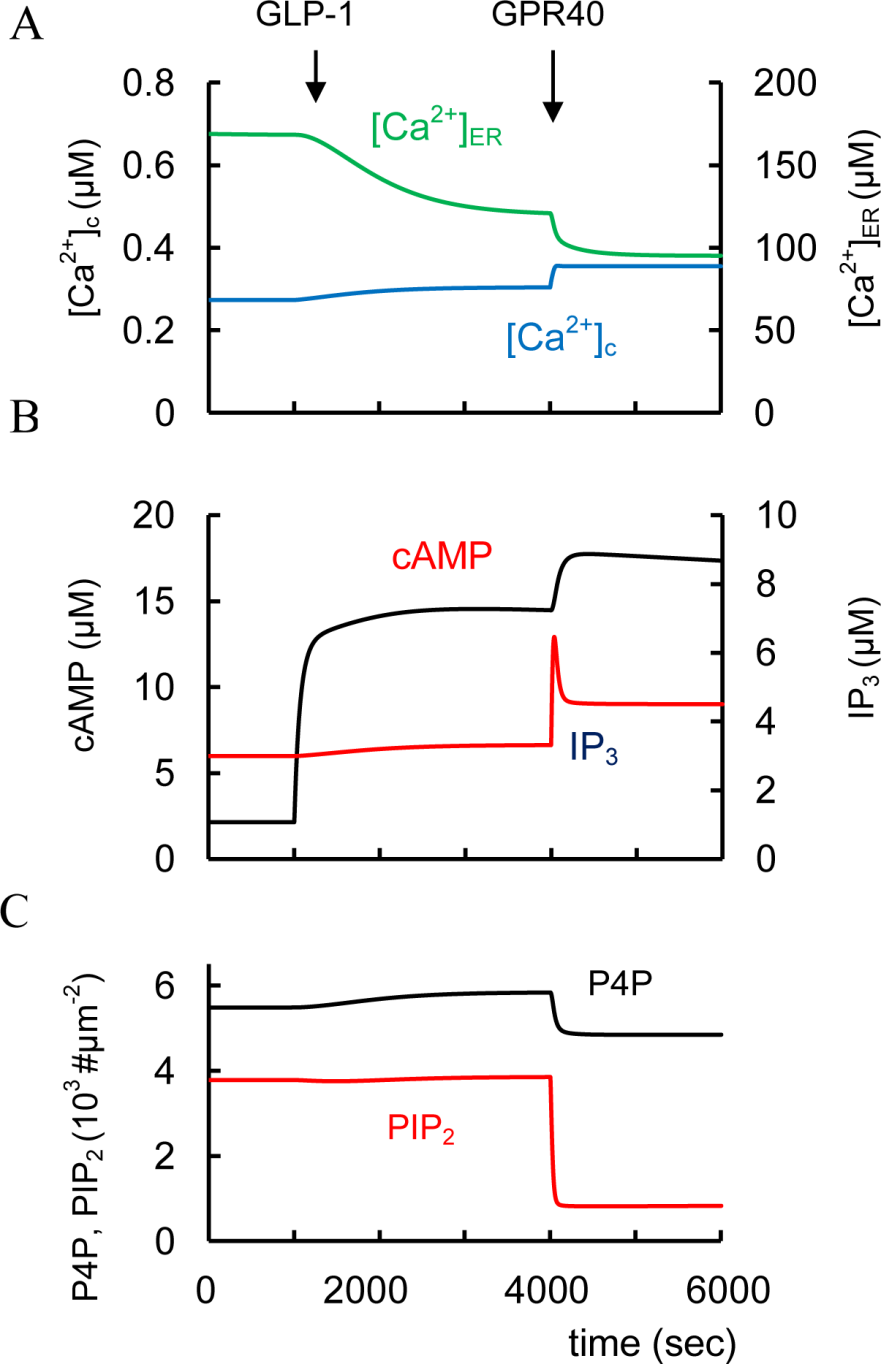
Simulated responses to GLP-1 and FFAR1/GPR40 activator at high glucose. (A) [Ca^2+^]_c_ and [Ca^2+^]_ER_; (B) Cytoplasmic cAMP and IP_3_ concentrations; (C) Concentrations of P4P and PIP_2_ on PM. Glucose (8 mM) induced changes of intracellular parameters were initially simulated as in Fig 3 up to steady state (left part). GLP-1 was simulated at 1000 sec as an increased GLP-1 from basal level (3.1e-7 μM) to 6.2e-4 μM. After that FFAR1/GPR40 activation was simulated at 4000 sec as increased AR_7_ from basal level (0.0506 μM) to 82 μM.

Interestingly, activation of the cAMP pathway can activate the PLC pathway. For example, GLP-1 can activate PKC through Ca^2+^-dependent activation of PLC in the insulin-secreting rat β-cell line INS-1 [185]. Indeed, according to our calculations an activation of the cAMP pathway (such as GLP-1 addition) leads to PKA activation and corresponding closure of the K_ATP_ channels and opening of IP_3_R leading to an significant increase in [Ca^2+^]_c_ (from 0.272 to 0.302 μM) (Fig 10A). This activates Ca^2+^-dependent PLC leading to an increase of IP_3_ (and DAG) concentrations (see Fig 10B).

**Fig 11:**
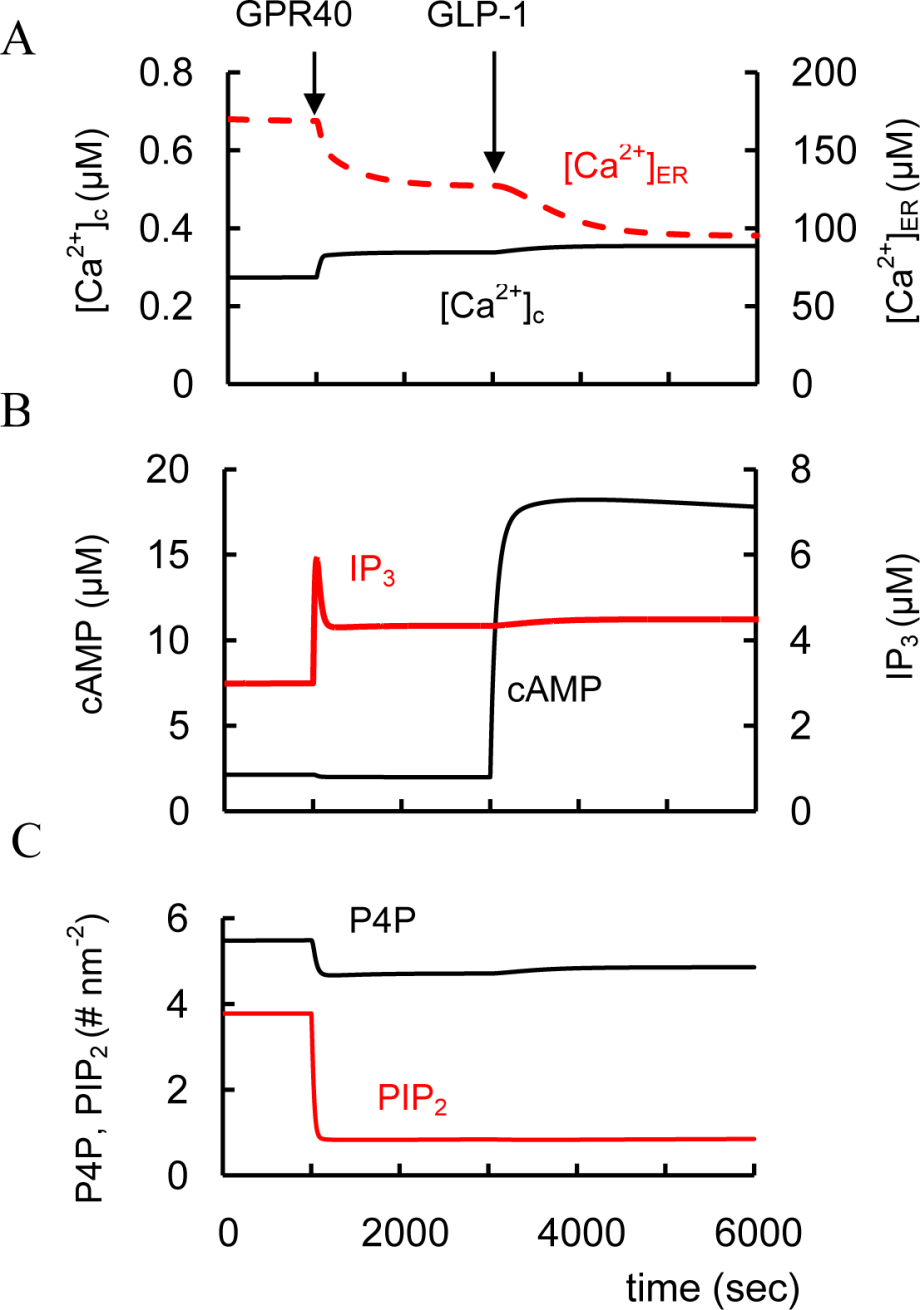
Simulated response to FFAR1/GPR40 activator with following addition GLP-1 at high glucose. (A) [Ca^2+^]_c_ and [Ca^2+^]_ER_; (B) Cytoplasmic cAMP and IP_3_ concentrations; (C) Concentrations of P4P and PIP_2_ on PM. Glucose (8 mM) induced changes of intracellular parameters were initially simulated as in Fig 3 up to steady state (left part). FFAR1/GPR40 activator was simulated at 1000 sec as an increased AR_7_ from basal level (0.0506 μM) to 82 μM. After than GLP-1 action was simulated at 3000 sec (GLP-1 increased from basal level (3.1e-7 μM) to 6.2e-4 μM).

On other hand, if FFAR1/GPR40 activators are employed first, to activate the PLC pathway, this does not lead to an increase in cAMP concentration (see Fig 11B). In this case FFAR1/GPR40 activation significantly increases [Ca^2+^]_c_, IP_3_ and DAG but it does not effect cAMP concentration. According to our model (that was constructed mainly from mouse islets data) cAMP concentrationis not increased because this would occur only by activation of PM bound AC by specific G-protein coupled receptors (such as GLP-1R or GIPR). Indeed agonist activation of the G_q/11_ receptor (i.e. an activation of PLC pathway) has no significant effect on intracellular cAMP level in mouse islets [186, 187].

However, the effect of activation of the PLC pathway on the cAMP pathway may be species dependent. For example, a different behavior was found in rat β-cells. In isolated rat islets the MR agonist carbamylcholine chloride (CCh) evoked a concentration-dependent increase in cAMP generation with a maximum at least 4.5-fold above control even at basal 2.8 mmol/l glucose. However, in this case activation of the PLC pathway may only partially activate the cAMP pathway. This was “partial” because forskolin and GLP-1 increased cAMP accumulation 10-fold and 23-fold, respectively [188], i.e. G-protein dependent AC activity plays a significant role in rat β-cells. Interestingly, acetylcholine acts predominately through activation of the cAMP/PKA pathway alone (no increase in the IP3 pathway) to enhance Ca^2+^-stimulated insulin release islets in the Goto-Kakizaki β-cell, a spontaneous rat model of T2D [189] although this finding has not been replicated.

**Fig 12:**
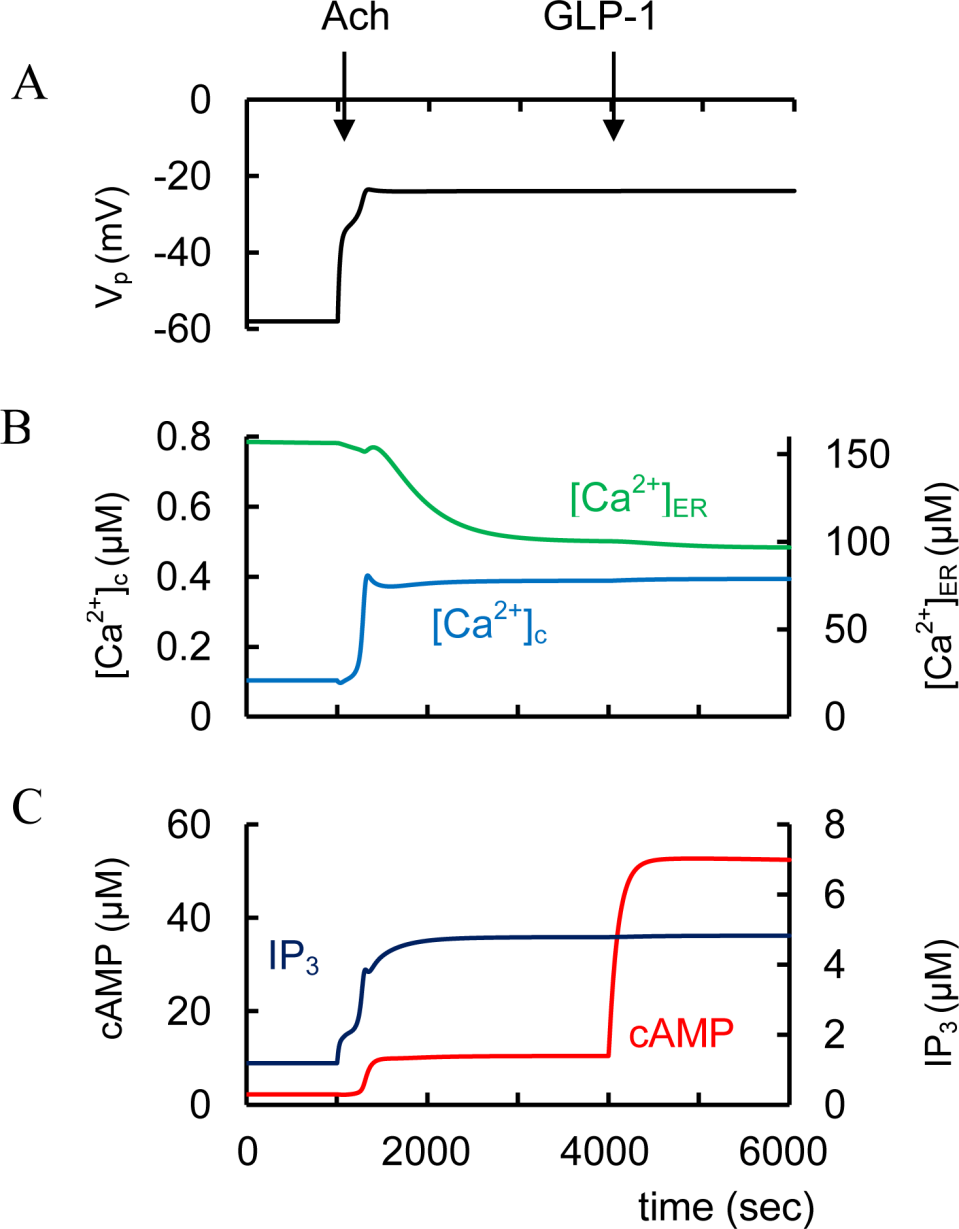
Simulated response to Ach at activated NALCN channels and decreased phosphodiesterase (PDE) activity in low glucose. (A) PM potentials (V_p_); (B) [Ca^2+^]_c_ and [Ca^2+^]_ER_; (C) Cytoplasmic cAMP and IP_3_ concentrations. For simulation of NALCN channel activity the maximal conductance (g_mNM3_, Eq 25, CM) was initially increased from 0 nS (basal level) to 35 nS. PDE activity (Vcpde, Eq 66) was decreased from 1.4 μmol s^-1^ (basal level) to 0.3 μmol s^-1^. All simulations were performed at low glucose level (3 mM).

We used our model to propose a hypothesis that can explain these experimental data. For example, our simulation showed that increased cAMP following activation of the PLC pathway may be due to the presence in rat of mainly PDE forms that are not activated by [Ca^2+^]_c_ (Fig 12). In this case increased [Ca^2+^]_c_ following acetylcholine MR activation (for example by activation of NALCN channels at low glucose accelerates the rate of cAMP production but not degradation, because the maximal activity in Ca^2+^/CaM activated PDE was decreased, leading to a significant increase in cAMP. cAMP increases 4.8 fold following simulation of the MR receptor activation and 24-fold with additional GLP-1 administration, compared to cAMP levels without agonists (Fig 12C), that corresponds to experimental data [188]. We could not find any information about the behavior of cAMP and IP_3_ in human β-cells related to possible interactions of the cAMP and PLC pathways. This question demands further investigation. However, our theoretical analysis shows the possible mechanisms and consequences of potential interactions.

In general, according to these simulations, simultaneous activation of the cAMP and PLC pathways should increase GSIS. This follows increased second messengers such as [Ca^2+^]_c_, cAMP and DAG that can activate an insulin secretion. In other words, receptor agonists for cAMP and PLC pathways can act cooperatively (for example see Figs 10 and 11). Indeed, the combination of GLP-1 and acetylcholine increased insulin release compared with each compound alone during an acute test in the β-cell line BRIN BD11 [190] and in the isolated perfused rat pancreas [191]. FFAR1/GPR40 and GLP-1 receptor agonists markedly increased GSIS and their combination led to GSIS that was significantly increased in comparison with each compound alone [166]. These data are in accord with our simulations. Interestingly, FFAR1/GPR40 and GLP-1 receptor agonists as well as their combination did not increase insulin secretion at low glucose in islets isolated from normal rats [166]. This could be the result of a inadequate activation of AC and PLC at low [Ca^2+^]_c_.

## Limitations and Perspectives

Interpretation of the effects of G-protein blockers and activators on β-cells is difficult because a change of any messenger potentially alters several aspects of the messenger dynamics of other messenger pathways. For this reason, using a mathematical modeling approach opens up significant new possibilities and allows a better understanding of potential messenger interactions. Our model represents significant improvements in a number of areas of β-cell computational simulation. It was possible to roughly determine the contribution of each messenger pathway to [Ca^2+^]_c_ and insulin secretion dynamics under specific conditions. Our simulations confirm that the specific messenger dynamics interactions can lead to an appropriate increase in insulin secretion.

The model we developed here must also be considered in the context of known limitations. Some limitations are unavoidable because of the limited current knowledge of basic biochemical and physiological data, the extensive variability among experimental data, the considerable variation in experimental conditions, and the potentially deleterious effects inherent in experiments with β-cells. For example, the actual variations in the number of receptors, G-proteins and target enzymes during signaling as well as the mechanisms of their interactions are not known.

These limitations required some formulations from other models based on animal experiments to be used. Therefore it would be unreasonable to expect the model to accurately reproduce all previously observed experimental results. However, we were able to show that the model we developed can reproduce a number of experimental observations ranging from G-protein coupled receptors to Ca^2+^ and insulin secretion dynamics. It can be used as the groundwork for *in silico* examination of the effects of agents that modulate insulin secretion via regulation of GPCRs. As other receptors are further characterized they can be readily incorporated into this basic model. This creates opportunities for *in silico* studies of possible functional influence of different agents on β–cell functional properties that it is difficult to evaluate at this time.

The data used to fit the parameters of our model were taken primarily from mouse islets and cell lines. For this reason the simulations of the experimental results obtained with rat and human β-cells can present difficulties. Some differences between species were stressed above. However, more data is clearly needed to analyze the human cells and islets.

Other limitations are related to mechanisms of insulin secretion. Insulin is secreted by exocytosis of large dense-core vesicles. Regulation of insulin–containing granule secretion in β-cells is a complex function of cytoplasmic Ca^2+^, cAMP and the PLC pathways. Several mathematical models were developed for describing exocytosis of insulin granules (see for review [25, 192]). However, our knowledge of the molecular regulation of exocytosis in β-cells remains fragmentary. A detailed consideration and modeling of the effects of additional regulatory pathways on the rate of exocytosis of insulin–containing granules is needed.

## Conclusion

The multiple receptors expressed by β-cell pass their signals through a common set of downstream effectors distinguished by multiple isoforms with different specificities and activities. The coupling among these pathways causes interactions among the signals sent by the different classes of receptors. We have developed a computational integrated model of the G-protein signal transduction system from the receptors to the intracellular second-messengers: Ca^2+^, PIP_2_, cAMP, DAG and IP_3_. The model includes detailed descriptions of interactions between G-protein-coupled-receptors, [Ca^2+^]_c_, Ca^2+^-bound calmodulin (Ca^2+^/CaM), adenylyl cyclase, phosphodiesterase and PLC pathway regulation. We used this model to predict the signal processing of several G-protein-coupled-receptors.

By combining experimental data on G-protein and second messenger interactions with mathematical modeling, our systems approach is likely yield a detailed analysis of metabolic and signaling pathway interactions in the β-cell and help elucidate key regulatory mechanisms involved in regulation of insulin secretion. The simulations provide a way to compare the time-course and consequence of changes in expression of mechanisms that regulate cytoplasmic Ca^2+^ relative to the activation of cAMP and PLC pathways and to evaluate hypotheses on regulatory mechanisms of insulin secretion.

The most important results of our system biology computational analysis are:

1. The concentration of incretin hormone receptors in the PM can be a key limiting factor in mechanisms leading to activation of insulin secretion by incretins.
2. There can considerable variability in the effects of acetylcholine and GLP-1, GIP or FFAR1/GPR40 agonists on insulin secretion. Our theoretical analysis shows that activators of GLP-1, GIP and FFAR1/GPR40 receptors may have a low risk of hypoglycemia because they need a concomitant increase in [Ca^2+^]_c_ for activation whereas an activator of muscarinic receptors can lead to hypoglycemia. This is a consequence of an activation of specific channels, leading to depolarization of the PM and [Ca^2+^]_c_ increase even at lower blood glucose levels.
3. G-coupled receptor activators can have additive effects on insulin secretion if they act on different messenger pathways (i.e. cAMP and PLC pathway in our case). The effectors may not have any additive interactions if they act on the same pathway (for example the pair GLP-1 and GIP or FFAR1/GPR40 activators and acetylcholine).
4. The modeling of messenger pathway interactions is important for determining the pharmacological targets for improving insulin secretion in T2D. The results of the model applications can guide clinical approaches to improvement of β-cell function by combinations of stimuli via GPCR agonists, helping to minimize side effects and maximize clinical benefit.

## Computational Model (CM)

The purpose of this section is to develop the mathematical formalism for a β-cell model of receptors and second messenger interactions. A schematic diagram of the biochemical steps, channels, receptors, Ca^2+^ handling and messenger pathways in β-cell is presented in Fig 1. We will highlight some basic assumptions and general properties of the model reflecting the main mechanisms describing in text. We assume concentration of most species lies in the range that allows us to avoid the need to use complex stochastic algorithms that would be necessary if regulatory processes are dependent on minute quantities of molecules. The β-cell is modeled as a small spherical cell. Modeling [24] has shown that in such a cell, for example, cAMP is distributed rapidly and uniformly throughout the cytoplasm, even following GLP-1 receptor activation, in time intervals that we used in our simulations (minutes). On this base we suggested that species are distributed uniformly on PM, in cytoplasm or ER of β-cells in our models. Certainly some molecular events are critically dependent on localized gradients such as possible activation of insulin granule release by Ca^2+^ near specific Ca^2+^ channels [193], however these potentially important events are not considered in our model.

### Approach

The present model is created as systems of ordinary differential equations with nonlinear terms. Numerical integration was carried out using standard numerical methods. Three compartments: plasma membrane, cytoplasm and ER were used. The units, except where indicated otherwise: cytosolic and ER species and ligand concentrations are represented by concentration in (*μ*M) while membrane-bound species are given as area densities (number per unit of membrane area) in (# μm^−2^), time in seconds (s), voltage in millivolts (mV), current in femtoamperes (fA), conductance in picosiemens (pS), capacitance in femtofarads (fF) (per cell).

Experimental data are often represented only for concentration per volume and it is often nessesary to calculate a concentration on PM surface. Then the coefficient (f_Nc_) to convert the concentration from # μm^-2^ to μM for β-cell can be calculated as

$${\text{f}}_{\text{Nc}}=10^{6}\;{\text{S}}_{\text{c}}/({\text{N}}_{\text{av}}\;{\text{V}}_{\text{c}})$$ (1)

where S_c_ is the cell surface, V_c_ is the volume of the cell cytoplasm (in liter) and N_av_ is Avogadro’s constant. f_Nc_ = 0.00211 μM μm^-2^ if the data from Table 1 were used.

**Table 1:** Cell and physical parameters.

Parameter	Value (units)	Eq.	Ref.
f_nc_	0.00211 μM μm^2^	1	Ad
C_m_	6158 fF	8	[33]
V_c_	764 μm^3^	1	[194]
S_c_	973 μm^2^	1	[194]
V_ER_	280 μm^3^	87	[194]
f_cf_	0.01	86	[33]
f_ER_	0.03	87	[33]
F	96.487 C μM^-1^	86	
N_av_	6.022 10^23^ mol^-1^	1	

Ad, adjusted to fit the integrated experimental values for whole system.

Lifetimes are often only accessible from experimental articles. Assuming the first-order processes, the detected lifetimes (t_1/2_) can be converted to rate constants according to equation k = ln2/t_1/2_ = 0.693/ t_1/2_.

The data used to fit the computational model in our study were taken primarily from isolated rodent β-cells and cell lines. Some parameters were taken from the literature. Remaining parameters were experimentally unreachable and we optimized their values by simulated the model and compare with experimental data (Tables 1–6). For simplicity, we use the concentrations the components inside a cell without brackets. Basal initial conditions were denoted as (-in).

**Table 2:** Cell metabolism and membrane current parameters.

Parameter	Value (units)	Eq.	Ref.
ATD___in	1.5	2	Ad
t_ATD_	100 s	2	Ad
ATD_m_	32	4	Ad
K_GF_	5200 μM	4	[195]
k_GFr_	7.4	4	Ad
hgla	5	4	[195]
FFA	0 μM	4	Ad
AT	4000 μM	5	[33]
V_P__in	-62 mV	8	Ad
g_mKATPi_	24,000 pS	9	[33]
E_K_	-75 mV	9	[29]
K_KPI2_	1125 # μm^-2^	10	[38]
k_dd_	17 μM	11	[29]
k_td_	26 μM	11	[29]
k_tt_	50 μM	11	[29]
k_KPKa_	1.	13	Ad
g_mKr_	45000 pS	15	[29]
V_dKr_	-9 mV	16	[29]
k_dKr_	5 mV	16	[29]
g_mCa_	900 pS	18	Ad
E_Ca_	100 mV	18	[29]
V_dCa_	-19 mV	19	[33]
k_dCa_	9.5 mV	19	[33]
K_dCap_	0.01	19	Ad
V_fCa_	-9 mV	20	[29]
k_fCa_	8 mV	20	[29]
gmSOC	10 pS	21	Ad
K_NS_	200 μM	22	[196]
P_mCa_	6000 fA	23	Ad
K_pCa_	0.2 μM	23	Ad
E_Na_	70 mV	24	[29]
g_mNb_	10 pS	25	Ad
gmNM3	0 pS	25	Ad

Ad, adjusted to fit the integrated experimental values for whole system.

**Table 3:** Initial values and coefficients for receptors and G-proteins.

Parameter (units)	Initial values and coefficients (Eq 46)		Initial values and coefficients (Eq 47)		Initial values and coefficients (Eq 48)	
Ligand (L_n_, μM)	GLP1i	3.1 10^−7^	GIPi	1.37 10^−6^	AR3i	0.002
Re_nd_ (# μm^-2^)	ReGLd_in	0.1	ReGId_in	0.1	ReARd_in	0.1
L_n_R_n_ (# μm^-2^)	LR1_in	0.01	LR2_in	0.01	LR3_in	0.01
L_n_R_n_G_n_ (# μm^-2^)	LRG1_in	0.01	LRG2_in	0.01	LRG3_in	0.01
G_αnGTP_ (# μm^-2^)	GaT1_in	0.01	GaT2_in	0.01	GaT3_in	0.01
G_αnGDP_ (# μm^-2^)	GaD1_in	0.01	GaD2_in	0.01	GaD3_in	0.01
G_αnGTP_E_n_ (#μm^2^)	AC_GLP__in	0.3	AC_GIP__in	0.3	AC_AR__in	0.3
Re_nt_ (# μm^-2^)	Re_GLt_	2	Re_GIt_	2.5	Re_ARt_	3
G_nt_ (# μm^-2^)	G1t	20	G2t	25	G3t	20
E_nt_ (#μm^-2^)	AC_pt_	3	AC_pt_	3	AC_pt_	3
K_L_ (μM)	K_GLP1_	3.1 10^−5^	K_GIP_	1.71 10^−4^	K_AR3_	0.3
k_1n_ (μM^-1^ s^-1^)	R11	7	R21	7	R31	7
k_2n_ (#^-1^ μm^2^ s^-1^)	R12	1	R22	1	R32	1
k_2nr_ (s^-1^)	R12r	0.68	R22r	0.68	R32r	0.68
k_3n_ (s^-1^)	R13	0.7	R23	0.7	R33	0.7
k_4n_ (#^-1^ μm^2^ s^-1^)	R14	1	R24	1	R34	1
k_5n_ (s^-1^)	R15	0.026	R25	0.026	R35	0.026
k_6n_ (s^-1^)	R16	0.4	R26	0.4	R36	0.4
k_7n_ (#^-1^ μm^2^ s^-1^)	R17	1.	R27	1	R37	1
k_8n_ (s^-1^)	R18	0.00005	R28	0.00005	R38	0.00005
k_9n_ (s^-1^)	R19	2.83 10^−4^	R29	2.83 10^−4^	R39	2.83 10^−4^

**Table 4:** Initial values and coefficients for receptors and G-proteins (continuation).

Parameter (Units)	Initial value and coefficients (Eq 49)		Initial value and coefficients (Eq 50)	
Ligand (L_n_) (μM)	AM3i	0.022	AR7i	0.0506
R_n_G_n_ (#μm^-2^)	RG6_in	0.01	NA	NA
R_nd_ (# μm^-2^)	Re_M3d__in	0.1	Re_R40d__in	0.1
L_n_R_n_ (# μm^-2^)	NA	NA	LR7_in	0.01
L_n_R_n_G_n_ (# μm^-2^)	LRG6_in	0.01	LRG7_in	0.01
G_αnGTP_(# μm^-2^)	GaT6_in	0.01	GaT7_in	0.01
G_anGDT_ (# μm^-2^)	G_a_D6_in	0.01	GaD7_in	0.01
G_αnGTP_E_n_ (# μm^-2^)	PLC_M3__in	0.1	PLC_R40__in	0.1
R_nt_ (# μm^-2^)	Re_M3t_	2	Re_R40t_	2
G_nt_ (# μm^-2^)	G6t	20	G7_t_	20
E_nt_ (# μm^-2^)	PLC_pt_	3	PLC_pt_	3
K_M_ (μM)	K_AM3_	0.22	K_AR7_	4.6
k_1n_ (μM^-1^ s^-1^)	R61	10	R71	7
k_2n_ (#^-1^ μm^2^s^-1^)	R62	0.68	R72	1
k_2nr_ (s^-1^)	R62r	6.8	R72r	0.68
k_3n_ (s^-1^)	R63	0.65	R73	0.7
k_4n_ (#^-1^ μm^2^ s^-1^)	R64	1	R74	1
k_5n_ (s^-1^)	R65	0.026	R75	0.026
k_6n_ (s^-1^)	R66	0.4	R76	0.4
k_7n_ (#^-1^ μm^2^ s^-1^)	R67	1	R77	1
k_8n_ (s^-1^)	R68	0.007	R78	5 10^−5^
k_9n_ (s^-1^)	R69	0.00105	R79	2.83 10^−4^

**Table 5:** Parameters and coefficients for calmodulin and cAMP pathways.

Parameter	Value (units)	Eq.	Ref.
CaCaM_in	0.42 μM	51	[25]
k_1f_	2.3 10^3^ μM s^-1^	51	[23]
k_1b_	2.4 10^3^ s^-1^	51	[23]
k_2f_	2.3 10^3^ μM s^-1^	52	[23]
k_2b_	2.4 10^3^s^-1^	52	[23]
k_3f_	160 10^3^ μM s^-1^	53	[23]
k_3b_	405 10^3^ s^-1^	53	[23]
k_4f_	160 10^3^ μM s^-1^	54	[23]
k_4b_	405 10^3^ s^-1^	54	[23]
CaMo	11.25 μM	55	[23]
kda	0.01	57	Ad
AC_pt_	3 # μm^-2^	59	Ad
V_mCaM_	2 μmol s^-1^	63	Ad
K_PCaM_	0.348 μM	64	[23]
K_NCa_	75 μM	64	[23]
V_mACc_	0.2 μmol s^-1^	65	Ad
K_mAACS_	1030 μM	65	[197]
K_mCACS_	0.5 μM	65	Ad
k_ACS_	0.01 μmol s^-1^	65	Ad
k_ipdei_	1	66	Ad
V_gpde_	0.04 μmol s^-1^	66	[23]
V_cpde_	1.4 μmol s^-1^	66	Ad
K_dpe_	0.348 μM	66	[23]
K_pde_	3 μM	66	[23]
PKAa_in	0.24	67	[25]
k_ak_	1	67	Ad
t_pka_	900 s	67	[198]
K_pcm_	2.9 μM	69	[24]
hpca	1.4	69	[24]
EPa_in	0.01	70	Ad
t_ep_	900 s	70	[25]
K_mep_	20.2 μM	72	[199]
hce	2	72	Ad

**Table 6:** Parameters and coefficients for PLC pathway and Ca^2+^ handling.

Parameter	Value (units)	Eq.	Ref.
P4P_in	4000 # μm^-2^	73	[200]
k_PI_	0.0015s^-1^	73	Ad
k_PIr_	0.006 s^-1^	73	[200]
k_P4P_	0.02 s^-1^	73	[200]
k_P4Pr_	0.014 s^-1^	73	[200]
PI	140,000 # μm^-2^	73	[201]
K_CaPI_	0.3	74	Ad
K_P4PK_	0.5	74	Ad
k_cpi_	0.2	74	Ad
PIP2_in	4200 # μm^-2^	75	Ad
K_PIP2_	2370 # μm^-2^	76	Ad
k_pPL_	15 μmol s^-1^	77	Ad
V_mPLPi_	700 μmol s^-1^	78	Ad
K_CaPL_	0.4 μM	78	[33]
V_mPLC_	50 μmol s^-1^	81	Ad
K_CCaPL_	0.2 μM	81	Ad
IP_3__in	1 μM	82	Ad
*k*_dIP3_	0.04 s^-1^	82	[33]
DAG_in	23 # μm^-2^	83	[200]
*k*_dDAG_	0.05 s^−1^	83	[200]
PKCa_in	0.1	84	Ad
k_PKC_	3E-6 s^-1^	84	Ad
k_PKCr_	0.0034 **s**^**-1**^	84	[202]
Cac_in	0.09 μM	86	Ad
*k*_sg_	0.00001 s^−1^	86	Ad
Ca_ER__in	160 μM	87	Ad
P_CaER_	6 μmol s^-1^	88	Ad
K_ser_	0.4 μM	88	Ad
k_mIP_	7 μM s^-1^	89	Ad
k_leak_	0.002 s^-1^	89	Ad
K_RPCa_	0.35 μM	90	Ad
K_IP3_	3.2 μM	90	[33]
K_IP3R_	0.5	90	Ad

### Glucose metabolism

The influence of glucose (Glu) and FFA on GSIS was modeled as the dynamical changes in [ATP]/[ADP] ratio that determines K_ATP_ channel opening. We assume that the nucleotide ratio satisfies the first-order kinetic (see [203]).

$$\text{d ATD}\;/\text{dt}=({\text{ATD}}_{\text{o}}\;-\;\text{ATD})/{\text{t}}_{\text{ATD}}$$ (2)

where

ATD=ATP/ADP (3)

$${\text{ATD}}_{\text{o}}=\frac{{\text{ATD}}_{\text{m}}\;{\left(\text{Glu}+{\text{k}}_{\text{GFr}}\;\text{FFA}\right)}^{\text{hgla}}}{{\left(\text{Glu}+{\text{k}}_{\text{GFr}}\;\text{FFA}\right)}^{\text{hgla}}+{{\text{K}}_{\text{GF}}}^{\text{hgla}}}$$ (4)

$$\text{AT}=\text{ATP}+{\text{ADP}}_{\text{f}}$$ (5)

$${\text{ADP}}_{\text{f}}=\text{AT}/(1+\text{ATD})$$ (6)

and

$$\text{Glu}={\text{Glu}}_{\text{i}}+\text{Glu}1\;(\text{t}>\text{t}1)$$ (7)

where ADP_f_ and ATP are concentrations of free ADP and ATP in cytoplasm, Glu and FFA are the concentrations of glucose and free fatty acid in surrounding medium. ATD_o_ is the steady-state ATP/ADP ratio. Glu_i_ is the initial Glu concentration. Glu1 is the change in Glu in time (t1).

The dependence of ATD_o_ on extracellular concentration of glucose and FFA (Eq 4) was suggested as a Hill function with saturated value (ATD_m_), K_GF_ is the coefficient for half-saturated glucose and FFA concentrations, hgla is the the Hill coefficient, k_GFr_ is the coefficient for a conversion from FFA to glucose concentration, t_ATD_ is the time constant (Table 2).

The coefficients K_GF_ and hgla were taken from work [195] where these data were evaluated for human β-cell. The scaling coefficient k_GFr_ was evaluated using data for FFAR1/GPR40 knockout mice because FFA can also activate FFAR1/GPR40 receptor affecting on GSIS. According to Fig 5A from [204] insulin secretion at 8.3 mM glucose and 0.5 mM palmitate is about the same as for 12 mM glucose in FFAR1/GPR40 knockout mice islets. In this case k_GFr_ = 7.4 (example calculated for palmitate concentration).

### Membrane potential, channels and pumps

#### Plasma membrane potential

Increased extracellular glucose promotes membrane depolarization. Several ionic channels regulate this process. A diagram of the main β-cell specific channels considered here is presented in Fig 1. We used our models of electrophysiological processes in the β-cell [29, 30, 33] as a framework. However, only the most important currents were taken into account to achieve the desired granularity. For simplification Boltzman-type equations in steady-state were employed for activation (d_i_) and inactivation functions (f_i_) to avoid a simulation of spike activity.

We modeled the electrophysiological events for one cell. The plasma membrane (PM) potential in a single β-cell can be described with the following current balance differential equation

$${\text{V}}_{\text{p}}/\text{dt}=-(I_{\text{KATP}}+I_{\text{Kr}}+I_{\text{Ca}}+I_{\text{SOC}}+I_{\text{PCa}}+I_{\text{Nab}})/{\text{C}}_{\text{m}}$$ (8)

where V_p_ is the PM potential, t is the time, C_m_ is the whole-cell membrane capacitance, *I*_KATP_ is the ATP-sensitive K^+^ channel current, *I*_Kr_ is the voltage-dependent K^+^ current, *I*_Ca_ is the high-voltage-activated Ca^2+^ current, *I*_SOC_ is the store-operated current activated by decrease of Ca^2+^ in ER, *I*_PCa_ is the plasma membrane Ca^2+^-pump current, *I*_Nab_ is the Na^+^ background current, that can be activated also by muscarinic acetylcholine M_3_ receptor.

#### ATP-sensitive K^+^ channels current (*I*_KATP_)

We adopted a kinetic model [29, 33] for whole-cell ATP-sensitive K^+^ channels current (*I*_KATP_). However, a dependence of this current on PIP_2_ and PKA activation was also found and we introduced the corresponding factors. The influence of PIP_2_ was represented as Michaelis-Menten function (f_KPI_, Eq 10) where PIP_2_ increase can enchance *I*_KATP_.

Both SUR1 and K_ir6.2_ subunits contain consensus PKA phophorylation sites and, for example, GLP-1 dependent activation of PKA leads to phosphorylation of SUR1 subunits lowering their affinity for ADP [205, 206]. For simplicity we introduced this dependence as a function (f_KPKa_, Eq 13) where an activated PKA (PKA_a_) decreases the calculated [MgADP_f_]_c_ leading to additional *I*_KATP_ decrease.

$$I_{\text{KATP}}={\text{g}}_{\text{mKATP}}\;{\text{f}}_{\text{KPI}}\;{\text{O}}_{\text{KATP}}\;({\text{V}}_{\text{P}}-{\text{E}}_{\text{K}})$$ (9)

where

$${\text{f}}_{\text{KPI}}={\text{PIP}}_{2}/({\text{PIP}}_{2}+{\text{K}}_{\text{KPI}2})$$ (10)

$${\text{O}}_{\text{KATP}}=\frac{0.08\;(1+2\;{\text{MgADP}}_{\text{f}}/{\text{k}}_{\text{dd}})+0.89\;{\left({\text{MgADP}}_{\text{f}}/\;{\text{k}}_{\text{dd}}\right)}^{2}}{{\left(1+{\text{MgADP}}_{\text{f}}/\;{\text{k}}_{\text{dd}}\right)}^{2}\;(1+0.45\;{\text{MgADP}}_{\text{f}}/\;{\text{k}}_{\text{td}}+{\text{ATP}}_{\text{f}}/\;{\text{k}}_{\text{tt}})}$$ (11)

$${\text{MgADP}}_{\text{f}}=0.055\;{\text{f}}_{\text{KPKa}}\;{\text{ADP}}_{\text{f}}$$ (12)

$${\text{f}}_{\text{KPKa}}=1/\;(1+{\text{k}}_{\text{KPKa}}\;{\text{PKA}}_{\text{a}})$$ (13)

$${\text{g}}_{\text{mKATP}}={\text{g}}_{\text{mKATPi}}+{\text{g}}_{\text{mKATP}1}\;(\text{t}>\text{t}9)$$ (14)

where g_mKATP_ is is the maximum conductance for *I*_KATP_, g_mKATPi_ is the initial conductance, g_mKATP1_ is the increase in conductance in time (t9). E_K_ is the reversal potential for K^+^ current, PIP_2_ is the PIP_2_ content on PM, K_KPI2_ is the activation constant, O_KATP_ is the fraction of open K_ATP_ channels, k_dd_, k_td_ and k_tt_ are the dissotiation coefficients, MgADP_f_ is free Mg-bound ADP, ATP_f_ and ADP_f_ are free ATP and ADP concentrations in cytoplasm; k_KPKa_ is the inhibition constant.

To determine K_KPI2_ (Eq 10) we suggest that f_KPI_ dependence corresponds to PIP_2_ dose-dependent activation of K_ATP_ channels (in absence GST-Syn-1A) where EC_50_ values for PIP2 was calculated as 2.38±0.81 μM [38] or 1125 #/μm^-2^ in our definition (see Eq 1).

#### Voltage-gated K^+^ current (*I*_Kr_)

Different voltage gated K^+^ currents (*I*_Kr_) were also registrated in β-cell. For example, delayed rectifier K^+^ current is main voltage-dependent K^+^ current in rodents [207]. This current activates at membrane potential near -30 mV and then increased with the applied voltage, an inactivation was negligible during at least 200 ms depolarization [208]. The Hodgkin-Huxley-type with stationary dependences for delayed rectifier K^+^ current and some corresponding coefficients were used from our previous mouse and human β-cell model to describe *I*_Kr_ [29, 30].

$$I_{\text{Kr}}={\text{g}}_{\text{mKr}}\;{{\text{d}}_{\text{Kri}}}^{2}\;{\text{f}}_{\text{Kri}}\;({\text{V}}_{\text{P}}-{\text{E}}_{\text{K}}),$$ (15)

where

$${\text{d}}_{\text{Kri}}=1/(1+\text{exp}[({\text{V}}_{\text{dKr}}-{\text{V}}_{\text{P}})/\;{\text{k}}_{\text{dKr}}])$$ (16)

$${\text{f}}_{\text{Kri}}=1$$ (17)

g_mKr_ is the maximum conductance for *I*_Kr_, V_dKr_ is the half-activation potential, k_dKr_ is the slope of half-activation potential.

#### Ca^2+^ current (*I*_Ca_)

*I*_Ca_ was modeled as L-type Ca^2+^ channel with one inactivation gating variables [29] without time dependent parameters

$$I_{\text{Ca}}\;={\text{g}}_{\text{mCa}}\;{\text{d}}_{\text{Cai}}\;{\text{f}}_{\text{Cai}}\;({\text{V}}_{\text{P}}-{\text{E}}_{\text{Ca}})$$ (18)

where

$${\text{d}}_{\text{Cai}}=1/(1+\text{exp}[({\text{V}}_{\text{dCa}}-{\text{V}}_{\text{P}})/{\text{k}}_{\text{dCa}}])\;+{\text{k}}_{\text{dCap}}$$ (19)

$${\text{f}}_{\text{Cai}}\;=1/\;(1+\text{exp}[-({\text{V}}_{\text{fCa}}-{\text{V}}_{\text{P}})/\;{\text{k}}_{\text{fCa}}]$$ (20)

where g_mCa_ is the maximum conductance for *I*_Ca_, E_Ca_ is the reversal potential for Ca^2+^ current, V_dCa_ is the half-activation potential, k_dCa_ is the slope of half-activation potential, k_dCap_ is the coefficient of the constitutive channel activity, V_fCa_ is the half-inactivation potential, k_fCa_ is the slope of half-inactivation potential.

#### Store-operated Ca^2+^ current (*I*_SOC_)

[Ca^2+^]_ER_ decrease activates the Ca^2+^ release-activated Ca^2+^ current through PM. Interestingly, in human Jurkat leukaemic T cells expressing an ER-targeted Ca^2+^ indicator, SOC channel activation follows the function of [Ca^2+^]_ER_, reaching half-maximum at ~200 μM with a Hill coefficient of ~4 [196]. We use these dependence and coefficients in our model (Table 2). We consider *I*_SOC_ as a voltage-independent Ca^2+^ inward current

$$I_{\text{SOC}}={\text{g}}_{\text{mSOC}}\;{\text{f}}_{\text{SOC}}\;({\text{V}}_{\text{p}}-{\text{E}}_{\text{Ca}})$$ (21)

where

$${\text{f}}_{\text{SOC}}={{\text{K}}_{\text{NS}}}^{4}\;/(\;{{\text{K}}_{\text{NS}}}^{4}+{{\left[{\text{Ca}}^{2+}\right]}_{\text{ER}}}^{4}\;)$$ (22)

g_mSOC_ is the maximum whole-cell conductance, *f*_SOC_ is the [Ca^2+^]_ER_ dependent function, *K*_NS_ is the [Ca^2+^]_ER_ inhibition constant.

#### Plasma membrane Ca^2+^ pump current (*I*_PCa_)

Ca^2+^ pumps provide an outward current and also contribute to V_p_. The corresponding equation was adapted from previous model [33]

$$I_{\text{PCa}}={\text{P}}_{\text{mCa}}{{\left[{\text{Ca}}^{2+}\right]}_{\text{c}}}^{2}\;/(\;{{\text{K}}_{\text{pCa}}}^{2}+{{\left[{\text{Ca}}^{2+}\right]}_{\text{c}}}^{2}\;)$$ (23)

where P_mCa_ is the maximum *I*_PCa_ current, K_pCa_ is the [Ca^2+^]_c_ activation constant.

#### Na^+^ background current (*I*_Nab_)

The model contains a voltage-independent Na^+^ background current. This current can depolarize the resting PM potential and modifies mouse and human β-cell electrical activity (see for details [29, 30, 33]). We have also included the specific NALCN channels, that activatable by the muscarinic acetylcholine M_3_ receptor. Than

$$I_{\text{Nab}}={\text{g}}_{\text{Nab}}\;({\text{V}}_{\text{P}}-{\text{E}}_{\text{Na}})$$ (24)

where

$${\text{g}}_{\text{Nab}}={\text{g}}_{\text{mNb}}+{\text{g}}_{\text{mNM}3}\;{\text{LRG}}_{6}$$ (25)

E_Na_ is the reversal potential for Na^+^, g_mNb_ is the permanent conductance for *I*_Nab_, g_mNM3_ is the maximum conductivity of NALCN channels and LRG_6_ is the M_3_ receptor bound with ligand and G-protein (see below).

### Receptors, G-proteins and target enzymes

**Fig 13:**
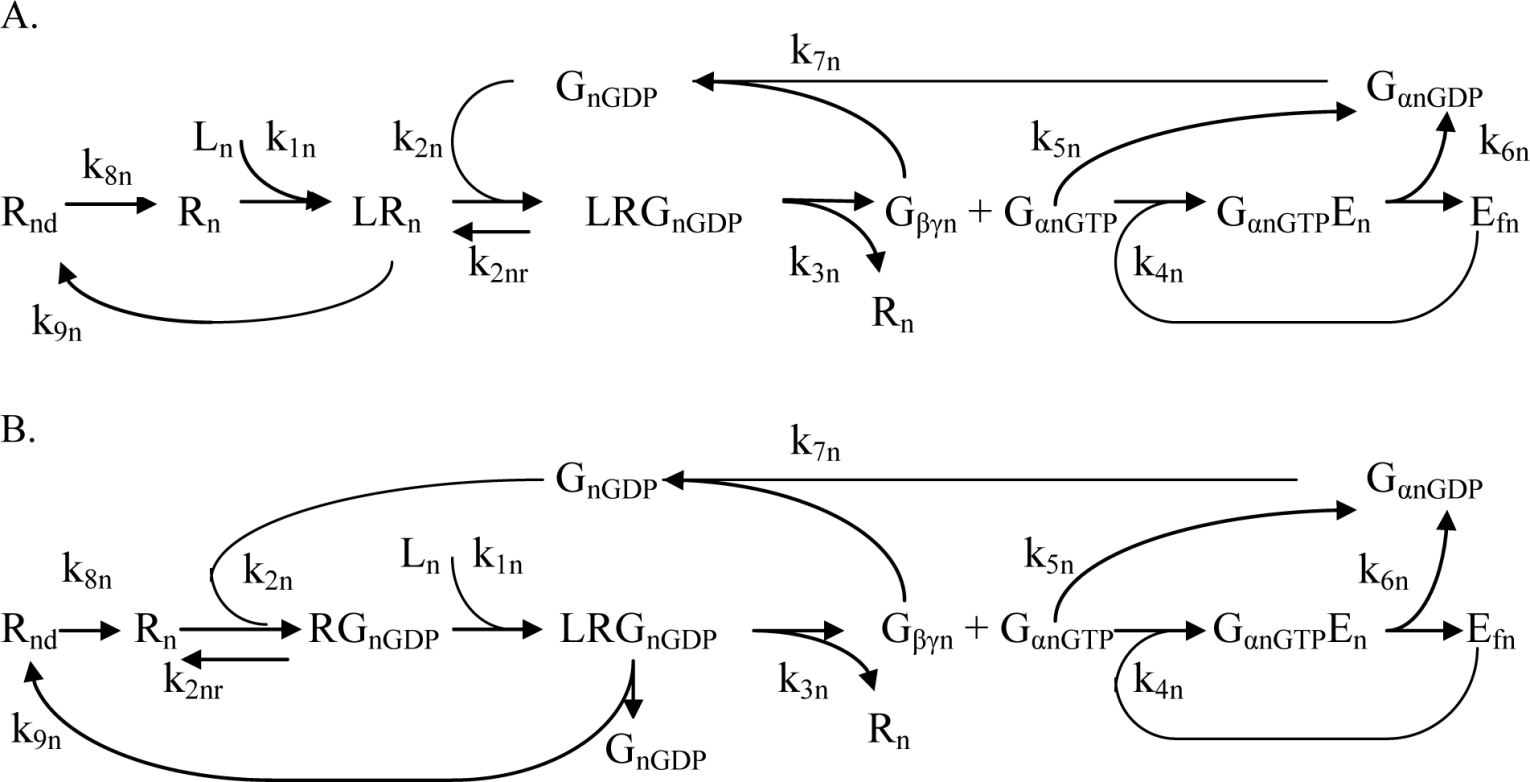
Diagram of the kinetic model of ligand, G protein and target enzyme interactions. R_nd_ is the desensitized receptor, R_n_ is the free receptor, L_n_ is the ligand, LR_n_ is the receptor bound with ligand, LRG_nGDP_ is the receptor bound with ligand and G protein, G_nGDP_ is the G proteins consisting of subunits α, β and γ. In this ground state the α-subunit is bound to GDP. G_αnGTP_ is the GTP-bound α subunit of G protein, G_βγn_ is the β and γ subunit of G protein, G_αnGDP_ is GDP-bound α subunit of G protein, G_αnGTP_E_n_ is the complex of the α-subunit carries the GTP and effectors enzyme (E_n_). E_fn_ is the enzyme that is not bound with G protein. The constants can be identified by their subcripts, where n the forward transition and nr is the reverse transition. For reversible reactions (double arrows), forward reactions are in the direction of association. (A) Collision coupling model. (B). Pre-coupling of receptor and G-protein model.

Plasma membrane receptors and G proteins usually possess a high number of distinct binding domains inducing the formation of large multiprotein signaling complexes. In quantitative models of GPCR signaling that incorporate these varied states, parameter values are often uncharacterized or varied over large ranges, making identification of important parameters and signaling outcomes difficult [53]. However, despite their diversity, signaling pathways in β-cells employ a set of common components that we have used for modeling. Fig 13 shows the reaction scheme for the minimal model of a receptor cascade. We modeled two different proposals about ligand-receptor interactions that were suggested in literature: 1. Collision coupling model, where a ligand binds to the free receptor and then the ligand-receptor complex ‘‘collides” with the free G-protein (Fig 13A). 2. Pre-coupling model where stable receptor/G-protein complex exists in the absence of ligand and ligand bounds with this complex (Fig 13B).

In the collision coupling model the initial steps in GPCR signaling take place in the plasma membrane and involve the binding of a ligand (L_n_) to a receptor that rapidly shifts an inactive receptor (R_n_) to an active state (L_n_R_n_). In the following steps the activated receptor couples to heterotrimeric G proteins (G_αβγnGDP_). In pre-coupling model the initial steps in GPCR signaling take place also in the plasma membrane. In this case a ligand binds with a receptor/G-protein complex.

The following steps are identical for both models. Heterotrimeric G proteins coupled with receptor activates through the release of a bound guanosine diphosphate (GDP) and the capture of guanosine triphosphate (GTP) on the G protein α-subunit. A series of intra and intermolecular events in the heterotrimer Gαβγ leads to a dissociation of this complex resulting in active Gα·GTP and Gβγ subunits.

Although both Gα·GTP and Gβγ can then activate different signaling pathways and effector proteins, we focus only on the reactions downstream of Gα·GTP activation since it is principal process for receptors, that we considerate in this article. In this case, as laterally diffusing Gα·GTP subunits bind isoforms of inactive (presumably freely diffusing) target enzymes (E_fn_) on the inner leaflet of the plasma membrane and forming a complex (G_αnGTP_ E_n_) (coefficient k_4n_), that activates or inhibits biochemical processes. Activated G proteins (G_αnGTP_) deactivate through their own GTPase activity (coefficient k_5n_) (or much faster when bound to E_fn_, coefficient k_6n_). This allows G_αnGDP_ to recombine with G_βγn_ (coefficient k_7n_) starting a new cycle.

Cells can fine-tune their excitability by changing the susceptibility of receptors, e.g. switch between active (L_n_R_n_−in collision coupling model or L_n_R_n_G_nGDP_ in pre-coupling model) forms and desensitized form (R_nd_) by regulating the number of receptors via internalization. We have modeled process of desensitization of activated receptor (rate constant—k_9n_). Internalized receptors can then undergo activation and return to the surface in our model (rate constant—k_8n_).

#### Receptor dynamics and G protein cascade

Our model of receptor dynamics is based on several earlier studies [209, 210]. However, we were not able to find a model that matches well to our aims. The majority of these models do not include a G-protein bound with target enzyme. For this reason we constructed a minimal model of the receptor signaling transduction and corresponding target enzymes activations.

Several suggestions were made. Our model assumes that initial concentration of ligand is sufficiently high so that it is not significantly depleted by binding to the receptors and therefore the ligands can be presumed as a fixed concentration. The process of receptors and G-proteins synthesis is assumed to compensate for intracellular receptor and G protein degradation, so that the total area density of receptors and G proteins in different states (free, active, and internalized) remains constant.

We have assumed that the processes occur in the plasma membrane and all the species, except for the agonist, are membrane bound and are diffuse freely. We simplify the model by ignoring the processes, such as the nucleotide exchange on G-proteins that are not bound to the receptor or bound to the receptor that is not bound to the ligand and others. Receptor desensitization was included as a process that can deactivate the receptor-ligand complex. The multiple deactivation and reactivation steps were shortened in single irreversible reactions (see Fig 13).

We modeled two different models of ligand-receptor interactions: 1. Collision coupling model and 2. Pre-coupling model (see Fig 13). Based on the above assumptions and using the law of mass action the collision coupling model suggests that ligands, receptors and G-proteins collide and binds to the corresponding enzyme transiently to produce target enzyme activation. For simplicity, we use concentrations on PM without brackets. Than a collision coupling model is represented as a system of ordinary differential equations:

$${\text{d LR}}_{\text{n}}\;/\;\text{dt}={\text{k}}_{1\text{n}}\;{\text{R}}_{\text{n}}\;([{\text{L}}_{\text{n}}]/({\text{K}}_{\text{L}}+([{\text{L}}_{\text{n}}]))+{\text{k}}_{2\text{nr}}\;{\text{LRG}}_{\text{n}}\;{\text{LRG}}_{2\text{n}}\;{\text{G}}_{\text{n}}*{\text{LR}}_{\text{n}}-{\text{LR}}_{9\text{n}}\;{\text{LR}}_{\text{n}}$$ (26)

$${\text{d LRG}}_{\text{n}}\;/\;\text{dt}={\text{k}}_{2\text{n}}\;{\text{G}}_{\text{n}}*{\text{LR}}_{\text{n}}-{\text{k}}_{2\text{nr}}\;{\text{LRG}}_{\text{n}}-{\text{k}}_{3\text{n}}\;{\text{LRG}}_{\text{n}}$$ (27)

$${\text{d R}}_{\text{nd}}\;/\;\text{dt}={\text{k}}_{9\text{n}}\;{\text{LR}}_{\text{n}}-{\text{k}}_{8\text{n}}\;{\text{R}}_{\text{nd}}$$ (28)

The following conservation constrain also holds (R_nt_)

$${\text{R}}_{\text{nt}}={\text{R}}_{\text{nd}}+{\text{R}}_{\text{n}}+{\text{LR}}_{\text{n}}+{\text{LRG}}_{\text{n}}$$ (29)

or

$${\text{R}}_{\text{n}}={\text{R}}_{\text{nt}}-{\text{R}}_{\text{nd}}-{\text{LR}}_{\text{n}}-{\text{LRG}}_{\text{n}}$$ (30)

where [L_n_] is the free ligand concentration; K_L_ is the Michaelis-Menten constant, R_n_, LR_n_, LRG_n_ and R_nd_ are, respectively, the area density of free, bound with ligand, bound with ligand and G-protein and internalized (desensitized) receptors and R_nt_ is the total receptor area density in # μm^-2^. (Definition L_n_R_n_G_n_ instead L_n_R_n_G_nGDP_ was used for simplification). k_1n_, k_2n_, k_3n_, k_8n_ and k_9n_ are the forward rate constants and k_2nr_ is the backward rate constant (see Fig 13A).

The first binding step reflects a simple bimolecular interaction between the ligand (L_n_) and the receptor (R_n_) (coefficient k_1n_) that is strictly dependent on ligand concentration. According to mass action kinetics, we have assumed that G_αnGTP_E_n_ are produced at a rate proportional to the area density of GTP-bound α subunit of G-protein, G_αnGTP_, and the inactive target protein area density (E_fn_*) (*coefficient k_4n_).

$${\text{d G}}_{\alpha \text{nGTP}}\;/\text{dt}={\text{k}}_{3\text{n}}\;{\text{LRG}}_{\text{n}}-{\text{k}}_{4\text{n}}\;\cdot {\text{G}}_{\alpha \text{nGTP}}*{\text{E}}_{\text{fn}}-{\text{k}}_{5\text{n}}\cdot {\text{G}}_{\alpha \text{nGTP}}$$ (31)

$${\text{d G}}_{\alpha \text{nGTP}}{\text{E}}_{\text{n}}/\text{dt}={\text{k}}_{4\text{n}}\;{\text{G}}_{\alpha \text{nGTP}}*{\text{E}}_{\text{fn}}-{\text{k}}_{6\text{n}}\;{\text{G}}_{\alpha \text{nGTP}}{\text{E}}_{\text{n}}$$ (32)

$${\text{d G}}_{\alpha \text{nGDP}}\;/\text{dt}={\text{k}}_{5\text{n}}\;{\text{G}}_{\alpha \text{nGTP}}+{\text{k}}_{6\text{n}}\;{\text{G}}_{\alpha \text{nGTP}}{\text{E}}_{\text{n}}-{\text{k}}_{7\text{n}}\;{\text{G}}_{\alpha \text{nGDP}}*{\text{G}}_{\beta \gamma \text{n}}$$ (33)

where k_4n_ - k_7n_ are the forward rate constants.

We suggest that the content of the endogenous G-proteins (G_nt_) and target enzymes (E_nt_) remain constant

$${\text{G}}_{\text{nt}}={\text{G}}_{\text{nGDT}}+{\text{LRG}}_{\text{n}}+{\text{G}}_{\alpha \text{nGTP}}+{\text{G}}_{\alpha \text{nGDP}}+{\text{G}}_{\alpha \text{nGTP}}{\text{E}}_{\text{n}}$$ (34)

or

$${\text{G}}_{\text{nGDP}}={\text{G}}_{\text{nt}}-{\text{L}}_{\text{n}}{\text{R}}_{\text{n}}{\text{G}}_{\text{n}}-{\text{G}}_{\alpha \text{nGTP}}-{\text{G}}_{\alpha \text{nGDP}}-{\text{G}}_{\alpha \text{nGTP}}{\text{E}}_{\text{n}}$$ (35)

$${\text{G}}_{\beta \gamma \text{n}}={\text{G}}_{\alpha \text{nGTP}}+{\text{G}}_{\alpha \text{nGDP}}+{\text{G}}_{\alpha \text{nGTP}}{\text{E}}_{\text{n}}$$ (36)

$${\text{E}}_{\text{nt}}={\text{E}}_{\text{fn}}+{\text{G}}_{\alpha \text{nGTP}}{\text{E}}_{\text{n}}$$ (37)

or

$${\text{E}}_{\text{fn}}={\text{E}}_{\text{nt}}-{\text{G}}_{\alpha \text{nGTP}}{\text{E}}_{\text{n}}$$ (38)

where G_nGDP_, LRG_n_, G_αnGTP_, G_αnGDP_ and G_αnGTP_E_n_ are the area density of free, bound with ligand and receptor, active, inactive and bound with the target enzyme G-proteins, respectively. G_nt_ is the total area density of G_n_ proteins per unit of PM, both activated and inactivated. E_fn_ is the free target enzyme and E_nt_ is the total area density of the target enzyme.

Pre-coupling mechanism of ligand-receptor interaction suggestes that the stable receptor/G-protein complex exists in the absence of ligand and ligand bounds with this complex (see Fig 13B). Equations for a pre-coupling model can also be obtained similarly Eqs 26–30 as a system of ordinary differential equations:

$${\text{d RG}}_{\text{n}}\;/\;\text{dt}={\text{k}}_{2\text{n}}\;{\text{R}}_{\text{n}}*{\text{G}}_{\text{n}}-{\text{k}}_{1\text{n}}\;([{\text{L}}_{\text{n}}]/({\text{K}}_{\text{L}}+([{\text{L}}_{\text{n}}]))\;{\text{LR}}_{\text{n}}-{\text{k}}_{2\text{nr}}\;{\text{R}}_{\text{n}}*{\text{G}}_{\text{n}}$$ (39)

$${\text{d LRG}}_{\text{n}}\;/\;\text{dt}={\text{k}}_{1\text{n}}\;[{\text{L}}_{\text{n}}]*{\text{RG}}_{\text{n}}-{\text{k}}_{3\text{n}}\;{\text{LRG}}_{\text{n}}-{\text{k}}_{9\text{n}}\;{\text{LRG}}_{\text{n}}$$ (40)

$${\text{d R}}_{\text{nd}}\;/\;\text{dt}={\text{k}}_{9\text{n}}\;{\text{LRG}}_{\text{n}}-{\text{k}}_{8\text{n}}\;{\text{R}}_{\text{nd}}$$ (41)

The following conservation constrains also hold

$${\text{R}}_{\text{nt}}={\text{R}}_{\text{nd}}+{\text{R}}_{\text{n}}+{\text{RG}}_{\text{n}}+{\text{LRG}}_{\text{n}}$$ (42)

$${\text{R}}_{\text{n}}={\text{R}}_{\text{nt}}-{\text{R}}_{\text{nd}}-{\text{RG}}_{\text{n}}-{\text{LRG}}_{\text{n}}$$ (43)

$${\text{G}}_{\text{nt}}={\text{G}}_{\text{nGDP}}+{\text{RG}}_{\text{n}}+{\text{LRG}}_{\text{n}}+{\text{G}}_{\alpha \text{nGTP}}+{\text{G}}_{\alpha \text{nGDP}}+{\text{G}}_{\alpha \text{nGTP}}{\text{E}}_{\text{n}}$$ (44)

or

$${\text{G}}_{\text{nGDP}}={\text{G}}_{\text{nt}}-{\text{RG}}_{\text{n}}-{\text{LRG}}_{\text{n}}-{\text{G}}_{\alpha \text{nGTP}}-{\text{G}}_{\alpha \text{nGDP}}-{\text{G}}_{\alpha \text{nGTP}}{\text{E}}_{\text{n}}$$ (45)

Other equations for pre-coupling model are similar to the collision coupling model (Eqs 31–38).

Several suggestions were accepted in order to determine of coefficients of both models:

The number of βγ subunits is the same as the number of α subunits, because each G-protein splits into one α and one βγ. Estimated ratio of G-protein to receptors was accepted as ~ 10:1 [211, 212].

Kinetics of ligand binding are usually fast relative to the other processes in the model (see [213]). The fast speed observed for receptor activation in response to saturated concentrations of small agonist molecules (coefficient k_1n_, Fig 13A) has half-life (t_1/2_) ranges between ≈ 40 and 100 ms in the case of α_2A_- and β_1_-adrenergic receptors, muscarinic M_1_- and M_2_-receptors [214] (k = ln2/t_1/2_). We used a coefficient of receptor ligand binding (k_1n_ ≈ 7 μM^-1^ s^-1^) for the collision coupled model that corresponds to the half-life about 100 ms.

Coefficient for binding of LR_n_ with target G protein in the collision coupling model (k_2n_ in Fig 13A) depends on the area density of moleculs on PM and was evaluated as 1 #^-1^μm^2^ s^-1^ [215]. Coefficient for receptor–G-protein unbinding (k_2nr_) was calculated for collision coupling model as 0.68 s^-1^ and for pre-coupling model as 6.8 s^-1^ [216].

Speed of G-protein activation (coefficient k_3n_ Fig 13A and 13B) is slow (half-life t_1/2_≈ 1 sec) [214]. This values corresponds k_3n_ ≈ 0.7 s^-1^. It is close to 0.8 s^-1^ evaluated by [217] or 1 s^-1^ [218].

Binding of G_αnGTP_ with target enzyme E_fn_ (coefficient k_4n_ in Fig 13A and 13B) was evaluated by [216] as 1 #^-1^μm^2^ s^-1^. Deactivation of G_αnGTP_ through its own GTPase activity (coefficient k_5n_ Fig 13A and 13B) was evaluated by [216] as 0.026 s^-1^. Dissociation of complex of G protein and target enzyme (coefficient k_6n_ Fig 13A and 13B) was evaluated by us as 0.4 s^-1^. The recombination of α and βγ subunits (coefficient k_7n_ in Fig 13A) is thought not to be limiting and was evaluated as 1 μm^2^ s^-1^ [216, 218]. Typical rate constants for desensitization of activated receptor (k_9n_) and its return to the surface (k_8n_) in our model was taken from model for slow GLP-1 receptor kinetics [24] (k_8n_ = 5 10^−5^ s^-1^ and k_9n_ = 0.000283 s^-1^). We used these parameters and coefficients if no additional experimental data can be found in literature for some receptors, corresponding G proteins or target enzymes.

Tonic receptor effects are simulated by including the presence of low concentrations of corresponding receptor ligands even when the experiments were simulated without specific agonists.

#### GLP-1R (R_1_)

We used the collision coupling model for this receptor. The processes were described similar to Eqs 26–40)

$$\text{d LR}1\;/\;\text{dt}={\text{R}}_{11}\;{\text{Re}}_{\text{GL}}\;(\text{GLP}1/({\text{K}}_{\text{GLP}1}+\text{GLP}1)+{\text{R}}_{12\text{r}}\;\text{LRG}1-{\text{R}}_{12}\;\text{G}1*\text{LR}1-{\text{R}}_{19}\;\text{LR}1$$ (46)

$$\text{d LRG}1\;/\;\text{dt}={\text{R}}_{12}\;\text{G}1*\text{LR}1-{\text{R}}_{12\text{r}}\;\text{LRG}1-{\text{R}}_{13}\;\text{LRG}1$$

$${\text{d Re}}_{\text{GLd}}\;/\;\text{dt}={\text{R}}_{19}\;\text{LR}1-{\text{R}}_{18}\;{\text{Re}}_{\text{GLd}}$$

$${\text{d G}}_{\text{aT}1}\;/\;\text{dt}={\text{R}}_{13}\;\text{LRG}1-{\text{R}}_{14}\;{\text{G}}_{\text{aT}1}*{\text{AC}}_{\text{pf}}-{\text{R}}_{15}\;{\text{G}}_{\text{aT}1}$$

$${\text{d AC}}_{\text{GLP}}\;/\;\text{dt}={\text{R}}_{14}\;{\text{G}}_{\text{aT}1}*{\text{AC}}_{\text{pf}}-{\text{R}}_{16}\;{\text{AC}}_{\text{GLP}}$$

$${\text{d G}}_{\text{aD}1}\;/\;\text{dt}={\text{R}}_{15}\;{\text{G}}_{\text{aT}1}+{\text{R}}_{16}\;{\text{AC}}_{\text{GLP}}-{\text{R}}_{17}\;{\text{G}}_{\text{aD}1}*{\text{G}}_{\text{bg}1}$$

$${\text{Re}}_{\text{GL}}={\text{Re}}_{\text{GLt}}-{\text{Re}}_{\text{GLd}}-\text{LR}1-\text{LRG}1$$

$${\text{G}}_{1}={\text{G}}_{1\text{t}}-{\text{LRG}}_{1}-{\text{G}}_{\text{aT}1}-{\text{G}}_{\text{aD}1}-{\text{AC}}_{\text{GLP}}$$

$${\text{G}}_{\text{bg}1}={\text{G}}_{\text{aT}1}+{\text{G}}_{\text{aD}1}+{\text{AC}}_{\text{GLP}}$$

GLP1=GLP1i+GLP11(t>t2)

GLP1 was used instead [L_n_], GLP1i is the initial GLP-1 concentration. GLP11 is the increase in GLP1 in time (t2), K_GLP1_ was used instead K_L_ and R_1X_ instead the corresponding coefficients (see Table 3). AC_GLP_ is the PM bound AC activated by GLP-1 receptor. The parameters and coefficients can be identified by their subscripts, where 1 was used instead n (Table 3).

Specific concentrations and coefficients: According [219] the amount of GLP-1R (Re_GLt_) in one INS-1 cell is about 2,000, i.e. it can be evaluated as about 2 # μm^-2^ (if cell surface is about 1000 μm^-2^). Half saturation (log_10_M = -10.5) for GLP1 was measured as an increase in cAMP production in the pancreatic β-cell line (Fig 5 from [220]), that we used for evaluation of K_GLP1_ (0.000031 μM, Table 3). Other coefficients were chosen as general coefficients.

#### GIPR (R_2_)

Regulated mechanisms for GIP receptor are assumed to be similar to those of GLP-1R.

$$\text{d LR}2\;/\;\text{dt}={\text{R}}_{21}\;{\text{R}}_{\text{eGI}}\;\text{GIP}/({\text{K}}_{\text{GIP}}+\text{GIP})+{\text{R}}_{22\text{r}}\;\text{LRG}2-{\text{R}}_{22}\;\text{G}2*\text{LR}2-{\text{R}}_{29}\;\text{LR}2$$ (47)

$$\text{d LRG}2\;/\;\text{dt}={\text{R}}_{22}\;\text{G}2*\text{LR}2-{\text{R}}_{22\text{r}}\;\text{LRG}2-{\text{R}}_{23}\;\text{LRG}2$$

$${\text{d Re}}_{\text{GId}}\;/\;\text{dt}={\text{R}}_{29}\cdot \text{LR}2-{\text{R}}_{28}\cdot {\text{Re}}_{\text{GId}}$$

$${\text{d G}}_{\alpha \text{T}2}\;/\;\text{dt}={\text{R}}_{23}\;{\text{LRG}}_{2}-{\text{R}}_{24}\;{\text{G}}_{\alpha \text{T}2}*{\text{AC}}_{\text{pf}}-{\text{R}}_{25}\;{\text{G}}_{\alpha \text{T}2}$$

$${\text{d AC}}_{\text{GIP}}\;/\;\text{dt}={\text{R}}_{24}\;{\text{G}}_{\alpha \text{T}2}*{\text{AC}}_{\text{pf}}-{\text{R}}_{26}\;{\text{AC}}_{\text{GIP}}$$

$${\text{d G}}_{\alpha \text{D}2}\;/\;\text{dt}={\text{R}}_{25}\;{\text{G}}_{\alpha \text{T}2}+{\text{R}}_{26}\;{\text{AC}}_{\text{GIP}}-{\text{R}}_{27}\;{\text{G}}_{\alpha \text{D}2}*{\text{G}}_{\text{bg}2}$$

$${\text{Re}}_{\text{GI}}={\text{Re}}_{\text{GIt}}-{\text{Re}}_{\text{GId}}-{\text{LR}}_{2}-{\text{LRG}}_{2}$$

$$\text{G}2={\text{G}}_{2\text{t}}-{\text{LRG}}_{2}-{\text{G}}_{\text{aT}2}-{\text{G}}_{\text{aD}2}-{\text{AC}}_{\text{GIP}}$$

$${\text{G}}_{\text{bg}2}={\text{G}}_{\text{aT}2}+{\text{G}}_{\text{aD}2}+{\text{AC}}_{\text{GIP}}$$

$$\text{GIP}={\text{GIP}}_{\text{i}}+\text{GIP}1\;(\text{t}>\text{t}3)$$

GIP was used instead [L_n_], K_GIP_ instead K_L_ and R_2X_ instead the corresponding coefficients (see Table 3). AC_GIP_ is the PM bound AC_p_ activated by GIP receptor. GIP_i_ is the initial GIP concentration. GIP1 is the increase in GIP in time (t3). The parameters and coefficients can be identified by their subscripts, where 2 was used instead n (Table 3).

Specific concentrations and coefficients: INS-1 cells expressed an average of 2443μ400 GIP receptors (G_2t_) on the cell surface or ~ 2.5 # μm^-2^ [221]. EC_50_ for GIP was evaluated as 171 pM for activation cAMP production [222], i.e. K_GIP_ = 0.000171 μM. Other coefficients were chosen as the general coefficients.

#### α2A-adrenergic receptor (R3)

A model of α_2A_ adrenergic receptor activation in β-cell was constructed as a collision coupling model. In this case an active G_α_GTP messenger (G_αT3_) released from stimulated receptor binds to the enzyme AC_pf_.

$$\text{d LR}3\;/\;\text{dt}={\text{R}}_{31}\;{\text{Re}}_{\text{AR}}\;{\text{AR}}_{3}/({\text{K}}_{\text{AR}3}+{\text{AR}}_{3})+{\text{R}}_{32\text{r}}\;\text{LRG}3-{\text{R}}_{32}\;{\text{G}}_{3}*{\text{LR}}_{3}-{\text{R}}_{39}\;{\text{LR}}_{3}$$ (48)

$$\text{d LRG}3\;/\;\text{dt}={\text{R}}_{32}\;\text{G}3*\text{LR}3-{\text{R}}_{32\text{r}}\;\text{LRG}3-{\text{R}}_{33}\;\text{LRG}3$$

$${\text{d Re}}_{\text{ARd}}\;/\;\text{dt}={\text{R}}_{39}\cdot \text{LR}3-{\text{R}}_{38}\cdot {\text{Re}}_{\text{ARd}}$$

$${\text{d G}}_{\alpha \text{T}3}\;/\;\text{dt}={\text{R}}_{33}\;{\text{LRG}}_{3}-{\text{R}}_{34}\;{\text{G}}_{\alpha \text{T}3}*{\text{AC}}_{\text{pf}}-{\text{R}}_{35}\;{\text{G}}_{\alpha \text{T}3}$$

$${\text{d AC}}_{\text{AR}}\;/\text{dt}={\text{R}}_{34}\;{\text{G}}_{\alpha \text{T}3}*{\text{AC}}_{\text{pf}}-{\text{R}}_{36}\;{\text{AC}}_{\text{AR}}$$

$${\text{d G}}_{\alpha \text{D}3}\;/\text{dt}={\text{R}}_{35}\;{\text{G}}_{\alpha \text{T}3}+{\text{R}}_{36}\;{\text{AC}}_{\text{AR}}-{\text{R}}_{37}\;{\text{G}}_{\alpha \text{D}3}\;{\text{G}}_{\text{bg}3}$$

$${\text{Re}}_{\text{AR}}={\text{Re}}_{\text{ARt}}-{\text{Re}}_{\text{ARd}}-{\text{LR}}_{3}-{\text{LRG}}_{3}$$

$${\text{G}}_{3}={\text{G}}_{3\text{t}}-{\text{LRG}}_{3}-{\text{G}}_{\text{aT}3}-{\text{G}}_{\text{aD}3}-{\text{AC}}_{\text{AR}}$$

$${\text{G}}_{\text{bg}3}={\text{G}}_{\text{aT}3}+{\text{G}}_{\text{aD}3}+{\text{AC}}_{\text{AR}}$$

$${\text{AR}}_{3}={\text{AR}}_{3\text{i}}+{\text{AR}}_{3}1\;(\text{t}>\text{t}4)$$

AR_3_ was used instead [L_n_], K_AR3_ instead K_L_ and R_3X_ instead the corresponding coefficients (see Table 3). AR_3i_ is the initial AR_3_ concentration. AR_31_ is the increase in AR_3_ in time (t4), AC_AR_ is the PM bound AC activated by α_2A_-adrenergic receptor. The parameters and coefficients can be identified by their subscripts, where 3 was used instead n (Table 3).

Specific concentrations and coefficients: Noradrenaline (norepinephrine) was shown to be a potent inhibitor of GSIS from rat pancreatic islets, with half-maximal inhibition of the secretory response to 20 mM-glucose occurring at approx. 0.3 μM [223], and we accepted this value for the coefficient K_AR3_. Other coefficients were chosen as the general coefficients.

Basal concentrations of GLP-1R, GIPR and AdR, that determinate constitutive receptor activity, were taken in such manner that 0.1 AC_P_ was bound for each receptor (see Eq 59).

#### M3 muscarinic receptor (R_6_)

We used the pre-coupled model for this receptor.

$${\text{d RG}}_{6}\;/\;\text{dt}={\text{R}}_{62}\;{\text{Re}}_{\text{M}3}*{\text{G}}_{6}-{\text{R}}_{61}\;{\text{RG}}_{6}\;{\text{AM}}_{3}/\;({\text{K}}_{\text{AM}3}+{\text{AM}}_{3})-{\text{R}}_{62\text{r}}\;{\text{RG}}_{6}$$ (49)

$${\text{d LRG}}_{6}\;/\;\text{dt}={\text{R}}_{61}\;{\text{RG}}_{6}\;{\text{AM}}_{3}/\;({\text{K}}_{\text{AM}3}+{\text{AM}}_{3})-{\text{R}}_{63}\;{\text{LRG}}_{6}-{\text{R}}_{69}\;{\text{LRG}}_{6}$$

$${\text{d Re}}_{\text{M}3\text{d}}\;/\;\text{dt}={\text{R}}_{69}\cdot {\text{LRG}}_{6}-{\text{R}}_{68}\cdot {\text{Re}}_{\text{M}3\text{d}}$$

$${\text{d G}}_{\alpha \text{T}6}\;/\;\text{dt}={\text{R}}_{63}\;{\text{LRG}}_{6}-{\text{R}}_{64}\;{\text{G}}_{\alpha \text{T}6}*{\text{PLC}}_{\text{pf}}-{\text{R}}_{65}\;{\text{G}}_{\alpha \text{T}6}$$

$${\text{d PLC}}_{\text{M}3}\;/\text{dt}={\text{R}}_{64}\;{\text{G}}_{\alpha \text{T}6}*\;{\text{PLC}}_{\text{pf}}-{\text{R}}_{66}\;{\text{PLC}}_{\text{M}3}$$

$${\text{d G}}_{\alpha \text{D}6}\;/\text{dt}={\text{R}}_{65}\;{\text{G}}_{\alpha \text{T}6}+{\text{R}}_{66}\;{\text{PLC}}_{\text{M}3}-{\text{R}}_{67}\;{\text{G}}_{\alpha \text{D}6}*{\text{G}}_{\text{bg}6}$$

$${\text{Re}}_{\text{M}3}={\text{Re}}_{\text{M}3\text{t}}-{\text{Re}}_{\text{M}3\text{d}}-{\text{RG}}_{6}-{\text{LRG}}_{6}$$

$${\text{G}}_{6}={\text{G}}_{6\text{t}}-\text{RG}6-\text{LRG}6-{\text{G}}_{\text{aT}6}-{\text{G}}_{\text{aD}6}-{\text{PLC}}_{\text{M}3}$$

$${\text{G}}_{\text{bg}6}={\text{G}}_{\text{aT}6}+{\text{G}}_{\text{aD}6}+{\text{PLC}}_{\text{M}3}$$

$${\text{AM}}_{3}={\text{AM}}_{3\text{i}}+{\text{AM}}_{31}\;(\text{t}>\text{t}6)$$

AM_3_ was used instead [L_n_], K_AM3_ instead K_L_ and R_6X_ instead the corresponding coefficients (see Table 4). PLC_M3_ is the PM bound PLC activated by M_3_ muscarinic receptor; PLC_pf_ is the free PLC on PM (see Eq 88). AM_3i_ is the initial AM_3_ concentration. AM_31_ is the increase in AM_3_ in time (t6). The parameters and coefficients can be identified by their subscripts, where 6 or M_3_ were used instead n.

Specific concentrations and coefficients: acetylcholine and carbachol (MR agonist) have different K_AM3_. We used the coefficients by Hoffman et al [224] for acetylcholine in human M_3_ muscarinic receptor (see Table 4). Agonists such as acetylcholine, carbachol or muscarine activate each receptor construct with half-maximal activation times between 60 and 70ms for human M3 muscarinic receptor [225] (coefficient R_61_) (or R_61_ = 10 s^-1^).

Receptor deactivation kinetics was found to be slow and independent of agonist concentrations [224] (coefficient R_62r_ was taken according [216]). R_68_ (coef k_8n_ in Fig 13B) was evaluated as the coefficient of M_3_ receptor dephosphorylation in work [226]. Human M_3_ receptors were internalized with a half life of 11 min (660 s) for M_3_ receptors expressed in COS-7 cells and in the presence of 10^−3^ M carbamylcholine (for receptor activation) [227] and we determined the coefficient R_69_ from these data. Other coefficients were chosen as general coefficients (Table 4).

#### FFAR1/GPR40 (R_7_)

A model for FFAR1/GPR40 was made as for collision coupling model receptors.

$$\text{d LR}7\;/\;\text{dt}={\text{R}}_{71}\;{\text{Re}}_{\text{R}40}\;{\text{AR}}_{7}/\;({\text{K}}_{\text{AR}7}+{\text{AR}}_{7})+{\text{R}}_{72\text{r}}\;{\text{LRG}}_{7}-{\text{R}}_{72}\;\text{G}7*{\text{LR}}_{7}-{\text{R}}_{79}\;{\text{LR}}_{7}$$ (50)

$$\text{d LRG}7\;/\;\text{dt}={\text{R}}_{72}\;{\text{G}}_{7}*{\text{LR}}_{7}\;{\text{R}}_{72\text{r}}\;{\text{LRG}}_{7}-{\text{R}}_{73}\;{\text{LRG}}_{7}$$

$${\text{d Re}}_{\text{R}40\text{d}}\;/\;\text{dt}={\text{R}}_{79}\cdot {\text{LR}}_{7}-{\text{R}}_{78}\cdot {\text{Re}}_{\text{R}40\text{d}}$$

$${\text{d G}}_{\alpha \text{T}7}\;/\;\text{dt}={\text{R}}_{73}\;{\text{LRG}}_{7}-{\text{R}}_{74}\;{\text{G}}_{\alpha \text{T}7}*{\text{PLC}}_{\text{Pf}}-{\text{R}}_{75}\;{\text{G}}_{\alpha \text{T}7}$$

$${\text{d PLC}}_{\text{R}40}\;/\;\text{dt}={\text{R}}_{74}\;{\text{G}}_{\alpha \text{T}7}*\;{\text{PLC}}_{\text{pf}}-{\text{R}}_{76}\;{\text{PLC}}_{\text{R}40}$$

$${\text{d G}}_{\alpha \text{D}7}\;/\text{dt}={\text{R}}_{75}\;{\text{G}}_{\alpha \text{T}7}+{\text{R}}_{76}\;{\text{PLC}}_{\text{R}40}-{\text{R}}_{77}\;{\text{G}}_{\alpha \text{D}7}*{\text{G}}_{\text{bg}7}$$

$${\text{Re}}_{\text{R}40}={\text{Re}}_{\text{R}40\text{t}}-{\text{Re}}_{\text{R}40\text{d}}-{\text{LRG}}_{7}-{\text{LR}}_{7}$$

$${\text{G}}_{7}={\text{G}}_{7\text{t}}-{\text{LRG}}_{7}-{\text{G}}_{\text{aT}7}-{\text{G}}_{\text{aD}7}-{\text{PLC}}_{\text{R}40}$$

$${\text{G}}_{\text{bg}7}={\text{G}}_{\text{aT}7}+{\text{G}}_{\text{aD}7}+{\text{PLC}}_{\text{R}40}$$

$${\text{AR}}_{7}={\text{AR}}_{7\text{i}}+{\text{AR}}_{71}\;(\text{t}>\text{t}7)$$

AR_7_ was used instead [L_n_], K_AR7_ instead K_L_ and R_7X_ instead the corresponding coefficients (see Table 4). PLC_R40_ is the PM bound PLC activated by FFAR1/GPR40 receptor. AR_7i_ is the initial AR_7_ concentration. AR_71_ is the increase in AR_7_ in time (t7). The parameters and coefficients can be identified by their subscripts, where 7 or R40 were used instead of n.

Specific concentrations and coefficients: K_AR7_ for AR7 was evaluated as palmitic acid potency (EC_50_) to induce [Ca^2+^]_c_ rise in mouse CHO cells expressing FFAR1/GPR40 [167]. Other coefficients were chosen as the general coefficients.

Basal concentrations of FFAR1/GPR40 and AR_7_, that determinate constitutive receptor activity, were taken in such manner that 0.1 PLC_Pt_ was bound for each receptor (see Eq 79).

### Calmodulin

Ca^2+^ binds to calmodulin (CaM) in four steps and generates four species of Ca^2+^-bound calmodulin: CaCaM, Ca_2_CaM, Ca_3_CaM, and Ca_4_CaM. However, CaM needs to bind at least 3 Ca^2+^ to be active. We accepted the model for CaM dynamic from [23] (Table 5).

$$\text{dCaCaM}/\text{dt}={\text{k}}_{1\text{f}}\;{\left[{\text{Ca}}^{2+}\right]}_{\text{c}}\;\text{CAM}-{\text{k}}_{1\text{b}}\;\text{CaCaM}$$ (51)

where

$${\text{Ca}}_{2}\text{CaM}=({\text{k}}_{2\text{f}}/{\text{k}}_{2\text{b}})\;{\left[{\text{Ca}}^{2+}\right]}_{\text{c}}\;\text{CaCaM}$$ (52)

$${\text{Ca}}_{3}\text{CaM}=({\text{k}}_{3\text{f}}/{\text{k}}_{3\text{b}})\;{\left[{\text{Ca}}^{2+}\right]}_{\text{c}}\;{\text{Ca}}_{2}\text{CaM}$$ (53)

$${\text{Ca}}_{4}\text{CaM}=({\text{k}}_{4\text{f}}/{\text{k}}_{4\text{b}})\;{\left[{\text{Ca}}^{2+}\right]}_{\text{c}}\;{\text{Ca}}_{3}\text{CaM}$$ (54)

$$\text{CaM}={\text{CaM}}_{\text{o}}\;-\;\text{CaCaM}\;-\;{\text{Ca}}_{2}\text{CaM}\;-\;{\text{Ca}}_{3}\text{CaM}\;-\;{\text{Ca}}_{4}\text{CaM}$$ (55)

$${\text{CaM}}_{\text{a}}={\text{Ca}}_{3}\text{CaM}+{\text{Ca}}_{4}\text{CaM}$$ (56)

where k_1f_–k_4f_ are the forward rate constants and k_1b_–k_4b_ are the backward rate constants in the four steps of Ca^2+^ binding to CaM, CaM_o_ is the total amount calmodulin, CaM_a_ is the active form of CaM.

### Modeling of cAMP pathway

Activated adenylyl cyclase synthesizes cAMP from the substrate Mg^2+^ATP. Our recent mathematical model of the cAMP pathway in pancreatic β-cells includes detailed descriptions of interactions between [Ca^2+^]_c_, Ca^2+^-bound calmodulin (Ca^2+^/CaM), adenylyl cyclase (AC), phosphodiesterase (PDE) and dynamics of cAMP concentration in the cytoplasm [23]. We have extended this model to include a description of cAMP-dependent modulation of PKA and Ecap and an activation of exocytosis processes [25]. In this article we added the equation for cAMP production by soluble Ca^2+^-activated AC isoform. As cAMP molecules are known to diffuse through the cytosol, we refer to cAMP production and degradation in terms of concentration (in μM) instead of the area density of molecules on PM. The dynamic of intracellular concentration of cAMP is determined by the rates of cAMP synthesis and degradationcan (see [23]):

$$\text{d cAMP}\;/\text{dt}={\text{V}}_{\text{AC}}\;-\;{\text{V}}_{\text{PDE}}\;-\;{\text{k}}_{\text{da}}\;\text{cAMP}$$ (57)

where V_AC_ is AC activity, V_PDE_ is the PDE activity, k_da_ is the coefficient of PDE-independent cAMP degradation.

AC activities were divided into two functionally distinct categories:

$${\text{V}}_{\text{AC}}={\text{V}}_{\text{ACp}}+{\text{V}}_{\text{ACc}}$$ (58)

where V_ACp_ is G-protein and Ca^2+^ dependent AC activity on PM, V_ACc_ is the glucose and Ca^2+^-activated cytoplasmic AC activity that is independent on G-proteins.

GLP-1 and GIP activate and catecholamines inhibite the same CaM and Ca^2+^-dependent isoform of AC on PM that bound corresponding G protens for activation. Equation for the area density of this activated AC isoform on PM is based on our previous analysis (see Eqs 46–48)

$${\text{AC}}_{\text{pt}}={\text{AC}}_{\text{GLP}}+{\text{AC}}_{\text{GIP}}+{\text{AC}}_{\text{AR}}+{\text{AC}}_{\text{pf}}$$ (59)

or

$${\text{AC}}_{\text{pf}}={\text{AC}}_{\text{pt}}-{\text{AC}}_{\text{GLP}}-{\text{AC}}_{\text{GIP}}-{\text{AC}}_{\text{AR}}$$ (60)

where

$${\text{AC}}_{\text{pa}}={\text{AC}}_{\text{GLP}}+{\text{AC}}_{\text{GIP}}$$ (61)

$${\text{AC}}_{\text{par}}={\text{AC}}_{\text{pa}}/{\text{AC}}_{\text{pt}}$$ (62)

AC_pt_ is the total concentration of AC isoform on PM, AC_pf_ is the concentration of free AC isoform on PM, AC_GLP_, AC_GIP_ and AC_AR_ are the concentrations of AC_p_ bound with corresponding G-proteins activated by GLP-1R, GIPR and α_2A_ adrenergic receptors (see above). AC_pa_ is the area density of active AC molecules that can be stimulated by G-proteins, AC_par_ is the relative concentration of AC_pa_. CaM/Ca^2+^ dependence of AC_p_ activity was accepted similarly to work [23],

$${\text{V}}_{\text{ACp}}={\text{V}}_{\text{mCaM}}{\text{f}}_{\text{acca}}{\text{AC}}_{\text{par}}$$ (63)

where

$${\text{f}}_{\text{acca}}=\frac{{\text{CaM}}_{\text{a}}}{{\text{CaM}}_{\text{a}}+{\text{K}}_{\text{PCaM}}}\;\frac{{\text{K}}_{\text{NCa}}}{{\text{K}}_{\text{NCa}}+{\left[{\text{Ca}}^{2+}\right]}_{\text{c}}}$$ (64)

V_mCaM_ is the maximum AC_p_ activity, f_acca_ is CaM activation factor, K_PCaM_ is the CaM_a_ activation constant, K_NCa_ is the [Ca^2+^]_c_ inhibition constant.

It was found that in S49 lymphoma cells, a widely used model system, each cell contains only a few thousand functional copies of the catalytic subunit of AC for binding forskolin (3000 molecules per cell) that can be consider as evaluation of PM bound AC (as 3 # μm^-2^) and G-protein appears to exist in stoichiometric excess relative to AC [228]. We used this data for evaluation of the content of PM bound AC isoform in β cell (Table 5).

Another isoform is cytoplasmic AC (AC_c_) that can be activated by ATP and Ca^2+^. We used a the Michaelis-Menten function for an dependence AC_c_ on ATP and [Ca^2+^]_c_ concentrations that were found for this AC isoform [229]

$${\text{V}}_{\text{ACS}}=\frac{{\text{V}}_{\text{mACc}}\;\text{ATP}}{\text{ATP}+{\text{K}}_{\text{mAACS}}}\;\frac{{\left[{\text{Ca}}^{2+}\right]}_{\text{c}}}{{\left[{\text{Ca}}^{2+}\right]}_{\text{c}}+{\text{K}}_{\text{mCACS}}}+\;{\text{k}}_{\text{ACS}}$$ (65)

where V_mACc_ is the maximum AC_c_ activity, K_mAACS_ is the of ATP activation constant, K_mCACS_ is the [Ca^2+^]_c_ activation constant, k_ACS_ is the coefficient of the constitutive AC_c_ activity.

The equation for the function of PDE was accepted from [23] in simplificated form. We added also the coefficient (k_ipde_) for a simulation of the specific inhibition.

$${\text{V}}_{\text{pde}}={\text{k}}_{\text{ipde}}\;(\;{\text{V}}_{\text{gpde}}+{\text{V}}_{\text{cpde}}\frac{{\text{CaM}}_{\text{a}}}{{\text{CaM}}_{\text{a}}+{\text{K}}_{\text{dpe}}})\;\frac{\text{cAMP}}{\text{cAMP}+{\text{K}}_{\text{pde}}}$$ (66)

$${\text{k}}_{\text{ipde}}={\text{k}}_{\text{ipdei}}+{\text{k}}_{\text{ipde}1}\;(\text{t}>{\text{t}}_{\text{pde}})$$

where V_gpde_ is the activity of Ca^2+^/CaM-independent PDE, V_cpde_ is the basal level of Ca^2+^/CaM dependent PDE activity, K_dpe_ is the CaM_a_ activation constant, K_pde_ is the cAMP activation constant, k_ipde_ is the the activation or inhibition coefficient. k_ipdei_ is the basal coefficient. k_ipde1_ is the change in k_ipde_ in time (t_pde_).

#### cAMP dependent PKA and Epac activation

Binding of cAMP to the regulatory units of PKA or Epac results in release of the catalytic units and an activation of PKA or Epac. We used the mathematical models of PKA activation [198]. Relative steady-state level of PKA activation by cAMP (f_pca_) was described by an empirical Hill-type equation [25].

$$\text{d PKAa}\;/\;\text{dt}={\text{k}}_{\text{ak}}\;({\text{f}}_{\text{pca}}\;{\text{PKA}}_{\text{i}}\;-\;{\text{PKA}}_{\text{a}})/{\text{t}}_{\text{pka}}\;,$$ (67)

where

PKAi=1−PKAa, (68)

$${\text{f}}_{\text{pca}}={\text{cAMP}}^{\text{hpca}}/({{\text{K}}_{\text{pcm}}}^{\text{hpca}}+{\text{cAMP}}^{\text{hpca}})$$ (69)

where PKA_a_ is the relative concentration of active PKA, PKA_i_ is the relative concentration of inactive PKA, the total relative concentration of active and inactive PKA was accepted as 1, t_pka_ is the time constant; k_ak_ is the scaling factor, f_pca_ is cAMP potential factor, where K_pcm_ is the cAMP activation coefficient [230] and hpca is the Hill coefficient.

The pathway, that includes Epac activation, was modeled similarly to the PKA dynamics

$${\text{d EP}}_{\text{a}}/\;\text{dt}=({\text{f}}_{\text{EcA}}\;{\text{EP}}_{\text{i}}\;-\;{\text{EP}}_{\text{a}})/\;{\text{t}}_{\text{ep}}$$ (70)

where

$${\text{EP}}_{\text{i}}=1\;-\;{\text{EP}}_{\text{a}};$$ (71)

$${\text{f}}_{\text{EcA}}={\text{cAMP}}^{\;\text{hce}}/({\text{K}}_{\text{mep}}{}^{\text{hce}}+{\text{cAMP}}^{\text{hce}})$$ (72)

where EP_a_ is the relative concentration of active Epac, EP_i_ is the relative concentration of inactive Epac, the total relative concentration of active and inactive Epac was accepted as 1; f_EcA_ is the cAMP potential factor, where K_mep_ is the cAMP activation constant for Epac, hce is the Hill coefficient, t_ep_ is the time constant.

Epac has a lower binding capacity for cAMP to compare with PKA [94] (see Table 5).

### Phosphoinositides

Phosphoinositides regulate numerous processes in pancreatic β-cells. We modeled phosphoinositides dynamics using the models and coefficients for sA201 cells [200, 201, 231] because a few quantitative kinetic measurements were made for pancreatic β-cells. According to [96] P4P synthesis activates by PKCa and Ca^2+^ that we have also taken into account introducing the specific term (f_PI_). Than the equation for P4P dynamic can be written as:

$$\text{d P}4\text{P}\;/\;\text{dt}\;={\text{k}}_{\text{PI}}\;{\text{f}}_{\text{PI}}\;\text{PI}+{\text{k}}_{\text{P}4\text{Pr}}\;{\text{PIP}}_{2}-{\text{k}}_{\text{PIr}}\;\text{P}4\text{P}-{\text{k}}_{\text{P}4\text{P}}\;\text{P}4\text{P}$$ (73)

where

$${\text{f}}_{\text{PI}}\;=\;\frac{{\left[{\text{Ca}}^{2+}\right]}_{\text{c}}{}^{2}}{{\left[{\text{Ca}}^{2+}\right]}_{\text{c}}{}^{2}\;+{\text{K}}_{\text{CaPI}}{}^{2}}\;\frac{{\text{PKC}}_{\text{a}}{}^{2}}{{\text{PKC}}_{\text{a}}{}^{2}+{\text{K}}_{\text{P}4\text{PK}}{}^{2}}+{\text{k}}_{\text{cpi}}$$ (74)

where PI is the intracellular pool of phospholipids on PM, PIP_2_ is the concentration of plasma membrane-bound phosphatidylinositol bisphosphate, k_PI_ is the P4P production coefficient, k_PIr_ is the backward rate constant for P4P, k_P4Pr_ is the backward rate constant for PIP_2_, k_P4P_ is the the PIP_2_ production coefficient, K_CaPI_ is the [Ca^2+^]_c_ activation coefficient, K_P4PK_ is the PKC_a_ activation coefficient.

#### PIP_2_ dynamics

According to [183] ATP dose-dependently stimulated PIP_2_ synthesis has the half-maximally stimulation at 300 μM ATP that significantly lower than ATP concentration in cytoplasm (see [31]). For this reason we did not take into account ATP concentration dependence at PIP_2_ synthesis. On the other hand PLC hydrolyzes plasma PIP_2_ molecules into inositol trisphosphate (IP_3_) and DAG. The rate of this reaction depends on activity of PLC.

$${\text{d PIP}}_{2}\;/\;\text{dt}\;={\text{k}}_{\text{P}4\text{P}}\;\text{P}4\text{P}-{\text{f}}_{\text{PIP}2}\;{\text{V}}_{\text{PL}}-{\text{k}}_{\text{P}4\text{Pr}}\;{\text{PIP}}_{2}$$ (75)

where

$${\text{f}}_{\text{PIP}2}={\text{PIP}}_{2}/({\text{K}}_{\text{PIP}2}+{\text{PIP}}_{2})$$ (76)

where V_PL_ is the PLC activity (see below, Eq 77), K_PIP2_ is the PIP_2_ activation coefficient.

Density of phosphoinositides at the plasma membrane in β-cell remains uncertain. We assumed that free PIP_2_ is about 5000 # μM^-2^ in our modeling similarly evaluation for tsA201 cells in work [201].

### PLC signaling pathway

Activation of PLC by M3 muscarinic receptors and FFAR1/GPR40 receptors was modeled above (Eqs 49–50). However, PLC activity is also regulated by G-protein independent (but [Ca^2+^]_c_ dependent) ways in insulin-secreting cells. We consider two different PLC isoforms: 1. MR and FFAR1/GPR40 activated (PLC_p_). 2. Ca^2+^ activated G-protein independent PLC form (PLC_c_). A schematic of these two PLC isoforms is shown in Fig 1.

$${\text{V}}_{\text{PL}}={\text{V}}_{\text{PLP}}+{\text{V}}_{\text{PLC}}+{\text{k}}_{\text{pPL}}$$ (77)

where

$${\text{V}}_{\text{PLP}}={\text{V}}_{\text{mPLP}}\frac{{\text{PLC}}_{\text{pa}}}{{\text{PLC}}_{\text{pt}}}\;\frac{{\left[{\text{Ca}}^{2+}\right]}_{\text{c}}{}^{2}}{{\text{K}}_{\text{CaPL}}{}^{2}+{\left[{\text{Ca}}^{2+}\right]}_{\text{c}}{}^{2}}$$ (78)

$$\begin{array}{c} {\text{V}}_{\text{mPLP}}={\text{V}}_{\text{mPLPi}}+{\text{V}}_{\text{mPLP}1}\;(\text{t}>\text{t}10)\\ {\text{PLC}}_{\text{pa}}={\text{PLC}}_{\text{M}3}+{\text{PLC}}_{\text{R}40} \end{array}$$ (79)

$${\text{PLC}}_{\text{pf}}={\text{PLC}}_{\text{pt}}\;-\;{\text{PLC}}_{\text{pa}}$$ (80)

$${\text{V}}_{\text{PLC}}={\text{V}}_{\text{mPLC}}\;{\left[{\text{Ca}}^{2+}\right]}_{\text{c}}{}^{2}/({\text{K}}_{\text{CCaPL}}{}^{2}+{\left[{\text{Ca}}^{2+}\right]}_{\text{c}}{}^{2}\;)$$ (81)

where V_PL_ is the total PLC activity, V_PLP_ is the G-protein dependent and V_PLC_ is the G-protein independent (but Ca^2+^ dependent) PLC activity, k_pPL_ is the coefficient of the PLC constitutive activity. V_mPLP_ is the maximum of V_PLP_ activity, K_CaPL_ is the [Ca^2+^]_c_ activation constant, V_mPLPi_ is the initial (basal) activity. V_mPLP1_ is the increase in V_mPLC_ in time (t10), PLC_pt_ is the is the total concentration of PLC_p_ isoform on PM, PLC_pa_ is the concentration of PLC_p_ bound with MR and FFAR1/GPR40 receptors, PLC_pf_ is the concentration of free PLC_p_, V_mPLC_ is the the maximum V_PLC_ activity, K_CCaPL_ is the [Ca^2+^]_c_ activation constant.

Endogenous PLC on PM (3 # μm^-2^) was evaluated for a transformed human kidney cell line (tsA-201) whose dimensions are close to that of the β-cell [200, 216] and we used this value for PLC_pt_ evaluation (Table 4).

#### IP_3_ dynamics

The generation of IP_3_ is determined by the hydrolysis rate of PIP_2_. Because IP_3_ molecules diffuse through the cytosol, we refer to IP_3_ production and degradation in terms of concentration (in μM) instead of the area density of molecules on PM. Different mechanisms seem to be available in the β-cells for degradation of IP_3_, however, we assume that IP_3_ is degraded at a rate proportional to the concentration of IP_3_. Then we used the simplest model of IP_3_ dynamics from [33]) but it was modified to consider the processes of IP_3_ production on PM.

$${\text{d IP}}_{3}\;/\;\text{dt}={\text{V}}_{\text{PL}}\;{\text{f}}_{\text{Nc}}\;{\text{f}}_{\text{PIP}2}-{\text{k}}_{\text{dIP}3}\;{\text{IP}}_{3}$$ (82)

where f_Nc_ is the coefficient to convert from # μm^-2^ to μM (Eq 1), *k*_dIP3_ is the rate constant of IP_3_ degradation.

#### DAG dynamics

DAG is the product of PLC-catalyzed breakdown of phosphoinositides, stimulates by PLC where it is produced stoichiometrically with IP_3_. However, DAG is bound with PM. Model of the DAG dynamics was developed similarly to the IP3 model. Coefficients from model [201] for native tsA201 cells were used (Table 6).

$$\text{d DAG}\;/\;\text{dt}={\text{V}}_{\text{PL}}\;{\text{f}}_{\text{PIP}2}-{\text{k}}_{\text{dDAG}}\;\text{DAG}$$ (83)

where *k*_dDAG_ is the coefficient of DAG degradation

#### DAG dependent PKC activation

Binding of DAG to the regulatory units of PKC results in release of the catalytic units from PKC and its activation. Relative PKC activation by DAG was described similarly to work [202] as equation:

$${\text{d PKC}}_{\text{a}}/\;\text{dt}={\text{k}}_{\text{PKC}}\;\text{DAG}\;*\;{\text{PKC}}_{\text{i}}-{\text{k}}_{\text{PKCr}}\;{\text{PKC}}_{\text{a}},$$ (84)

$${\text{PKC}}_{\text{i}}={\text{PKC}}_{\text{t}}\;-\;{\text{PKC}}_{\text{a}},$$ (85)

where PKC_t_ = 1

PKC_a_ is the relative concentration of active PKC, PKC_i_ is the relative concentration of inactive PKC, PKC_t_ is the total relative concentration of active and inactive PKC, k_PKC_ and k_PKCr_ are the forward backward rate constants. t_1/2_ was evaluated as 204 s^-1^ [202] (k_PKCr_ = 0.0034 s^-1^).

### Ca^2+^ dynamics

Cytoplasmic Ca^2+^ dynamics was modeled at the whole cell level. We included only fluxes through Ca^2+^ channels on PM, Ca^2+^ pumps on PM and ER and Ca^2+^ flux from ER. Then the free cytoplasmic Ca^2+^ ([Ca^2+^]_c_ or Cac in computational program) dynamics can be modeled by the following equations:

$$\frac{{\text{d}[{\text{Ca}}^{2+}]}_{\text{c}}}{\text{dt}}=\frac{{\text{f}}_{\text{cf}}}{{\text{V}}_{\text{c}}}\;(\frac{-I_{\text{Ca}}-\;I_{\text{SOC}}-2I_{\text{PCa}}}{2\;\text{F}}+J_{\text{rel}}-\;J_{\text{ser}}\;)-\;{\text{k}}_{\text{sg}}\;{\left[{\text{Ca}}^{2+}\right]}_{\text{c}}$$ (86)

where f_cf_ is the fraction of free Ca^2+^ in cytoplasm, F is Faraday’s constant, V_c_ is the effective volume of the cytosolic compartment, and k_sg_ is the coefficient of Ca^2+^ sequestration rate. Total Ca^2+^ current on PM include the current through the voltage-dependent Ca^2+^ channels—*I*_Ca_ (Eq 18); the store-operated current—*I*_SOC_ (Eq 21) and the PM Ca^2+^ ATP-ase pumps—*I*_PCa_ (Eq 23). *J*_ser_ is the Ca^2+^ flux from the cytosol into the ER activated by Ca^2+^ ATP-ases (SERCA) (per cell); *J*_rel_ is the Ca^2+^ flux from the ER into the cytosol (per cell).

Ca^2+^ dynamics in ER ([Ca^2+^]_Er_ or CaER in computational program) can be modeled by the following equation:

$${\text{d}[{\text{Ca}}^{2+}]}_{\text{Er}}\;/\;\text{dt}={\text{f}}_{\text{er}}\;(J_{\text{ser}}-\;J_{\text{rel}})/\;{\text{V}}_{\text{ER}}$$ (87)

where f_er_ is the is the fraction of free Ca^2+^ in ER, V_ER_ is the effective volume of the ER compartment. A function of the Ca^2+^ ATP-ase on ER (per cell) was modeled using the usual expression [33]:

$$J_{\text{ser}}={\text{P}}_{\text{CaER}}{\left[{\text{Ca}}^{2+}\right]}_{\text{c}}{}^{2}\;/\;({\text{K}}_{\text{ser}}{}^{2}+{\left[{\text{Ca}}^{2+}\right]}_{\text{c}}{}^{2})$$ (88)

where P_CaEr_ is the maximum SERCA pump rate, K_ser_ is the [Ca^2+^]_c_ activation constant.

The total Ca^2+^ flux from the ER into the cytosol is given by

$$J_{\text{rel}}=({\text{k}}_{\text{mIP}}\;{\text{P}}_{\text{RIP}3}+{\text{k}}_{\text{leak}})\;({\left[{\text{Ca}}^{2+}\right]}_{\text{Er}}-{\left[{\text{Ca}}^{2+}\right]}_{\text{c}})$$ (89)

where k_mIP_ is the maximum permeability for IP_3_-activated channel, P_RIP3_ is the IP_3_ receptor channel open probability, k_leak_ is the coefficient of Ca^2+^ passive leak from the ER through unspecified channels.

#### IP_3_ Receptor

The simplest model of IP_3_ receptor (IP_3_R) activated by IP_3_ and Ca^2+^ was taken from Bertram and Sherman [203]. Additional possibility is an activation of IP_3_R by PKA. PKA-mediated phosphorylation leads to a direct increase in the sensitivity of the IP_3_ receptor toward IP_3_ without shifting its Ca^2+^ sensitivity. We modeled this effect as a decreased IP_3_R activation constant with PKA activation (function f_IPKA_).

$${\text{P}}_{\text{RIP}3}={\left(\frac{{\left[{\text{Ca}}^{2+}\right]}_{\text{c}}}{{\text{K}}_{\text{RPCa}}+{\left[{\text{Ca}}^{2+}\right]}_{\text{c}}}\right)}^{3}{\left(\frac{{\text{IP}}_{3}}{{\text{f}}_{\text{IPKA}}\;{\text{K}}_{\text{IP}3}+{\text{IP}}_{3}}\right)}^{3}{\left(\frac{{\text{d}}_{\text{inact}}}{{\text{d}}_{\text{inact}}+{\left[{\text{Ca}}^{2+}\right]}_{\text{c}}}\right)}^{3}$$ (90)

where

$${\text{f}}_{\text{IPKA}}=1/(\text{PKAa}+{\text{K}}_{\text{IP}3})$$

P_RIP3_ is the channel open probability, K_RPCa_ is the [Ca^2+^]_c_ activation constant, K_IP3_ is the IP_3_ activation constant, d_inac_ is the [Ca^2+^]_c_ inhibition constant at higher concentrations of Ca^2+^, k_IP3R_ is the PKAa activation coefficient. The first factor represents an activation by Ca^2+^, the second an activation by IP_3_, and the third an inactivation at high concentration of [Ca^2+^]_c_.

### Simulations

The model consists of a system of nonlinear ordinary differential equations describing the time rate of change in parameters. Parameter values and initial conditions (Tables 1–6) contain all the information necessary to carry out the simulations. These values were used in all simulations except where indicated otherwise. To calculate the steady-state cellular parameters, the model was allowed to run for at least 1000 s with no external stimulation.

Simulations were performed as noted previously using standard numerical methods and the software environment from “Virtual Cell” (see for example [25, 33]).

## Abbreviations

AC: adenylyl cyclase
AC_P_: PM bound AC
AC_c_: soluble cytoplasmic AC
AdR: adrenergic receptors
ER: endoplasmic reticulum
CAM: calmodulin
CM: computational model
CRA: Ca^2+^ release-activated nonselective cation channel
[Ca^2+^]_c_: cytoplasmic free Ca^2+^ concentration
[Ca^2+^]_ER_: Ca^2+^ concentration inside of endoplasmic reticulum
DAG: diacylglycerol
Epac: guanyl exchange protein directly activated by cAMP
ER: endoplasmic reticulum
GIP: glucose-dependent insulinotropic polypeptide
GLP-1: glucagon-like peptide-1
GIPR: glucose-dependent insulinotropic polypeptide receptor
GLP-1R: glucagon-like peptide-1 receptor
GPCR: G-protein-coupled receptor
GPR40: G-protein-coupled receptor 40
GSIS: glucose-stimulated insulin secretion
FFA: free fatty acid
FFAR1/GPR40: free fatty acid receptor
IP_3_: inositol 1,4,5-trisphosphate
IP_3_R: IP_3_ receptor
K_ATP_ channels: ATP-sensitive K^+^ channels
MR: muscarinic acetylcholine receptor
NALCN: nonselective cation channels
PDE: phosphodiesterase
PI: phosphatidylinositol
PIP: phosphatidylinositol 4-phosphate
PIP_2_: phosphatidylinositol 4,5-bisphosphate
PKA: protein kinase A
PKD: protein kinase D
PKC: protein kinase C
PLC: phospholipase C
PLC_P_: G-protein-coupled PLC localized on PM
PLC_c_: cytoplasmic PLC
PM: plasma membrane
P4P: phosphatidylinositol-4-phosphate
SERCA: sarco-(endo)plasmic reticulum Ca^2+^-ATPase
SOC: store operated-current
T2D: type 2 diabetes
VGCC: voltage-gated Ca^2+^ channel
V_p_: plasma membrane potential
